# Tuning Hsf1 levels drives distinct fungal morphogenetic programs with depletion impairing Hsp90 function and overexpression expanding the target space

**DOI:** 10.1371/journal.pgen.1007270

**Published:** 2018-03-28

**Authors:** Amanda O. Veri, Zhengqiang Miao, Rebecca S. Shapiro, Faiza Tebbji, Teresa R. O’Meara, Sang Hu Kim, Juan Colazo, Kaeling Tan, Valmik K. Vyas, Malcolm Whiteway, Nicole Robbins, Koon Ho Wong, Leah E. Cowen

**Affiliations:** 1 Department of Molecular Genetics, University of Toronto, Toronto, Ontario, Canada; 2 Faculty of Health Sciences, University of Macau, Macau SAR, China; 3 Department of Molecular and Cellular Biology, University of Guelph, Guelph, Ontario, Canada; 4 Infectious Disease Research Centre, Université Laval, Quebec City, Quebec, Canada; 5 Vanderbilt University School of Medicine, Nashville, Tennessee, United States of America; 6 Whitehead Institute for Biomedical Research, Cambridge, Massachusetts, United States of America; 7 Department of Biology, Concordia University, Montréal, Quebec, Canada; University College Dublin, IRELAND

## Abstract

The capacity to respond to temperature fluctuations is critical for microorganisms to survive within mammalian hosts, and temperature modulates virulence traits of diverse pathogens. One key temperature-dependent virulence trait of the fungal pathogen *Candida albicans* is its ability to transition from yeast to filamentous growth, which is induced by environmental cues at host physiological temperature. A key regulator of temperature-dependent morphogenesis is the molecular chaperone Hsp90, which has complex functional relationships with the transcription factor Hsf1. Although Hsf1 controls global transcriptional remodeling in response to heat shock, its impact on morphogenesis remains unknown. Here, we establish an intriguing paradigm whereby overexpression or depletion of *C*. *albicans HSF1* induces morphogenesis in the absence of external cues. *HSF1* depletion compromises Hsp90 function, thereby driving filamentation. *HSF1* overexpression does not impact Hsp90 function, but rather induces a dose-dependent expansion of Hsf1 direct targets that drives overexpression of positive regulators of filamentation, including Brg1 and Ume6, thereby bypassing the requirement for elevated temperature during morphogenesis. This work provides new insight into Hsf1-mediated environmentally contingent transcriptional control, implicates Hsf1 in regulation of a key virulence trait, and highlights fascinating biology whereby either overexpression or depletion of a single cellular regulator induces a profound developmental transition.

## Author summary

For human pathogens, the capacity to respond to elevated temperature is required for survival, with elevated temperature in the form of fever as a conserved host response to defend against infection. One of the leading fungal pathogens of humans in *Candida albicans*, which is capable of growing in both a yeast and filamentous state. The ability to transition between these forms is a key virulence trait, and one that is highly temperature-dependent. A pivotal regulator of filamentous growth is the temperature-responsive molecular chaperone Hsp90, which has complex relationships with the transcription factor Hsf1. Although Hsf1 regulates changes in gene expression in response to heat shock, its impact on morphogenesis remains unknown. Here, we uncover an intriguing phenomenon whereby overexpression or depletion of *C*. *albicans HSF1* induces morphogenesis. We observe that *HSF1* depletion compromises Hsp90 function, thereby driving filamentation. In contrast, *HSF1* overexpression induces a dose-dependent expansion of its transcriptional targets that drives overexpression of positive regulators of filamentous growth. This work illuminates novel mechanisms through which tuning the levels of an environmentally contingent transcription factor drives a key developmental program.

## Introduction

Microorganisms that colonize mammals are continually challenged with a myriad of environmental stimuli, such as osmotic imbalances, oxidative stresses, nutrient limitation and temperature fluctuations, with fever as one of the most ubiquitous responses to infection. The capacity to sense and respond to temperature change is critical for pathogens to survive, and the circuitry governing responses to thermal stress is one of the most conserved responses in nature[1]. For fungal pathogens, temperature sensing also has a profound impact on developmental programs that are crucial for virulence. This is exquisitely clear for thermally dimorphic fungi, including species of *Histoplasma*, *Blastomyces*, *Coccidioides* and *Paracoccidiodes*, which grow as non-pathogenic filamentous molds in the soil and respond to the temperature increases of a mammalian host by switching to a pathogenic yeast form[2].

Although the opportunistic fungal pathogen *Candida albicans* resides within warm-blooded mammals and occupies a thermally buffered niche, it has retained the heat shock response[3, 4] and temperature influences diverse aspects of its biology including mating, phenotypic switching, and drug resistance[5]. Temperature also has a profound effect on the transition between distinct morphological states, including the yeast form and filamentous pseudohyphal and hyphal forms[6]. The capacity to transition between morphotypes is strongly correlated with virulence[7, 8], as both forms play critical roles in infection. The transition from yeast to filamentous growth is induced by diverse host relevant environmental cues, including exposure to serum, nutrient limitation, and neutral pH, all of which require the physiologically relevant temperature of 37°C[5, 6]. Response to these morphogenetic cues is regulated by complex signaling cascades, which include many transcription factors that positively regulate filamentation, such as Efg1, Cph1, Flo8[5, 6], Ume6[9, 10] and Brg1[11], as well as repressors of morphogenesis, such as Tup1 and Nrg1[5, 6]. Although filamentation in response to different cues can depend upon distinct genetic circuitry and culminate in contrasting cellular and sub-cellular phenotypes, filamentation in response to most cues is accompanied by a common global transcriptional remodeling, which includes the expression of a suite of filament-specific genes that encode adhesin proteins such as Als3 and Als5, secreted aspartyl proteases such as Sap4 and Sap5, cyclins such as Hgc1, and the hyphal wall protein Hwp1[12, 13]. Although genetic screens have revealed many genes that influence filamentation[5–8, 14], the molecular basis of how environmental signals such as temperature are sensed and transduced to activate this developmental program remains largely enigmatic.

A fascinating example of temperature sensing relevant to morphogenesis involves the coordinated and rapid induction of a suite of cellular heat shock proteins (HSPs) that enable adaptation to thermal insults. At the core is Hsp90, an essential and conserved molecular chaperone that stabilizes diverse client proteins integral to many signaling pathways[15]. Hsp90 represses *C*. *albicans* morphogenesis via the cyclic AMP-protein kinase A (cAMP-PKA) signaling pathway[16, 17], which positively regulates filamentation in response to diverse inducing cues. The elevated temperature normally required for filamentation creates a state of cellular stress that exceeds the functional capacity of Hsp90 to chaperone client proteins, thereby relieving Hsp90-mediated repression of the filamentation program[17, 18]. Compromise of Hsp90 function genetically or pharmacologically also relieves repression of cAMP-PKA signaling and induces a morphological transition from yeast to filamentous growth in the absence of an inducing cue or elevated temperature[17]. Filamentation in response to Hsp90 inhibition is dependent on most components of the cAMP-PKA pathway[17], interactions with the cell cycle kinase Cdc28[19], and signaling through the cyclin Pcl1, cyclin-dependent kinase Pho85 and transcription factor Hms1[20]. Further, Hsp90 function is regulated by complex interactions with co-chaperones that regulate its ATPase activity and mediate interactions with client proteins, and by post-translational modifications such as phosphorylation and acetylation[15, 21], which can have profound effects on cellular responses to thermal stress and on morphogenesis[16, 22].

Global transcriptional remodeling in response to elevated temperature is orchestrated by the essential heat shock transcription factor, Hsf1. Hsf1 is a conserved temperature sensor, whose expression and activity are elevated in response to acute increases in temperature, in order to enable crucial cellular responses to thermal stress[3, 23]. In *C*. *albicans* and related yeast species, Hsf1 is constitutively trimerized and located in the nucleus where it drives the basal expression of *HSP90* and other chaperone genes and is poised to induce their expression upon heat shock[3, 4, 24]. Hsp90 physically interacts with Hsf1 and represses its function, such that compromising Hsp90 function leads to activation of Hsf1 and induction of the heat shock response[25]. We recently established that *C*. *albicans* Hsf1 binds to an expanded set of targets in response to heat shock, and engages not only with genes involved in protein folding, but also genes important for filamentation, pathogenesis, adhesion and biofilm formation[4]. This is consistent with findings that mutations in *HSF1* that block its activation prevent thermal adaptation and attenuate virulence in a mouse model of systemic disease[26], although Hsf1 has yet to be implicated in morphogenesis. *C*. *albicans* Hsf1 binds to distinct motifs in nucleosome-depleted promoter regions that resemble conserved Hsf1 binding sites, referred to as Heat Shock Elements (HSEs), which are found in other eukaryotes including *Saccharomyces cerevisiae*[24], *Cryptococcus neoformans*[27], *Drosophila* species[28] and humans[29]. Importantly, there are 4,879 *C*. *albicans* genes with an Hsf1 consensus sequence but no detectable Hsf1-binding under basal or heat shock conditions, suggesting that Hsf1 binds to distinct targets in response to different environmental conditions[4]. As an environmentally contingent transcriptional regulator of cellular circuitry, Hsf1 is poised to regulate temperature-dependent developmental programs in *C*. *albicans*.

Here, we establish that tuning the expression level of *HSF1* has a profound impact on *C*. *albicans* morphogenesis, such that depletion or overexpression of *HSF1* is sufficient to induce a transition from yeast to filamentous growth in the absence of any external cues. We demonstrate that the filaments induced by *HSF1* depletion or overexpression are structurally distinct, are contingent upon different genetic circuitry, and are induced through independent mechanisms. Our findings support the model that *HSF1* depletion induces filamentation by compromising Hsp90 function, independent of its role in regulating *HSP90* transcription. *HSF1* overexpression does not impact Hsp90 function, but rather induces filamentation by driving the overexpression of positive regulators of morphogenesis and repressing the expression of negative regulators of morphogenesis. For example, global ChIP-seq and RNA-seq analyses reveal that *HSF1* overexpression expands the set of bona fide targets, thereby influencing the expression of many morphogenetic regulators, including the positive regulators Ume6 and Brg1, and the negative regulator Nrg1. Reducing the ambient temperature abolishes filamentation induced by *HSF1* overexpression, but does not affect filamentation induced by *HSF1* depletion. This work provides new insight into Hsf1-mediated environmentally contingent transcriptional control, implicates Hsf1 in regulation of a key virulence trait, and highlights fascinating biology whereby either overexpression or depletion of a single cellular regulator induces a profound developmental transition.

## Results

### *HSF1* overexpression and depletion induce filamentation through distinct mechanisms

**Fig 1:**
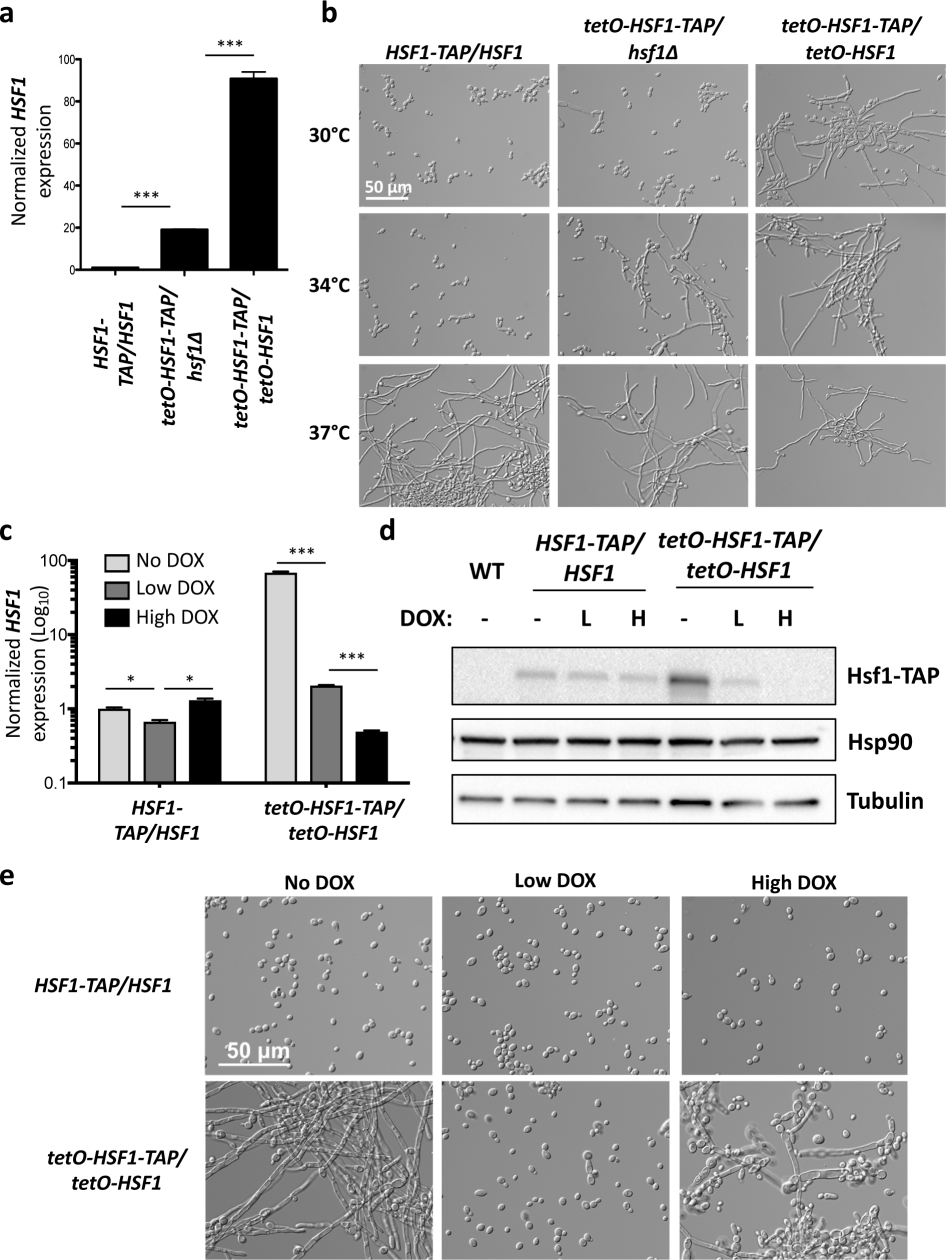
Overexpression or depletion of *HSF1* induces filamentation. a) *HSF1* levels can be overexpressed to different levels by placing one or both alleles of *HSF1* under the control of the tetracycline-repressible promoter, *tetO*. Strains were grown in the absence of doxycycline (DOX) to mid-log phase. *HSF1* transcript levels were measured by quantitative RT-PCR and normalized to *ACT1* and *GPD1*. Data are means +/- standard error of the means for triplicate samples. *** indicates P value <0.005, unpaired t test. b) Overexpression of *HSF1* can bypass the temperature requirement for morphogenesis. Strains were grown in the absence of DOX for 4 hours at the indicated temperature. c) The *tetO-HSF1-TAP/tetO-HSF1* strain can be used to monitor the effect of both *HSF1* overexpression and depletion. *HSF1* is overexpressed in the *tetO-HSF1-TAP/tetO-HSF1* strain when grown in the absence of DOX (No DOX). *HSF1* expression is lowered when the strain is grown in the presence of 0.1 μg/mL DOX (Low DOX), and is depleted when grown with 20 μg/mL DOX (High DOX). *HSF1* transcript levels were measured by quantitative RT-PCR and were normalized to *ACT1* and *GPD1*. Data are means +/- standard error of the means for triplicate samples and are graphed on a log_10_ scale. *** indicates P value <0.005, * indicates P value <0.05, unpaired t test. d) Hsf1 protein levels are overexpressed when the *tetO-HSF1-TAP/tetO-HSF1* strain is grown in the absence of DOX, reduced when grown in 0.1 μg/mL DOX (Low DOX, labelled L), and depleted when grown in 20 μg/mL DOX (High DOX, labelled H). WT indicates the wild type, untagged control. Tubulin levels serve as loading control. e) *HSF1* overexpression or depletion induces filamentation in the absence of an inducing cue. Strains were grown in the presence of no DOX, 0.1 μg/mL DOX (Low DOX), or 20 μg/mL DOX (High DOX) at 30°C.

To investigate the role of Hsf1 in *C*. *albicans* morphogenesis, we engineered a strain in which one allele of *HSF1* is deleted and the remaining allele is regulated by the tetracycline-repressible promoter *tetO* (*tetO-HSF1/hsf1Δ*), which is repressed by tetracycline or the analog doxycycline (DOX). As *HSF1* is generally expressed at low levels, replacing its native promoter with the strong *tetO* promoter caused *HSF1* overexpression in the absence of DOX **(Fig 1A)**. Although *C*. *albicans* morphogenesis is normally contingent upon a temperature of 37°C, we observed that overexpression of *HSF1* in the *tetO-HSF1/hsf1Δ* strain enabled filamentation at 34°C **(Fig 1B)**. In a strain where both alleles of *HSF1* were regulated by *tetO* (*tetO-HSF1/tetO-HSF1*), the magnitude of *HSF1* overexpression in the absence of DOX was much greater **(Fig 1A)**, completely bypassing the requirement for elevated temperature and inducing filamentation even at 30°C in the absence of an inducing cue **(Fig 1B)**. Filamentation is not a general response to overexpressing genes in this system, as overexpression of other genes, including the morphogenetic regulator *HSP90*, did not promote filamentous growth **(S1A Fig)**. Contrary to findings in *S*. *cerevisiae* where *HSF1* overexpression causes severe growth defects and toxicity[30, 31], overexpression of *HSF1* in *C*. *albicans* did not cause a defect in growth or viability **(S1B Fig)**. Thus, *HSF1* overexpression is sufficient to drive morphogenesis, and *HSF1* levels modulate the temperature-dependence of an important developmental program.

To further explore the relationship between *HSF1* levels and filamentation, we grew the *tetO-HSF1/tetO-HSF1* strain in varying concentrations of DOX to repress *HSF1* expression. As expected, when the *tetO-HSF1/tetO-HSF1* strain was grown in the absence of DOX, *HSF1* transcript and protein levels were dramatically overexpressed **(Fig 1C and 1D)**, causing robust filamentation at 30°C **(Fig 1E)**. When grown in a very low concentration of DOX (0.01 μg/mL), *HSF1* levels were reduced to an intermediate level of overexpression that still promoted filamentous growth **(S2 Fig)**. Filamentation induced by *HSF1* overexpression was reversible, as growth in a low concentration of DOX (0.1 μg/mL) reduced *HSF1* expression to a similar level as observed in the wild-type strain (**Fig 1C, Fig 1D and S2 Fig),** and the cells grew in the yeast form **(Fig 1E and S2 Fig)**. Remarkably, when the *tetO-HSF1/tetO-HSF1* strain was grown in the presence of a ten-fold higher concentration of DOX (1 μg/mL) some filamentation was observed **(S2 Fig)**, and filamentation was more robust when the strain was grown in high concentrations of DOX (10–20 μg/mL) that lead to a significant reduction in *HSF1* levels (**Fig 1C, Fig 1D, Fig 1E** and **S2 Fig)**. Although Hsf1 is essential in *C*. *albicans*[3, 8, 26], depletion of *HSF1* with 20 μg/mL DOX in these conditions had only a minor impact on viability **(S1B Fig)**. We observed that overexpression or depletion of *HSF1* induced filamentation in multiple strain backgrounds, confirming the robustness of the phenotypes **(S3 Fig)**. Analysis of published RNA sequencing (RNA-seq) data of wild-type *C*. *albicans* grown in diverse environmental conditions revealed that *HSF1* expression is highly responsive to the environment and varies over 100-fold across experiments **(S4A Fig)**. In particular, *HSF1* levels are transcriptionally upregulated upon a 30°C to 42°C heat shock, as well as during biofilm growth **(S4B Fig)**. This upregulation leads to a level of *HSF1* that is similar to that observed in the *tetO-HSF1/hsf1Δ* strain when grown in the absence of DOX **(S4B Fig)**, which we demonstrated promotes filamentation at 34°C **(Fig 1B)**. Together, this defines a fascinating paradigm in which tuning the levels of a single environmentally-responsive transcriptional regulator drives *C*. *albicans* morphogenesis.

**Fig 2:**
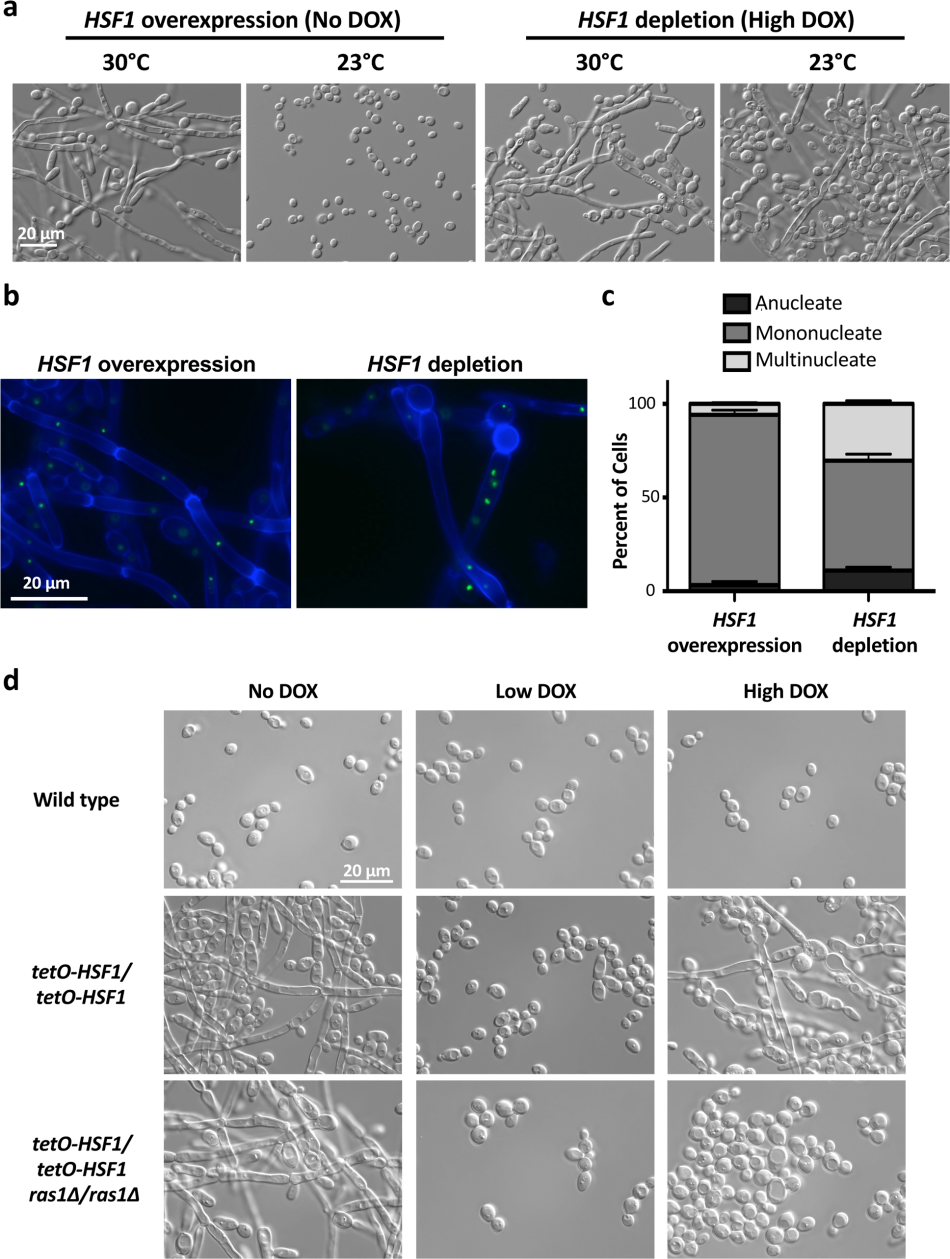
*HSF1* overexpression and depletion induce filaments that differ in their morphology, nuclear content and dependence on Ras1. a) Reduced temperature blocks filamentation in response to *HSF1* overexpression but not in response to *HSF1* depletion. Strains were grown in the absence or presence of high levels of DOX (20 μg/mL) at the indicated temperature. b) Filaments induced by *HSF1* overexpression and depletion have distinct features. Cell walls and septa were visualized using calcofluor white and the nuclei were visualized using a strain with the nucleolar protein, Nop1, GFP tagged. c) Filaments induced by *HSF1* depletion have a significant increase in the percentage of cells that are multinucleate compared to filaments induced by *HSF1* overexpression. The number of nuclei in at least 300 cells were counted for each condition, for two biological replicates. Means are graphed with the error bars displaying the standard error of means. Unpaired t-test indicates a significant difference in percentage of multinucleate cells, P value is 0.0056. d) Filaments induced by *HSF1* depletion, but not overexpression, are dependent on the GTPase Ras1. Strains were grown in rich medium with 80 mg/L uridine added, in the presence of no DOX, 0.1 μg/mL DOX (Low DOX), or 20 μg/mL DOX (High DOX) at 30°C.

Hsf1 is exquisitely responsive to temperature, as its expression and activity increase in response to temperature upshifts, thereby enabling thermal adaptation[23]. Given that modulating *HSF1* expression altered the requirement of elevated temperature for morphogenesis **(Fig 1B)**, we hypothesized that further reduction in temperature might impair filamentation in response to altered *HSF1* levels. To test this, we assayed whether filamentation induced by overexpression or depletion of *HSF1* was maintained at temperatures below 30°C. Filamentation in response to *HSF1* overexpression was completely blocked at 23°C, but filamentation in response to *HSF1* depletion was maintained **(Fig 2A)**. The temperature dependence of filamentation induced by *HSF1* overexpression was striking and distinct from the robust filamentation observed at 23°C in response to depletion of *HSP90*, deletion of the transcriptional repressors *TUP1* and *NRG1*, or overexpression of the positive regulators of filamentation *UME6* and *BRG1* **(S5 Fig)**. This suggests that Hsf1 activity is strongly influenced by temperature, with profound effects on its capacity to induce morphogenesis.

We further explored the morphology of filaments induced by *HSF1* overexpression and depletion using the GFP-tagged nucleolar protein Nop1 to visualize the nuclei and calcofluor white to label the septa and cell wall[32]. When *HSF1* was overexpressed, filaments formed parallel cell walls with distinct, mononucleate compartments defined by regularly spaced septa **(Fig 2B)**. In contrast, *HSF1* depletion induced filaments with variable widths, less-defined compartments harboring sporadically spaced septa, and often anucleate or multinucleate compartments **(Fig 2B)**. Quantification of the number of nuclei per cellular compartment confirmed that *HSF1* depletion induced filaments with a significant increase in multinucleate cells compared to filaments induced by *HSF1* overexpression **(Fig 2C)**. This is consistent with findings that *HSF1* depletion in *S*. *cerevisiae* leads to large, amorphous cells with 4C DNA content[33, 34]. Thus, we observed structural differences between the filaments induced by *HSF1* overexpression and depletion, suggesting that the mechanisms underpinning the cellular responses are distinct.

Next, we assessed whether filamentation in response to *HSF1* overexpression and depletion is dependent on the same genetic circuitry. To do so, we engineered *tetO-HSF1/tetO-HSF1* strains in a *RAS1* homozygous deletion mutant background. Ras1 is a GTPase that regulates cAMP-PKA signaling and is a critical regulator of filamentation in response to many cues[5, 17, 35]. Intriguingly, loss of Ras1 completely blocked filamentation induced by *HSF1* depletion but did not affect filamentation induced by *HSF1* overexpression **(Fig 2D)**. Ras1 remained necessary for filamentation induced by *HSF1* depletion even when the *tetO-HSF1/tetO-HSF1* strain was grown in a higher concentration of DOX (50 μg/mL), confirming that the block in filamentation is not a result of insufficient *HSF1* depletion **(S6 Fig)**. Taken together, we establish that overexpression of *HSF1* induces filaments that differ from those induced by depletion of *HSF1* in terms of their temperature dependence, cell biology and genetic requirements.

### *HSF1* depletion induces filamentation by compromising Hsp90 function

We focused first on elucidating the mechanism through which *HSF1* depletion induces filamentation. We confirmed that *HSF1* depletion induced filamentation when *HSF1* is repressed in the *tetO-HSF1/hsf1Δ* strain in the presence of DOX and with the independent *MAL2p* repressible promoter system, which is repressed when cells are grown in the presence of glucose (**S7A Fig)**. The *tetO-HSF1/hsf1Δ* strain was used for our mechanistic studies as there are profound changes in physiology associated with switching carbon sources, and the concentrations of DOX required for this system are phenotypically neutral. Further, this strain grows in the yeast form in the absence of DOX **(Fig 1B** and **S7A Fig)** simplifying the genetic system to study the mechanisms governing filamentation upon *HSF1* depletion. To provide an initial portrait of transcriptional changes in response to *HSF1* depletion, we performed comparative microarray analysis of the *tetO-HSF1/hsf1Δ* strain grown in the absence or presence of 20 μg/mL of DOX to deplete *HSF1*. This identified 1,346 genes whose expression were altered by at least 1.5-fold upon *HSF1* depletion (P value < 0.05; **S1 Data**), with GO term enrichments for oxidation-reduction processes and many processes related to morphogenesis **(S7B Fig** and **S1 Data)**. Gene Set Enrichment Analysis (GSEA) was employed to identify transcriptional profiles with significant correlation to the microarray profile induced upon *HSF1* depletion. This analysis identified a significant correlation (P value < 0.0001) between the profile of genes upregulated upon *HSF1* depletion with profiles of genes upregulated in filaments induced by serum at 37°C[12], treatment with cell cycle inhibitors[36], and treatment with geldanamycin[20], an Hsp90 inhibitor that binds with high affinity to the unusual Hsp90 ATP binding pocket[37] **(S7C Fig, S7D Fig** and **S1 Data)**. This suggests that depletion of *HSF1* induces a core transcriptional program that is common to filamentation in response to diverse cues.

**Fig 3:**
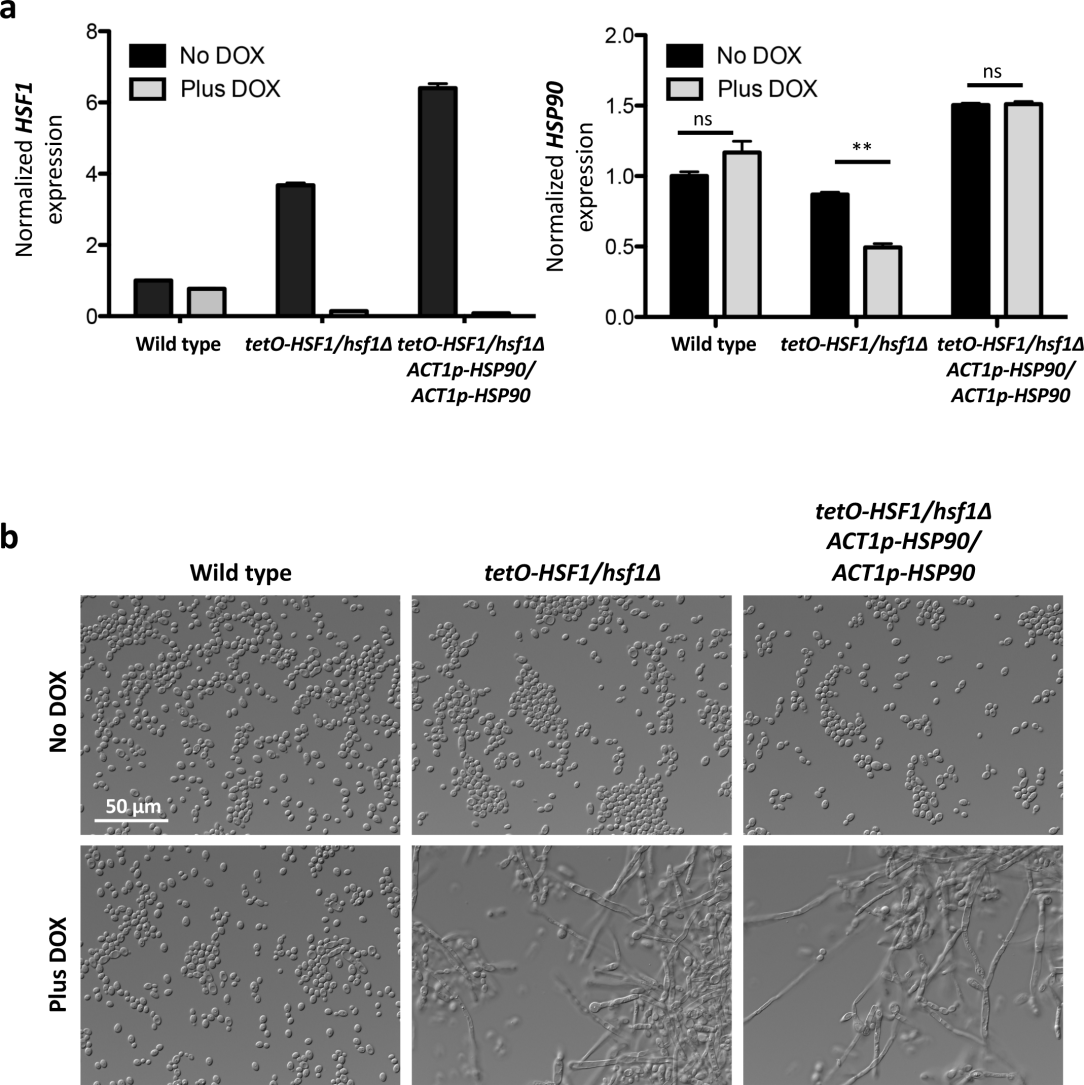
*HSF1* depletion induces filamentation independently of changes in *HSP90* expression. Strains were grown in the absence or presence of 20 μg/mL DOX in rich medium at 30°C. a) A strain was engineered where *HSP90* is under the control of a constitutive promoter, *ACT1p*, in the *tetO-HSF1/hsf1Δ* background. In the *ACT1p-HSP90* strain, *HSF1* levels were depleted (left panel) with DOX without altering *HSP90* expression (right panel). *HSF1* and *HSP90* transcript levels were normalized to *ACT1* and *GPD1*. Data are means +/- standard error of the means for triplicate samples. ** indicates P value <0.01, ns indicates no significant difference, unpaired t test. b) *HSF1* depletion induces filamentation independently of changes in *HSP90* expression.

**Fig 4:**
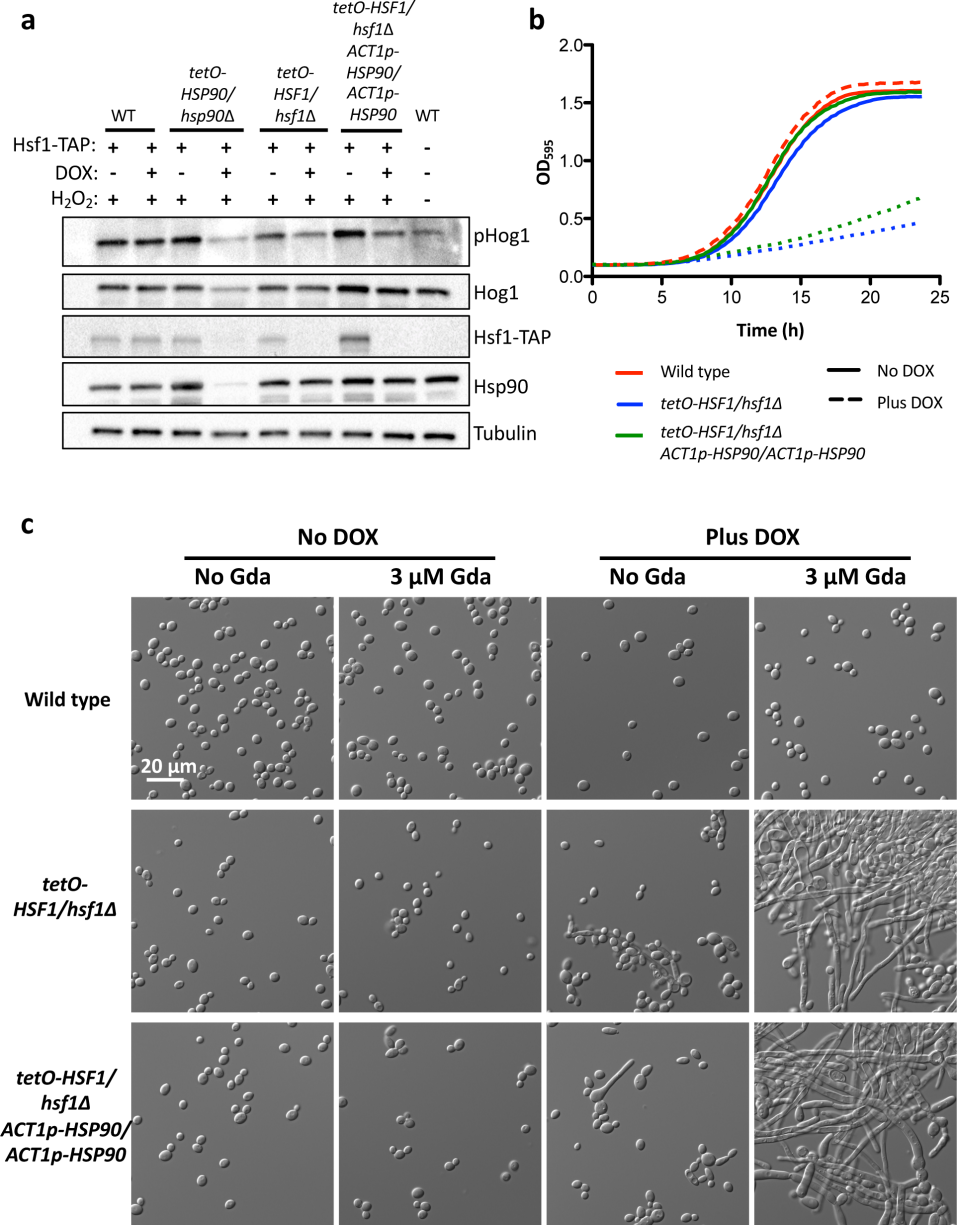
*HSF1* depletion induces filamentation by compromising Hsp90 function, independently of changes in *HSP90* expression. a) Western blot analysis was performed to assay if *HSF1* depletion compromises Hsp90 function by monitoring the Hsp90 client protein Hog1. Strains were grown in the absence or presence of 20 μg/mL DOX. Cells were treated with 5 mM hydrogen peroxide (H_2_O_2_) for 10 minutes to induce oxidative stress before protein extraction. Depletion of Hsf1 reduces the levels of phosphorylated Hog1 (pHog1) but not total Hog1 levels, even in the isogenic strain with constitutive *HSP90* expression. Tubulin levels serve as loading control. WT indicates the wild type control. Experiment was performed in biological quadruplicate and a representative image is shown, with the western blots for the additional replicates shown in **S8 Fig.** b) *HSF1* depletion causes a hypersensitivity to the Hsp90 inhibitor geldanamycin, even when *HSP90* expression is constitutive and independent of Hsf1. Growth curves were generated by measuring the optical density of cells grown in the absence or presence of 20 μg/mL DOX in the presence of a concentration of geldanamycin that does not inhibit the growth of the wild-type strain (3.13 μM). Optical density at 595 nm was measured every 15 minutes with a TECAN plate reader grown with high orbital shaking at 30°C. Experiment was performed in biological quadruplicate, with one representative graph shown. c) *HSF1* depletion causes filamentation at lower concentrations of geldanamycin (Gda) than is necessary to induce filamentation of the wild-type strain. Strains were grown in static conditions in the presence of no DOX or 20 μg/mL DOX, and in the presence of no geldanamycin (Gda) or 3.13 μM geldanamycin (Gda) at 30°C. In static growth conditions, 3.13 μM geldanamycin does not inhibit growth of the strains.

Based on the observations that Hsf1 regulates the expression of *HSP90* and co-chaperone genes important for Hsp90 function[3, 4], that Hsp90 regulates temperature-dependent morphogenesis[17, 18], and that filaments induced by compromised Hsp90 function and *HSF1* depletion have structural similarities including an increase in multinucleate cells[19], we hypothesized that filamentation in response to *HSF1* depletion is a consequence of reduced *HSP90* expression or function. To determine if *HSF1* induces filamentation via reduction of *HSP90* expression, we engineered a strain in which *HSF1* could be depleted without affecting *HSP90* levels. In the *tetO-HSF1/hsf1Δ* strain, we ectopically integrated *HSP90* under the control of the strong, constitutive promoter *ACT1p*, which is independent of Hsf1[4]. In these strains, DOX-mediated transcriptional repression of *HSF1* occurred without significant alterations in *HSP90* transcript or protein levels **(Fig 3A** and **Fig 4A)**. Genetic depletion of *HSF1* induced filamentation despite constitutive *HSP90* expression (**Fig 3B**), indicating that morphogenesis induced by depletion of *HSF1* occurs in a manner that is independent of *HSP90* expression. This suggests that *HSF1* depletion induces morphogenesis via impaired Hsp90 function or that it does so via circuitry that is independent of Hsp90.

**Fig 5:**
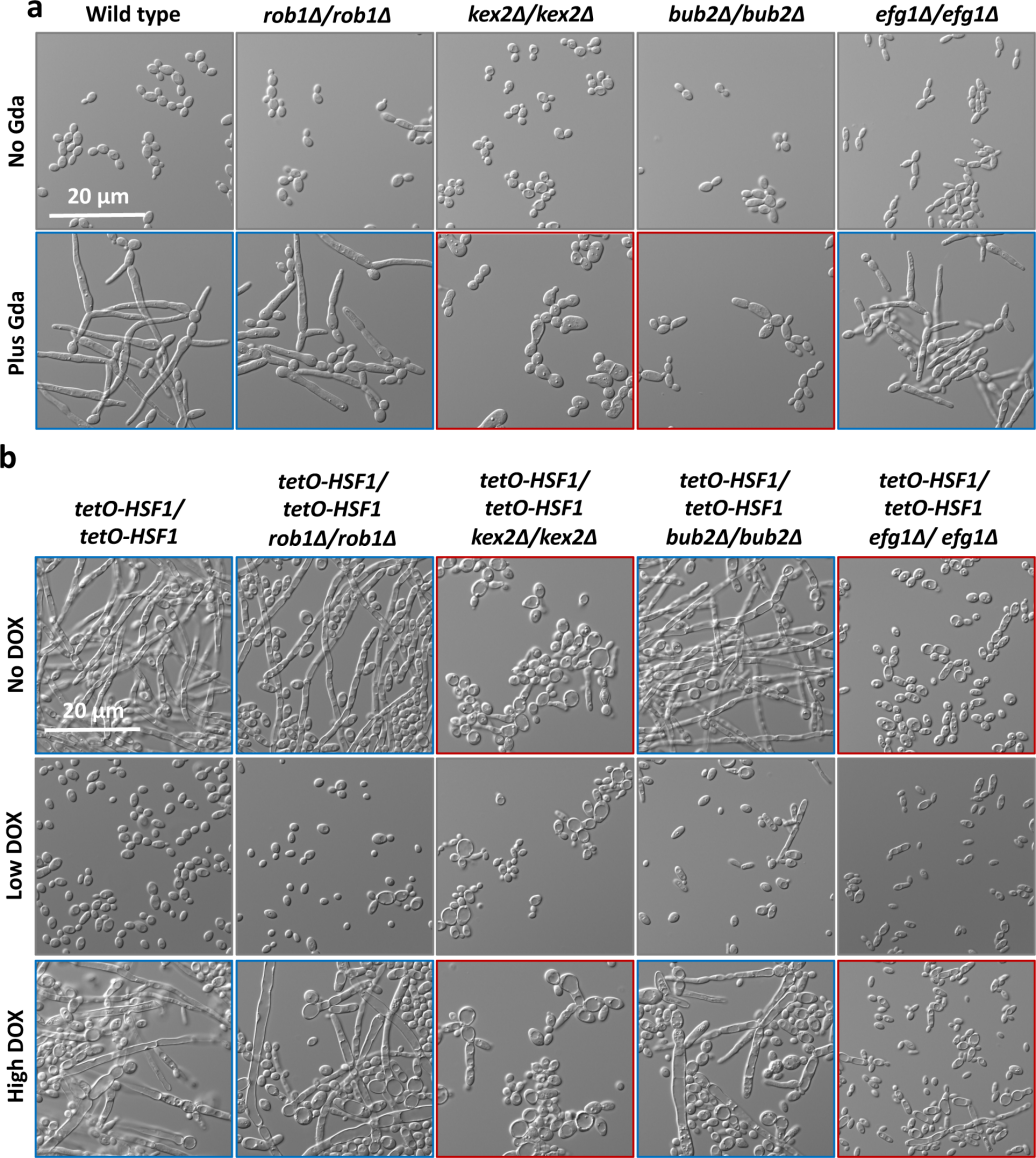
The genetic circuitry through which Hsf1 and Hsp90 regulate filamentation are distinct. a) Filaments induced by compromised Hsp90 function are not dependent on the transcription factors Rob1 or Efg1, but are dependent on the protease Kex2 and the cell cycle checkpoint protein Bub2. Cultures were grown in the absence or presence of 10 μM of geldanamycin (Gda) for 6.5 hours to inhibit Hsp90 function. b) Unlike filaments induced by compromised Hsp90 function, filaments induced by *HSF1* depletion are not dependent on Bub2 but are dependent on Efg1. Cultures were treated with no DOX, 0.1 μg/mL DOX (Low DOX), or 20 μg/mL DOX (High DOX) at 30°C. For both a) and b), blue outlines indicate that the homozygous deletion mutant of the morphogenetic regulator filaments to a comparable level as the non-mutant control and red outlines indicates that the mutant blocks filamentation.

To determine if Hsp90 function was compromised upon *HSF1* depletion, we monitored activation of the Hsp90 client protein Hog1, as Hsp90 enables activation of this mitogen activated protein kinase (MAPK)[38]. Depletion of *HSF1* caused a small but reproducible reduction in the levels of phosphorylated Hog1, even in the strain with constitutive *HSP90* expression **(Fig 4A** and **S8 Fig)**. *HSF1* depletion did not affect total Hog1 levels **(Fig 4A)**, highlighting that *HSF1* depletion does not have the same magnitude of impact as direct compromise of Hsp90 function. The reduction of phosphorylated Hog1 levels upon *HSF1* depletion are unlikely to be caused by direct transcriptional effect of Hsf1 on the Hog1 pathway, as the expression levels of *HOG1* and the upstream kinases *PBS2* or *SSK2*[39] are unaffected by *HSF1* depletion **(S1 Data)**. Together, this is consistent with the model that Hsf1 is required for proper Hsp90 function to enable the accumulation of activated Hog1. As a complementary approach to confirm that *HSF1* depletion compromises Hsp90 function, we monitored sensitivity to the Hsp90 inhibitor geldanamycin, as strains with compromised Hsp90 function are hypersensitive to this inhibitor[22]. We observed that *HSF1* depletion caused hypersensitivity to geldanamycin, even with constitutive *HSP90* expression **(Fig 4B)**. Strains with compromised Hsp90 function have also been observed to filament at lower concentrations of geldanamycin than the wild-type strain[40]. At a time point before *HSF1* depletion induces filamentation, *HSF1* depletion caused filamentation at a significantly lower concentration of geldanamycin (3.13 μM) than is necessary to induce filamentation in the wild-type strain (10 μM) **(Fig 4C** and **Fig 5A)**. Together, these results are consistent with a model in which *HSF1* depletion compromises Hsp90 function, which provides a mechanism through which *HSF1* depletion can induce filamentation.

Hsf1 might promote Hsp90 function by driving the transcription of Hsp90 co-chaperone genes that are important for chaperone function. Indeed, Hsf1 binds within the promoter region of six known Hsp90 co-chaperone genes in *C*. *albicans*: *AHA1*, *CDC37*, *CPR6*, *HCH1*, *SBA1*, and *STI1*[4]. We hypothesized that *HSF1* depletion could lead to reduction in the levels of these critical regulators of Hsp90 function, thereby inducing filamentous growth. To test the impact of reduced co-chaperone levels on filamentation, we generated deletion or depletion strains for each of the Hsf1-regulated Hsp90 co-chaperones and found that loss of any co-chaperone alone was not sufficient to induce filamentation **(S9 Fig)**. This suggests that *HSF1* depletion does not compromise Hsp90 function and drive filamentation through effects on a single co-chaperone, but rather, the effects could be mediated through multiple co-chaperones or through an additional regulator of Hsp90 function such as a kinase or deacetylase. Given that Hsf1 regulates the expression of many proteostasis genes including the Hsp70 genes *SSA1* and *SSA2*[4], it is also possible and consistent with findings in *S*. *cerevisiae*[41], that *HSF1* depletion could cause an accumulation of misfolded proteins, which could alter Hsp90 function indirectly by overwhelming the functional capacity of Hsp90.

To further explore the genetic circuitry governing filamentous growth upon *HSF1* depletion, we compared the genes necessary for filamentation in response to *HSF1* depletion with those genes necessary for filamentation upon inhibition of Hsp90 with geldanamycin. To do this, we generated *tetO-HSF1/tetO-HSF1* strains in homozygous deletion mutant backgrounds of genes important for filamentation, including *EFG1*[42], *BUB2*[19], *KEX2*[43], and *ROB1*[44]. We determined that the transcription factor Rob1 was dispensable for filamentation induced by compromised Hsp90 function and modulation of Hsf1 levels **(Fig 5)**. Surprisingly, unlike filamentation induced by compromised Hsp90 function[17, 20], we observed that filamentation induced by *HSF1* depletion was dependent on Efg1 **(Fig 5A** and **Fig 5B)**. We also observed that although the cell cycle checkpoint protein Bub2 was necessary for filamentation in response to geldanamycin[19] **(Fig 5A)**, it was dispensable for filamentation induced by depletion of *HSF1* **(Fig 5B)**. This suggests that although compromised Hsp90 function and *HSF1* depletion induce filamentation using some of the same genetic circuitry, including Ras1[17] **(Fig 2D)** and the protease Kex2 **(Fig 5)**, depletion of *HSF1* also governs filamentous growth through circuitry independent of Hsp90.

### *HSF1* overexpression drives expression of morphogenetic regulators

**Fig 6:**
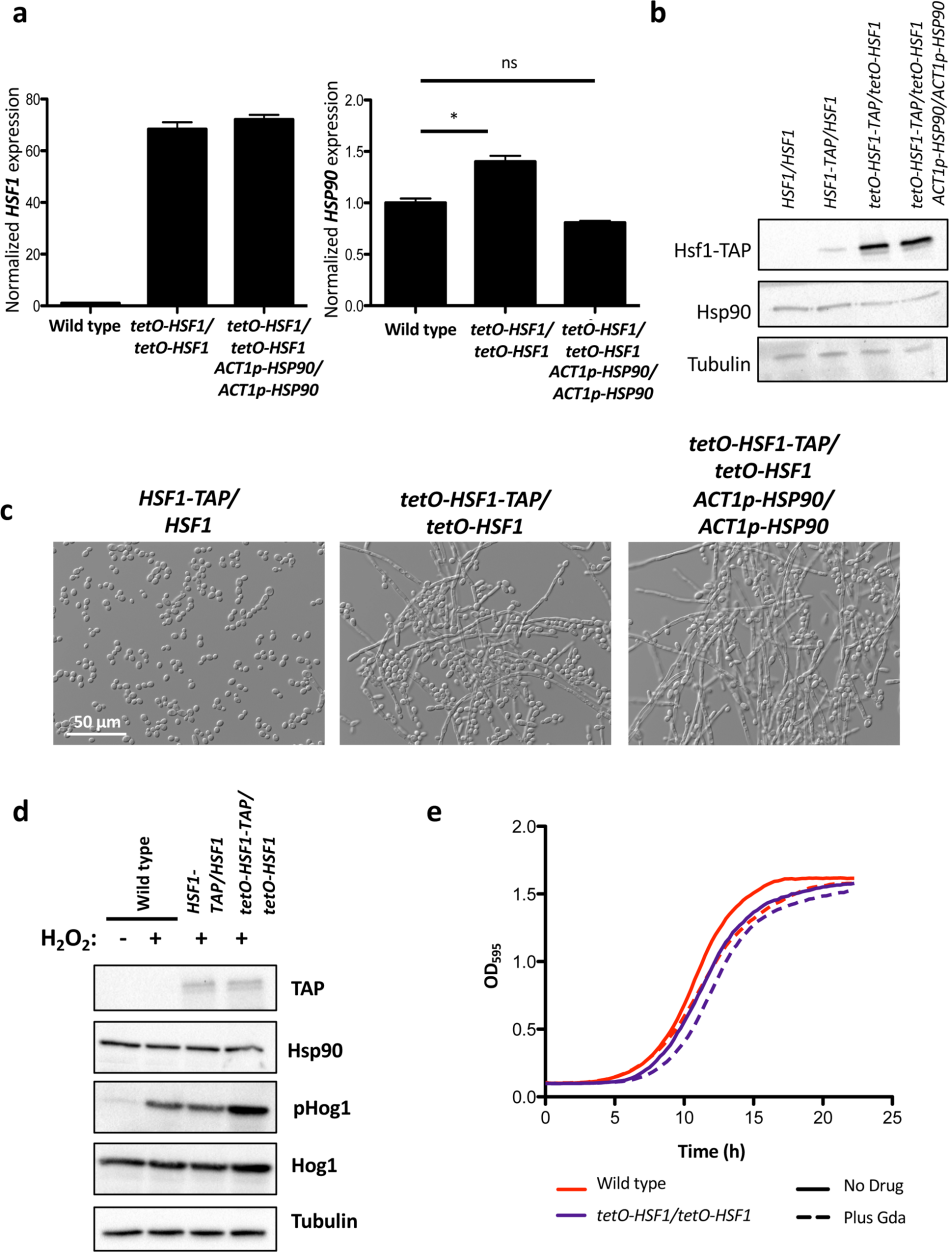
*HSF1* overexpression induces filamentation independently of changes in *HSP90* transcript or protein levels, and function. a) To monitor the effects of *HSF1* overexpression on filamentation independently of changes in *HSP90* expression, we engineered strains where *HSP90* is under the control of a constitutive promoter, *ACT1p*, in the *tetO-HSF1/tetO-HSF1* strain. *HSF1* and *HSP90* transcript levels were normalized to *ACT1* and *GPD1*. Data are means +/- standard error of the means for triplicate samples. In the *ACT1p-HSP90* strain, *HSF1* levels are overexpressed (left panel) but *HSP90* levels do not differ substantially from the wild-type levels (right panel). * indicates P value <0.05, ns indicates no significant difference, unpaired t test. b) Western blot analysis shows that overexpression of *HSF1* does not impact the levels of Hsp90 protein. Tubulin serves as loading control. c) *HSF1* overexpression induces filamentation independently of changes in *HSP90* expression. Strains were grown in the absence of DOX at 30°C. d) Western blot analysis was performed to assay if *HSF1* overexpression compromises Hsp90 function by monitoring the Hsp90 client protein Hog1. Cells were treated with 5 mM hydrogen peroxide (H_2_O_2_) for 10 minutes to induce oxidative stress before protein extraction. Overexpression of *HSF1* does not affect the stability of Hog1, nor does it block its activation. Tubulin serves as a loading control. e) *HSF1* overexpression does not cause hypersensitivity to the Hsp90 inhibitor geldanamycin (Gda). Growth curves were generated by measuring the optical density of cells grown in the absence and presence of 20 μg/mL DOX in the presence of 3.13 μM geldanamycin. Optical density measurements at 595 nm were taken every 15 minutes with a TECAN plate reader. Experiment was performed in biological quadruplicate, with one representative graph shown.

To determine if *HSF1* overexpression also influences filamentation through effects on Hsp90 expression or function, we generated *tetO-HSF1/tetO-HSF1* strains in which *HSP90* expression is independent of *HSF1* by placing *HSP90* under the control of the *ACT1* promoter. Despite stable *HSP90* transcript **(Fig 6A)** and protein levels **(Fig 6B)**, the strain filamented robustly in response to *HSF1* overexpression **(Fig 6C)**. Further, *HSF1* overexpression did not affect Hsp90 function, as *HSF1* overexpression did not cause a reduction in phosphorylated Hog1 or total Hog1 levels **(Fig 6D)**, or hypersensitivity to geldanamycin **(Fig 6E)**. Thus, *HSF1* overexpression does not influence morphogenesis through effects on Hsp90 expression or function.

**Fig 7:**
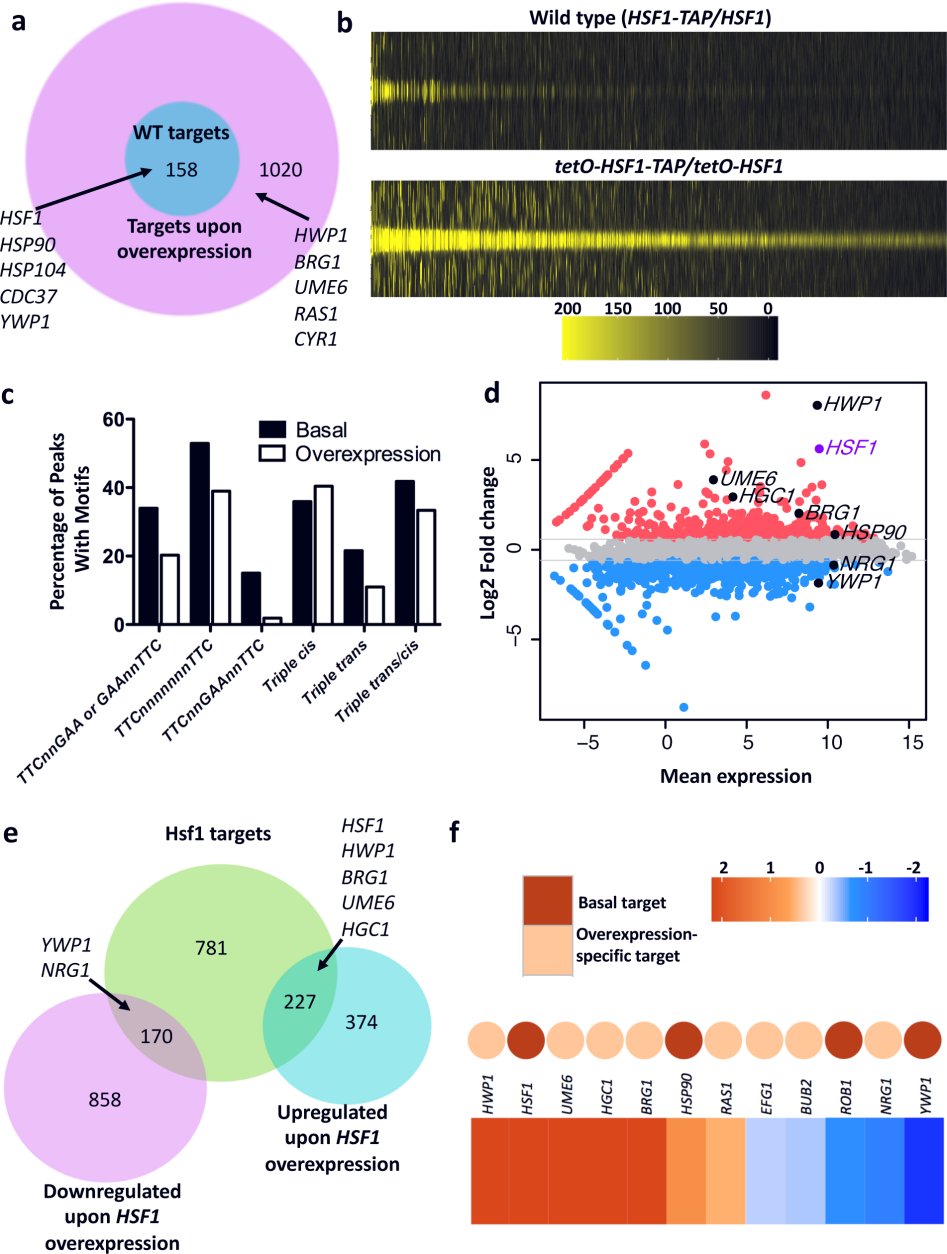
Hsf1 binds to an expanded set of taget genes upon overexpression, inducing the expression of positive regulators of filamentation. a) ChIP-seq was performed to determine the targets of Hsf1 in strains expressing basal (*HSF1-TAP/HSF1*) and overexpressed (*tetO-HSF1-TAP/tetO-HSF1*) levels of Hsf1, which identified an expansion of Hsf1 targets upon overexpression. Genes previously identified as Hsf1 targets and genes involved in *C*. *albicans* filamenation are indicated. b) Hsf1 signals at the summit of Hsf1 ChIP-seq peaks in wild-type (basal conditions, top) and *HSF1* overexpression (bottom) strains. Weak Hsf1 ChIP-seq signal can be seen at the overexpression-specific target sites under basal conditions, suggesting that Hsf1 also binds these sites under basal conditions albeit at a lower level. Legend shows Hsf1 ChIP-seq signal across a 2 kb window spanning Hsf1 binding summits identified by MACS analysis. c) Both basal and *HSF1* overexpression-specific targets contain the conserved heat shock element (HSE) binding sites. The percentage of ChIP-seq peaks with the HSE motifs TTCnnGAA, TTCn^7^TTC, TTCnnGAAnnTTC, or variations of the motifs termed triple cis motifs (GAA n^0-3^ GAA n^0-3^ GAA), triple trans motifs (GAA n^0-3^ TTC n^0-3^ GAA) or triple trans/cis motifs (TTC n^0-3^ GAA n^0-3^ GAA, GAA n^0-3^ GAA n^0-3^ TTC, TTC n^0-3^ TTC n^0-3^ GAA, GAA n^0-3^ TTC n^0-3^ TTC) are shown for basal and overexpression-specific targets. d) MA plot showing difference in gene expression between wild-type and *HSF1* overexpression strains. Genes more than 1.5-fold up-regulated in the *HSF1* overexpression strain as compared to the wild-type strain are shown in red, whereas genes more than 1.5-fold down-regulated are shown in blue. Genes of interest based on their roles in filamentation are indicated. *HSF1* is highlighted in purple. e) A Euler diagram depicting the overlap between differentially regulated genes upon *HSF1* overexpression determined by RNA-seq and Hsf1 bound targets determined by ChIP-seq. Hsf1 targets includes those promoters bound in either the wild-type or overexpression strain. Selected genes associated with filamentation are indicated in the diagram. f) A heat map showing gene expression changes upon *HSF1* overexpression for filamentation regulators. Colored dots are included to indicate whether the respective gene is bound by Hsf1 in the wild-type strain (in red) or only when Hsf1 is overexpressed (in orange). The colour bar depicts the change in expression as determined by RNA-seq.

As a transcription factor, Hsf1 is poised to induce filamentation via transcriptional regulation of target genes, but it is not clear whether Hsf1 would bind to and control the same or distinct sets of target genes when expressed at different levels. To determine this and the direct targets of Hsf1, we performed chromatin immunoprecipitation of TAP-tagged Hsf1 coupled with sequencing (ChIP-seq) on strains expressing basal (*HSF1-TAP/HSF1*) or overexpressed (*tetO-HSF1-TAP/tetO-HSF1)* levels of Hsf1. Model-based Analysis of ChIP-seq (MACS) and promoter analyses identified 158 promoters to which Hsf1 binds when expressed at a basal level (referred to as basal targets), including many known targets of Hsf1 such as *HSF1* itself, *HSP104*, *HSP90*, and six Hsp90 co-chaperones (*AHA1*, *CDC37*, *CPR6*, *HCH1*, *SBA1*, and *STI1*)[4] **(Fig 7A** and **S2 Data)**. Consistent with the known functions of Hsf1-regulated genes[3, 4], GO term analysis of the basal targets identified an enrichment of processes related to protein folding, protein refolding, and response to temperature stimulus **(S2 Data).** In the *HSF1* overexpression strain, Hsf1 bound to the promoters of all the basal targets, as well as an additional 1,020 targets (referred to as overexpression-specific targets) **(Fig 7A** and **S2 Data)**. Close examination of the Hsf1 ChIP-seq signal under basal conditions revealed that the majority of these overexpression-specific targets had weak but distinct binding signals **(Fig 7B)**, suggesting that these targets are also bound at a low level under basal conditions. *De novo* motif analysis by Multiple EM for Motif Elicitation (MEME) of the 200 bp sequences spanning the binding sites of the Hsf1 basal and overexpression-specific targets identified that both sets of targets had a significant enrichment for Hsf1 binding motifs following the conserved Heat Shock Element (HSE) pattern of inverted nGAAn repeats **(Fig 7C** and **S3 Data)**. The presence of these conserved Hsf1 binding motifs in the overexpression-specific targets together with the low level of Hsf1 binding to these targets under basal conditions suggests that they are *bona fide* Hsf1 targets. GO term analysis of the overexpression-specific targets showed an enrichment in genes involved in symbiosis, growth, response to stimulus, and many processes related to filamentous growth **(S2 Data)**. The overexpression-specific target set included many genes encoding morphogenetic regulators such as Ras1; the transcription factors Efg1, Ume6 and Brg1; and the hypha-specific G1 cyclin-related protein Hgc1 **(S2 Data)**. Thus, overexpressed Hsf1 engages with an expanded target set including many morphogenetic regulators, suggesting that it drives morphogenesis via overexpression of genes that promote filamentation.

To identify the Hsf1 targets that are also transcriptionally modulated upon *HSF1* overexpression, we performed genome-wide expression profiling analysis using RNA-seq. Comparison of the transcriptional profiles of the *tetO-HSF1-TAP/tetO-HSF1* strain with the wild-type strain grown in the absence of DOX revealed 1,629 genes for which expression was altered by at least 1.5-fold upon *HSF1* overexpression **(Fig 7D** and **S4 Data)**. Among the differentially expressed genes, 397 were in fact direct targets of Hsf1 **(Fig 7E)**. Intriguingly, 53% (83/158) of the basal targets had altered expression upon *HSF1* overexpression, including *HSP90*, *HSP104* and all six of the Hsf1-dependent Hsp90 co-chaperone genes **(S4 Data)**, and 31% (314/1,020) of the overexpression-specific targets had significantly altered expression. This included the upregulation of many filament-induced genes, including *ALS3*, *ECE1*, *IHD2*, *PHR1* and *RBT1*[6] which were also upregulated upon *HSF1* depletion **(S1 Data** and **S4 Data)**. It also included the induction of positive regulators of filamentation such as *HWP1*, *HGC1*, *UME6* and *BRG1*, and downregulation of the negative regulator of filamentation *NRG1* **(Fig 7E and 7F)**. Finally, the *HSF1* overexpression-specific targets encompassed genes known to be regulated by other morphogenetic regulators including the heat shock factor (HSF)-type transcription factors Sfl2 and Skn7[45, 46]. These Hsf1-dependent morphogenetic regulators are ideal candidates for targets through which *HSF1* overexpression could induce morphogenesis.

**Fig 8:**
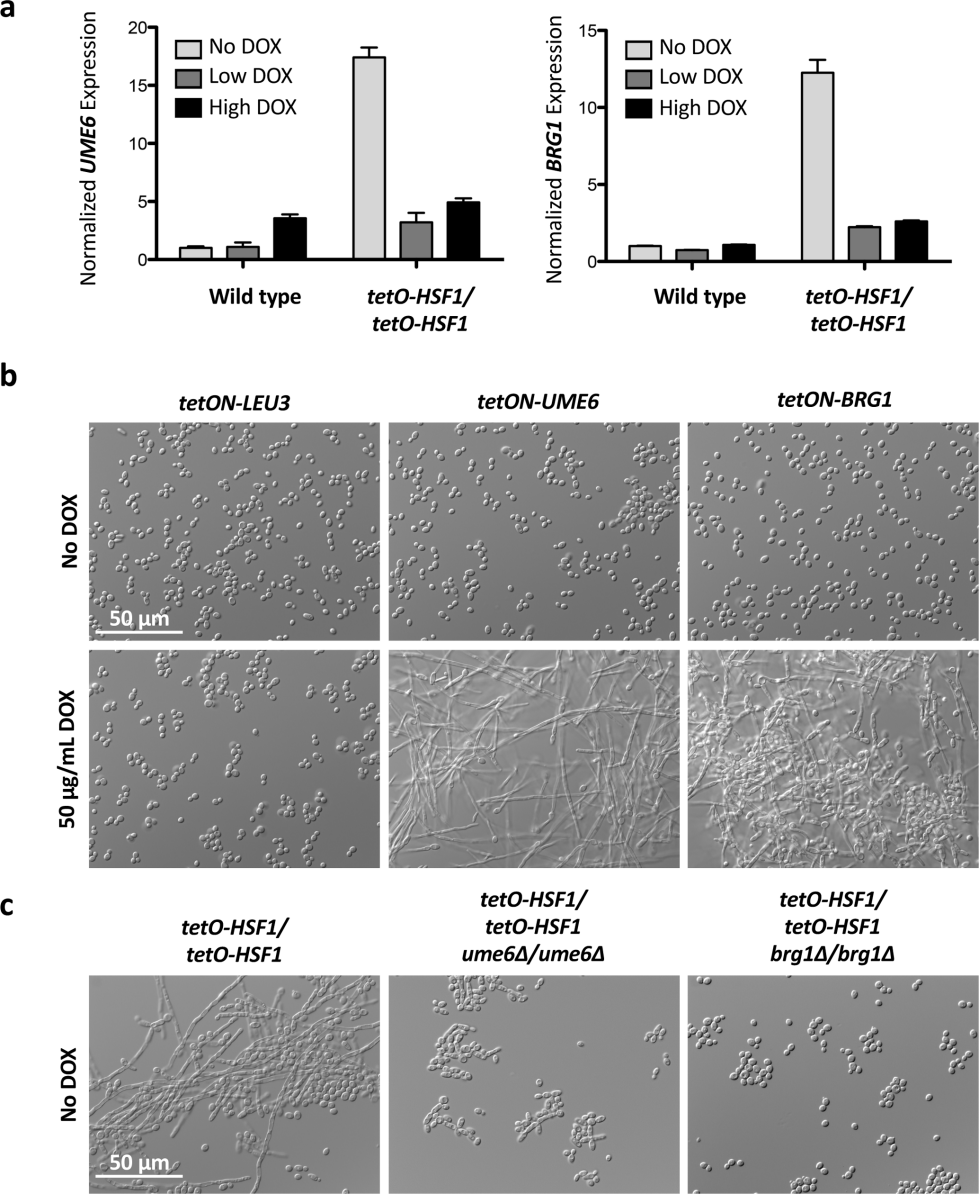
Overexpression of *HSF1* induces filamentation through overexpression of positive regulators of filamentation. a) Overexpression of *HSF1* drives the overexpression of *UME6* and *BRG1*. Strains were grown in the presence of no DOX, 0.1 μg/mL DOX (Low DOX), or 20 μg/mL DOX (High DOX) at 30°C. *UME6* and *BRG1* transcript levels were normalized to *ACT1* and *GPD1*. Data are means +/- standard error of the means for triplicate samples. b) Overexpression of *UME6* or *BRG1* induces filamentation. Strains with a tetracycline-inducible promoter (*tetON*) driving the expression of target genes were grown in the presence of 50 μg/mL DOX to induce overexpression at 30°C. Overexpression of *LEU3* acts as a negative control. c) Loss of Ume6 or Brg1 blocks filamentation induced by *HSF1* overexpression. Strains were grown in the absence of DOX at 30°C.

We focused on two well-established positive regulators of filamentation, Ume6 and Brg1, for which overexpression is sufficient to induce morphogenesis[10, 47]. We validated the RNA-seq analysis and confirmed that *HSF1* overexpression drives overexpression of *UME6* and *BRG1* **(Fig 8A)**. The overexpression of *UME6* and *BRG1* is dependent on *HSF1* levels, as the overexpression was lost when *HSF1* was depleted with DOX **(Fig 8A)**. Next, we confirmed that overexpression of *UME6* or *BRG1* was sufficient to induce filamentation using tetracycline-inducible conditional expression strains in which target genes are overexpressed in the presence of DOX[47] **(Fig 8B** and **S10A Fig)**. Overexpression of the leucine biosynthesis gene *LEU3*, a gene that does not influence the yeast-to-filament transition, was included as a control. Intriguingly, we observed that the overexpression of *UME6* and *BRG1* upon *HSF1* overexpression was lost when the *tetO-HSF1/tetO-HSF1* strain was grown at 23°C **(S10B Fig)**, a temperature at which *HSF1* overexpression is not able to induce filamentation **(Fig 2A)**. Finally, to assess the impact of Ume6 and Brg1 on filamentation induced by *HSF1* overexpression, *tetO-HSF1/tetO-HSF1* strains were generated in homozygous deletion mutants of *UME6* or *BRG1*. Loss of either Ume6 or Brg1 was sufficient to block filamentation in response to *HSF1* overexpression **(Fig 8C)**. Although Ume6 and Brg1 are necessary for filamentation in response to many cues[9, 11], they are not strictly required for polarized growth as neither Ume6 or Brg1 are required for filamentation in response to geldanamycin[20, 44], and Brg1 is dispensable for filamentation in RPM1 and Lee’s Medium[44]. Together, this is consistent with a model in which *HSF1* overexpression drives filamentation through the upregulation of positive regulators of filamentation, including *UME6* and *BRG1*.

## Discussion

**Fig 9:**
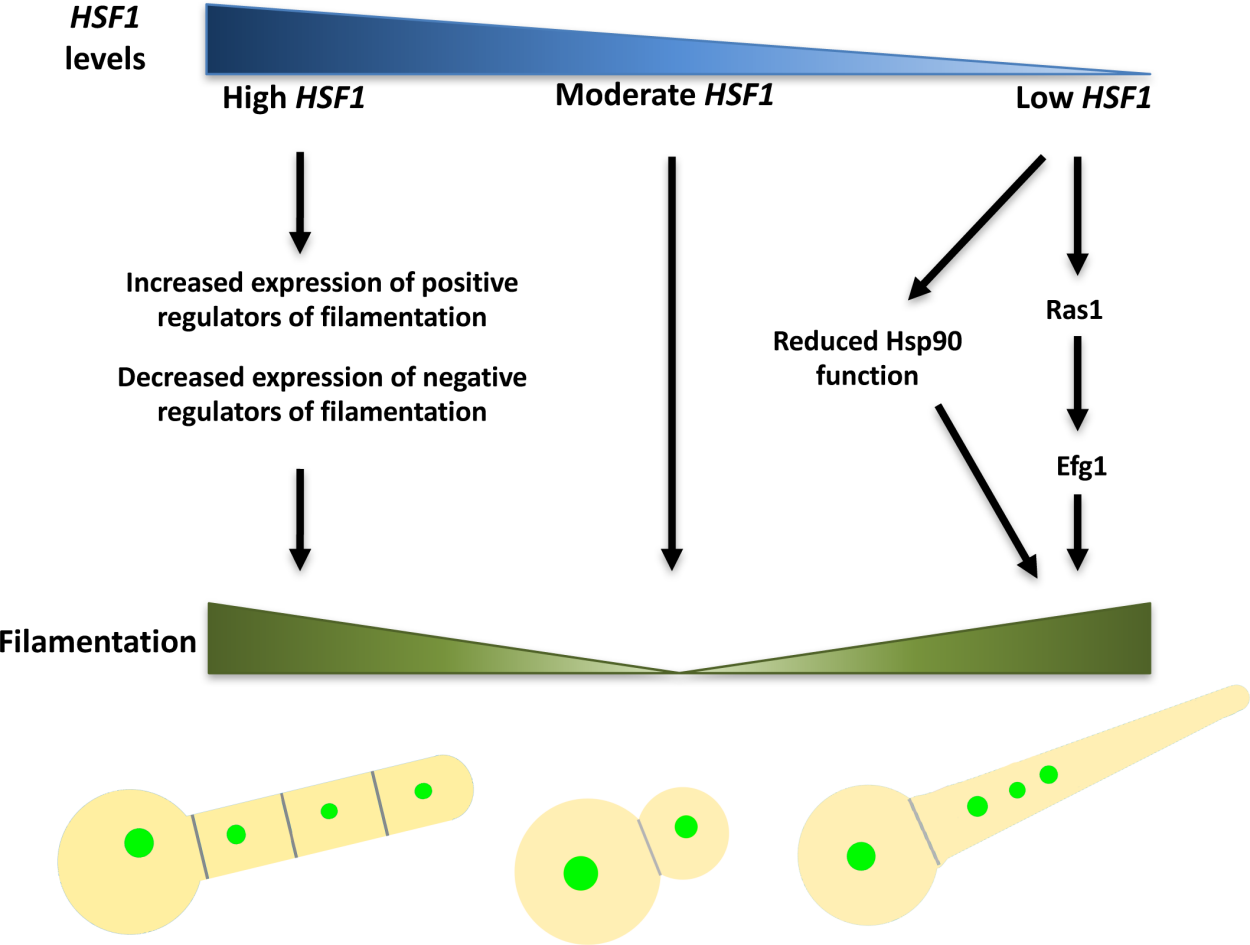
Model for how *HSF1* overexpression and depletion induce filamentation. Under basal conditions, *HSF1* levels are moderate and *C*. *albicans* exists in the yeast form. A reduction in *HSF1* levels leads to filamentous growth both by compromising Hsp90 function and through circuitry that is independent of Hsp90 but dependent on Efg1. An increase in *HSF1* levels induces a dose-dependent expansion of Hsf1 direct targets that drives overexpression of positive regulators of morphogenesis, including Brg1 and Ume6, and decreased expression of negative regulators of morphogenesis, such as Nrg1, resulting in filamentous growth. Filaments induced by *HSF1* overexpression and depletion are structurally distinct, require different genetic circuitry and are induced through distinct mechanisms.

*C*. *albicans* morphogenesis is regulated by complex genetic circuitries that enable sensing and responding to environmental inducing signals. Here, we implicate Hsf1 as a novel regulator of filamentation, expanding its function beyond its classical role in regulating the heat shock response and providing a fascinating example of how homeostasis of Hsf1 levels is required to maintain the yeast state **(Fig 9)**. Depletion of *HSF1* leads to an impairment of Hsp90 function even when *HSP90* levels are held constant and independent of Hsf1 **(Fig 3** and **Fig 4)**, which provides a mechanism through which *HSF1* depletion could induce filamentous growth. *HSF1* depletion also induces filamentation through mechanisms distinct from Hsp90, which require the cAMP-PKA pathway and Efg1 **(Fig 5)**. Overexpression of *HSF1* also induces filamentous growth, but does so by means of engaging with an expanded set of targets, thereby up-regulating the expression of positive regulators of filamentation such as Ume6 and Brg1, and down-regulating the expression of negative regulators of filamentation such as Nrg1 **(Fig 7** and **Fig 8)**. As a central transcriptional regulator that is exquisitely responsive to environmental cues, Hsf1 serves as a master regulator of *C*. *albicans* morphogenesis.

We establish that in addition to Hsf1’s role in transcriptional regulation of *HSP90*, Hsf1 also influences Hsp90 function **(Fig 4)**, providing an additional layer of complexity to the known regulatory feedback loop between Hsf1 and Hsp90 in which Hsf1 drives *HSP90* expression and Hsp90 represses Hsf1 activity[15]. Hsp90 activity is under intensive regulation by co-chaperones and enzymes that post-translationally modify Hsp90[15], and we postulate that Hsf1 could alter Hsp90 function by controlling the expression of these critical regulators. Our findings that loss of any single Hsf1-dependent Hsp90 co-chaperone is insufficient to induce filamentation **(S9 Fig)**, suggests that misregulation of multiple Hsp90 regulatory proteins, including co-chaperones, lysine deacetylases or kinases in concert could be necessary to compromise Hsp90 function to induce filamentation. It is also possible *HSF1* depletion modulates Hsp90 function through a mechanism independent of its transcriptional activity, by altering complex formation between Hsp90 and its clients or regulators. This is consistent with studies in mammalian cells, which demonstrated that *HSF1* depletion impairs Hsp90 function in chaperoning kinase client proteins through reduced associations between Hsp90, Cdc37, and kinase clients[48, 49], as well as the reduced association between Hsp90 and the deacetylase HDAC6[49]. It is also possible that *HSF1* depletion affects Hsp90 function indirectly. In *S*. *cerevisiae* it has been shown that loss of Hsf1 function leads to an accumulation of misfolded proteins due to the downregulation of Hsp90 and Hsp70 [41]. Since Hsf1 is a key regulator of many proteostasis genes, we hypothesize that *HSF1* depletion would also lead to an accumulation of misfolded proteins, which would exceed the functional capacity of Hsp90. These possibilities are not mutually exclusive, and *HSF1* depletion may induce filamentation through multiple of these mechanisms, as well as through an Hsp90-independent mechanism that requires the terminal transcription factor of the cAMP-PKA pathway, Efg1.

In contrast, *HSF1* overexpression induces filamentation independent of Hsp90 **(Fig 6)**, but in an exquisitely temperature-dependent manner **(Fig 1** and **Fig 2)**. Our transcriptional analyses revealed that upon overexpression, Hsf1 engages with an expanded target set, thereby directly activating a filamentation transcriptional program by up-regulating positive regulators of filamentation including *HGC1*, *HWP1*, *UME6* and *BRG1*, and down-regulating repressors such as *NRG1* **(Fig 7)**. We previously established that ~80% of *C*. *albicans* promoters contain at least one Hsf1 consensus sequence[4], although Hsf1 was not bound to most of these promoters under basal or heat shock conditions, suggesting that there might be extensive environmentally contingent Hsf1 binding. Increased levels of Hsf1 protein due to *HSF1* overexpression or elevated temperature could allow Hsf1 to bind to additional targets. Even when overproduced, the transcriptional output of Hsf1 is modulated by the environment, as filamentation in response to *HSF1* overexpression was abolished at 23°C as was up-regulation of target genes involved in filamentation **(Fig 2A** and **S10B Fig)**. This suggests that the transcriptional activity of Hsf1 is affected by additional factors that are dependent on environmental temperatures and might include nucleosome occupancy or post-translational modifications. Nucleosome occupancy at stress responsive genes is modulated by Hsp90[4], and nucleosome positioning may also be modulated by temperature, thereby altering accessibility of Hsf1 consensus sequences. Moreover, Hsf1 activity is regulated by phosphorylation, which increases upon heat shock[3, 25], and the phosphorylated form of *S*. *cerevisiae* Hsf1 recruits the Mediator complex to the promoters of its target genes, mediating transcription[31]. These results suggest a model in which Hsf1 may be bound to its target promoters in an unphosphorylated state at lower temperatures and therefore be unable to recruit the Mediator to influence gene expression. Taken together, our findings illuminate a new facet to repertoire of mechanisms that microbial pathogens use to couple sensing the environment to activation of a developmental program important for virulence.

By providing the first example of a protein that acts both as a positive and negative regulator of filamentation, this work challenges the classical model of morphogenetic regulation in which overexpression and depletion of key regulators have opposing effects on filamentation. As a classic example, Ume6 is a positive regulator of filamentation that promotes polarized growth in a dose-dependent manner[10, 11]. In contrast, our studies demonstrate that homeostasis of *HSF1* levels is required to maintain the yeast state, and either overexpression or depletion of *HSF1* induces filamentation. The filaments induced by overexpression of *HSF1* differ considerably from those induced by depletion of *HSF1* in terms of both morphology, temperature dependence, and mechanisms involved. Given that *HSF1* expression and activity are exquisitely sensitive to the environment **(Fig 1B, Fig 2A** and **S4 Fig)**, it is poised to sense and transduce environmental signals to orchestrate morphogenetic programs.

This work highlights Hsf1 as an environmentally contingent regulator for which tuning levels has a profound impact on diverse aspects of cellular biology, including response to temperature, morphogenesis, proteostasis, cell cycle, host cell adhesion and damage[4], drug sensitivity[50] and iron homeostasis[51]. Beyond these processes, Hsf1 may also influence additional *C*. *albicans* phenotypic states such as the white, grey, opaque and gastrointestinally induced transition (GUT) cell morphotypes, which are intimately connected with mating and host interactions[52]. Consistent with this expectation, *HSF1* levels vary within cells of these states[53–55], and the master regulators of these transitions, Wor1 and Efg1[52], are targets of Hsf1 upon overexpression **(S2 Data)**. We also observed that *HSF1* levels were dramatically increased in biofilm growth conditions **(S4B Fig)**, suggesting that cellular morphologies in this host-relevant state may be influenced by elevated *HSF1* levels **(S4 Fig)**. Hsf1 may also have a much broader role in governing virulence traits of diverse fungi such as thermally dimorphic fungi or *C*. *neoformans* for which *HSF1* overexpression enables thermotolerance[27]. With Hsf1 and Hsp90 as core hubs of cellular circuitry in diverse cells types, there has been a growing appreciation of the therapeutic potential of targeting these regulators in treating cancer[56, 57], neurodegenerative disorders[58], and infectious disease[21]. Realizing this potential will require the development of novel chemical matter that can selectively perturb proteostasis and exploit differences between mammals and fungi in structures and functional relationships of Hsf1 and Hsp90. Exploring this core cellular circuitry creates unprecedented opportunity for understanding mechanisms governing environmentally responsive developmental programs and for developing new therapeutic strategies for devastating diseases.

## Materials and methods

### Strains and culture conditions

All strains used in this study are listed in **S1 Table**. All plasmids used to create strains are included in **S2 Table**. All oligonucleotide sequences used in this study are included in **S3 Table**. Strains were grown in rich YPD medium (1% yeast extract, 2% bactopeptone, 2% glucose) or YPM medium (1% yeast extract, 2% bactopeptone, 2% maltose) at 30°C, unless indicated otherwise. Archives of C. albicans strains were maintained at -80°C in YPD with 25% glycerol. For solid medium, 2% agar was used. Strains that were auxotrophic for uridine were grown with 80 mg/L uridine added to the growth medium. When indicated, strains were grown in the presence of doxycycline (Doxycycline Hydrochloride, BioBasic, DB0889) dissolved in water.

Since the *tetO-HSF1/hsf1Δ* and *tetO-HSF1/tetO-HSF1* strains have different levels of *HSF1* overexpression when grown in the absence of DOX **(Fig 1A)**, the strains were grown for different lengths of time to deplete *HSF1* and observe the relevant phenotypes. For experiments with *tetO-HSF1/hsf1Δ* strains looking at *HSF1* depletion, unless indicated otherwise, overnights of relevant strains and controls were subcultured in the absence or presence of 20 μg/mL DOX and grown overnight. The cells were subcultured into the same conditions and grown to mid-log phase for quantitative reverse transcription PCR (qRT-PCR) experiments and western blot analysis or were grown overnight for microscopy analysis. For experiments with *tetO-HSF1/tetO-HSF1* strains looking at *HSF1* depletion, unless indicated otherwise, overnights of relevant strains and controls were subcultured in the absence or presence of DOX (at the indicated concentrations) and grown overnight. The cells were subcultured into the same conditions and grown for an additional overnight. Then, cells were subcultured into the same conditions and grown to mid-log phase for qRT-PCR experiments and western blot analysis or were grown overnight for microscopy analysis. For experiments monitoring *HSF1* overexpression only, unless indicated otherwise, overnights were subcultured and grown in the absence of DOX to mid-log phase for qRT-PCR experiments and western blot analysis or were grown overnight for microscopy analysis.

To overexpress *LEU3*, *UME6* or *BRG1* using the *tetON* strains, overnights were subcultured in the absence or presence of 50 μg/mL DOX and grown overnight. The cells were subcultured into the same conditions for an additional overnight to achieve sufficient overexpression of *UME6* and *BRG1* to induce filamentation, allowing for microscopy analysis. For qRT-PCR experiments, cells were subcultured into the same conditions and grown to mid-log phase.

In order to monitor *HSF1* levels in biofilm growth conditions, biofilms were grown in multi-well six well plates (Falcon). An overnight culture of the wild-type strain (*HSF1-TAP/HSF1*) was grown at 30°C in rich medium and diluted to an optical density at 600 nm of 0.5 in Spider medium (1% nutrient broth, 1% mannitol, 0.2% K_2_HPO_4_). The suspension was inoculated into sterile, six-well plates that had been preincubated with bovine serum (Gibco Life Technologies, 16170078) for 90 minutes and washed once with PBS. Cells were incubated for 90 minutes at 37°C in static conditions. Non-adherent cells were washed away once with PBS and fresh Spider medium was added. Plates were incubated for 48 hours at 37°C in static conditions. The supernatant was removed and the wells were washed once with PBS before the biofilm cells were collected by scraping. The pelleted cells were washed with cold, distilled water before being flash-frozen on a dry ice ethanol bath.

In order to monitor *HSF1* levels upon heat shock, an overnight culture of the wild-type strain (*HSF1-TAP/HSF1*) was grown at 30°C in rich medium, diluted to an optical density at 600 nm of 0.1 and grown at 30°C until mid-log phase. The cells underwent a 30°C to 42°C heat shock, by diluting the mid-log phase cells two-fold in medium prewarmed at 54°C and incubating the cells at 42°C for 10 minutes before pelleting. The pelleted cells were washed with cold, distilled water before being flash-frozen on a dry ice ethanol bath.

### Quantitative reverse transcription-PCR (qRT-PCR)

To monitor gene expression changes, strains were grown to mid-log phase, pelleted at 3000 rpm at 4°C and washed with cold, distilled water before being flash-frozen on a dry ice ethanol bath. The pellets were stored at -80°C. Cells were lysed by bead beating, six times for 30 seconds each, with one minute on ice between. RNA was extracted from the lysed cells using the QIAGEN RNeasy kit and DNase treated using the QIAGEN RNase free DNAase Set. Complementary DNA synthesis was performed using the AffinityScript Multi Temperature cDNA Synthesis Kit (Agilent Technologies). qRT-PCR was performed using in a 384-well plate, with a 10 μL reaction volume using Fast SYBR Green Master Mix (Applied Biosystems) and the BioRad CFX-384 Real Time System with the following cycling conditions: 95°C for 3 minutes, then 95°C for 10 seconds and 60°C for 30 seconds, for 40 cycles. The melt curve was completed with the following cycle conditions: 95°C for 10 seconds and 65°C for 10 seconds with an increase of 0.5°C per cycle up to 95°C. Reactions were performed in technical triplicate using the primer pairs oLC2285/oLC2286 for *ACT1*, oLC752/oLC753 for *GPD1*, oLC6472/oLC6473 for *PMA1*, oLC2451/oLC2452 for *HSF1*, oLC756/oLC757 for *HSP90*, oLC1460/oLC1461 for *UME6*, and oLC2635/oLC2636 for *BRG1*. Primer sequences are included in **S3 Table**. Data were analyzed using the BioRad CFX Manager 3.1. Error bars depict standard error of the means of technical triplicates.

### Microscopy

Cells cultured in liquid medium were imaged using differential interference contrast (DIC) microscopy with a Zeiss Axio Imager.MI (Carl Zeiss) and an X-cite series 120 light source for fluorescence. ET green fluorescent protein (GFP), 4’,6-diamidino-2-phenylindole (DAPI) hybrid, and ET HQ tetramethylrhodamine isothiocyanate (TRITC)/DsRED filter sets from Chroma Technology (Bellows Falls, VT) were used to visualize Nop1-GFP, calcofluor white, and propidium iodide respectively. Propidium iodide (Sigma-Aldrich P4170, dissolved in water) was added to cells at a final concentration of 25 μg/mL and incubated in the dark for 5 minutes before imaging. Calcofluor white (Fluorescent Brightener 28, Sigma-Aldrich F3543, dissolved in water) was added to cells at a final concentration of 5 μg/mL. Quantification of nuclei was performed by counting the number of nuclei per cellular compartment in at least 300 compartments per condition for two biological replicates, using Image J software. Statistical significance was determined using an unpaired t test using GraphPad software (https://www.graphpad.com/quickcalcs/ttest1.cfm).

### Growth curves

Sensitivity to geldanamycin was determined using minimum inhibitory concentration assays in 96-well microtiter plates (Sarstedt) as previously described[59–61]. Assays were performed in YPD with a total volume of 200 μL/well with two-fold dilutions of geldanamycin (LC Laboratories, G-4500, dissolved in DMSO). Strains were grown in the absence or presence of 20 μg/mL doxycycline dissolved in water. Strains were grown in a TECAN GENios plate reader at 30°C, with orbital shaking on high. Absorbance readings were measured using the XFluor4 software at an optical density of 595 nm every 15 minutes for 24 hours. Data is displayed for the growth in 3.13 μM geldanamycin, where there was minimal growth inhibition of the wild-type strain. To monitor filamentation in response to geldanamycin, minimum inhibitory concentration assays were performed as above but incubated at 30°C in static conditions. Filamentation was monitored at the highest concentration of geldanamycin that did not cause substantial growth inhibition upon *HSF1* depletion.

### Microarray analysis

Three independent overnights of the *tetO-HSF1/hsf1Δ* strain were grown in YPD and subcultured to an optical density of 600 nm (OD_600_) of 0.2 in the absence or presence of 20 μg/mL DOX overnight. The cells were subcultured into the same conditions for an additional overnight to achieve sufficient *HSF1* depletion to induce filamentation. Finally, the cells were subcultured to OD_600_ of 0.05 into the same conditions in the absence or presence of DOX and grown to mid-log phase. At this time point, the *HSF1* depleted cells were robustly filamentous. Cells were spun down at 4000 rpm for 5 minutes at 4°C, washed once with cold water, and spun again. All liquid was removed and the pellet was frozen in liquid nitrogen before storage at -80°C. RNA was extracted from the pellets by bead beating for 30 seconds, six times, with one minute on ice between. Pellets were split into multiple bead beating tubes for a higher efficiency of breaking open the cell. RNA was extracted using the Qiagen RNeasy Mini Kit. RNA purity and integrity was assessed by nanodrop and electrophoresis on a 1% agarose gel. Microarray analysis was performed as previously[20, 62]. Briefly, RNA was reverse transcribed using Superscript III Reverse Transcriptase (Invitrogen) and oligo(dT)21 in the presence of Cy3- or Cy5-dCTP (Invitrogen). The samples were treated with 2.5 units RNase H (USB) and 1 μg RNase A (Pharmacia) at 37°C for 15 minute to degrade template RNA. Labeled cDNA was purified with a QIAquick PCR Purification Kit (QIAGEN). Hybridization was performed using DIG Easy Hyb Solution (Roche Diagnostics) containing 0.45% salmon sperm DNA and 0.45% yeast tRNA at 42°C for 24 hours in a SlideBooster Hyb chamber SB 800 (Advalytix, Brunnthal, Germany) with regular microagitation. The slides were washed once in 1.0% SSC (0.15 M NaCl and 0.015 M sodium citrate) with 0.2% SDS at 42°C for 5 minutes and twice in 0.1% SSC with 0.2% SDS at 42°C for 5 minutes. The slides were additionally washed once in 0.1% SSC at 24°C for 5 minutes, followed by four rinses in 0.1% SSC. Microarray slides were air dried before being scanned using a ScanArray Lite microarray scanner (Perkin Elmer-Cetus; versions 2.0 and 3.0). The microarray data was analyzed with GeneSpring GX v7.3 (Agilent Technologies). Raw intensities were normalized with a Lowess curve using 20% of the data to fit each point. To identify transcripts with a significant change in abundance, the fluorescence ratios were compared using the Welch t-test and the Benjamini and Hochberg False Discovery Rate. Volcano plots were used to identify genes with statistically significant (P<0.5) changes in transcript abundance of greater than 1.5 fold. Gene Set Enrichment Analysis was performed as described previously[63], with details provided at http://www.broadinstitute.org/gsea/.

### ChIP-seq and RNA-seq

Four independent overnights of the wild-type (CaLC2302), *HSF1-TAP/HSF1* (CaLC5012), and *tetO-HSF1-TAP/tetO-HSF1* (CaLC5014) strains were subcultured to an OD_600_ of 0.1 and grown to mid-log phase, approximately 4 hours. The culture was split so that ~45mL was spun down for RNA-seq and ~45mL was prepared for ChIP-seq. The samples for ChIP-seq were prepared as described previously[4, 64]. Briefly, 40 mL of culture was combined with 1.2 mL of 37% formaldehyde and incubated with gentle rocking for 20 minutes at room temperature. 10 mL of 2.5M glycine was then added to stop the crosslinking and cells were incubated with gentle rocking for 10 minutes. Cells were harvested by spinning at 3000 rpm for 2 minutes at 4°C, washing twice in 20 mL ice-cold Tris buffered saline (TBS). Pellets were frozen in liquid nitrogen and kept at -80°C until chromatin preparation. Cell pellets were lysed in ice-cold FA lysis buffer in a Bullet Blender for three times five minutes at setting 12. Chromatin was pelleted, resuspended in fresh FA lysis buffer and fragmented in a Qsonica Q800 sonicator with sonication cycles of 10 seconds on and 10 seconds off for a total sonication time of 20 minutes. Immunoprecipitation was carried out using ~15 μl IgG Sepharose (GE Healthcare) for Hsf1-TAP on an end-to-end rotator for at least 3 hours at room temperature before washing with 2 x FA lysis buffer, 1 x FA lysis buffer with 500 mM NaCl, 1 x LiCl buffer and 1 x TE buffer, as described previously[65]. Library preparation of ChIP DNA samples were carried out as described previously[66]. Single end 60 bp sequencing was performed on the Illumina 2500 platform. The samples for RNA-seq were prepared as described previously[4]. Briefly, cells were pelleted at 3000 rpm for 5 minutes at 4°C, washed with ice cold TBS, at which point supernatant was removed and the pellets were frozen in liquid nitrogen. All pellets were stored at -80°C. RNA was extracted using the Qiagen RNeasy Mini Kit and DNase treated using the Ambion DNA free kit. Quality was assessed by monitoring the 260/280 and 260/230 ratios and using Agilent RNA Bioanalyzer assay. When necessary, the RNA was cleaned up using the Qiagen RNeasy MinElute Cleanup column kit. Library preparation was carried out using TruSeq Stranded mRNA Sample Prep kit and TruSeq DNA Sample Prep PCR kit according to manufacturer’s instruction protocol version revision E. Pair-end 150 sequencing was done by the Illumina technology.

### ChIP-seq data analysis

De-multiplexing and processing of ChIP-seq data was performed according to previous studies[66, 67]. Barcodes were removed from the 5’ end of each reads and mapped to the *C*. *albicans* A21 reference genome sequences using Bowtie2 with default parameters. Hsf1 binding peaks were identified by Model-based Analysis of ChIP-seq (MACS)[68] using standard parameters with -log_10_(q-value) cutoff of 50. *De novo* motif discovery was performed using MEME-ChIP on 200 bp sequences spanning Hsf1 summits reported by MACS (i.e. 100 bp on each side of summits)[69]. Hsf1 target genes were identified by mapping genes with ATG closest to Hsf1 binding sites using an in-house R-script. To identify Hsf1 motifs at promoters, canonical Hsf1 binding sites as well as motifs reported by the MEME analysis were searched and counted within 1 kb promoter sequence of Hsf1 target genes using an in-house R-script. ChIP-seq data are available at SRA under the accession number SRP119587.

### RNA-seq data analysis

Raw data was mapped to the *C*. *albicans* reference genome (A21) using Tophat2[70] and gene expression values (fragments per kilobase of exon per million fragments mapped–FPKM) were calculated using Cuffdiff2[71] and genes with expression values larger than 1.5 are classified as differential expression genes. RNA-seq data are available at SRA under the accession number SRP119587.

### Western blot analysis

Proteins were extracted for western blot analysis by two different methods. For western blots monitoring *HSF1* overexpression alone, samples were prepared using a quick, protein extraction method. Briefly, cells were grown to mid-log phase and the equivalent of 1 mL of each culture at an OD_600_ of 1 was pelleted, spinning for 15 seconds on high. The supernatant was removed and the cells were spun for 15 seconds again, to remove all remaining liquid. The pellet was resuspended in 60 μL of 1X sample buffer (one-sixth volume of 6x sample buffer containing 0.35 M Tris-HCl, 10% (w/w) SDS, 36% glycerol, 5% β-mercaptoethanol, and 0.012% bromophenol blue). Samples were boiled for 5 minutes at 100°C. Cell debris was pelleted by spinning for 5 minutes at 14,000 rpm. Supernatant was loaded onto an 8% SDS-PAGE gel. For western blots monitoring *HSF1* depletion, cells were grown overnight, subcultured to an OD_600_ of 0.1 in the absence or presence of the indicated concentrations of DOX and grown again. Cells were subcultured to an OD_600_ of 0.1 in the same conditions and grown until mid-log phase. Cells were pelleted at 3000 rpm at 4°C and washed with cold, distilled water before being flash-frozen on a dry ice ethanol bath. The pellets were stored at -80°C. For western blot analysis to detect pHog1, cells were treated for 10 minutes with 5 mM hydrogen peroxide (Sigma-Aldrich) before pelleting. Cells were lysed by bead beating, six times for 30 seconds each, with one minute on ice between. For all westerns, separated proteins were electrotransferred to polyvinylidene difluoride (PVDF) membrane (Bio-Rad Laboratories, Inc.). Blots were blocked with 5% skim milk in phosphate-buffered saline with 0.2% Tween 20 (PBS-T), except for pHog1 blots which were blocked in 5% Bovine Serum Albumin in TBS with 0.2% Tween 20 (TBS-T). Blots were hybridized with primary antibodies against the TAP epitope (1:3000, Thermo Fisher Scientific, CAB1001), pHog1 (1:1000, Phospho-p38 MAPK from Cell Signaling, 92155), Hog1 (1:1000–1:2500, Santa Cruz Biotechnology, SC 9079), Hsp90 (1:10,000, Bryan Larson), β-Actin (1:5000, Santa Cruz Biotechnology, SC 47778) or Tubulin (1:1000–1:5000, AbDseroTec, MCA78G) in block solution. Blots were washed with PBS-T or TBS-T and incubated with FITC-conjugated secondary antibodies diluted 1:5000 in the block solution. Blots were washed with PBS-T or TBS-T before detecting the signals using an ECL western blotting kit as per the manufacturer’s instructions (Pierce).

### Strain construction

Strains were constructed according to standard protocols. To select for mutants prototrophic for arginine or histidine, synthetic defined medium plates (0.17% yeast nitrogen base without ammonium sulfate, 0.1% glutatmic acid, 2% glucose, 2% agar) were supplemented with arginine HCl (50 mg/L) or histidine HCl (20mg/L) as required. To select for nourseothricin (NAT)- resistant mutants, nourseothricin (Jena Bioscience) was solubilized in water and supplemented into YPD plates at a final concentration of 150 μg/mL.

**CaLC970:** To construct the *HSF1/hsf1Δ* strain, the plasmid pLC478, which contains the *HSF1* knockout construct, was digested with BssHII and transformed into CaLC239 (SN95). NAT-resistant transformants were PCR tested for proper integration of the construct using primer pairs oLC989/oLC275 and oLC274/oLC990. The *SAP2* promoter was induced to drive expression of FLP recombinase to excise the NAT marker cassette.

**CaLC2928:** To construct the *tetO-HSF1/hsf1Δ* strain, the plasmid pLC551 was digested with SapI and KpnI to liberate the *TAR-tetO-HSF1* construct. This construct was transformed into CaLC970 (*HSF1/hsf1Δ)*. NAT-resistant transformants were PCR tested for proper integration of the cassette using primer pairs oLC274/oLC996 and oLC275/oLC995. The *SAP2* promoter was induced to drive expression of FLP recombinase to excise the NAT marker cassette. NAT-sensitive colonies were additionally PCR tested with the primer pairs oLC985/oLC988 to verify the presence of the deleted allele of *HSF1*, oLC991/oLC994 to ensure there were no remaining wild-type alleles of *HSF1* present, and oLC300/oLC994 to verify the presence of the *tetO* promoter.

**CaLC3017:** To construct the *tetO-HSF1/hsf1Δ ACT1p-HSP90/ACT1p-HSP90* strain the *ACT1p-HSP90* construct with homology to the *PHO23* locus was liberated by digesting the plasmid pLC755 with BssHII. The digested plasmid was transformed into CaLC2928 (*tetO-HSF1/hsf1Δ)*. NAT-resistant transformants were PCR tested with the primer pairs oLC275/oLC376 and oLC381/oLC324 to verify the integration of the *ACT1p-HSP90* cassette at the *PHO23* locus. The *SAP2* promoter was induced to drive expression of FLP recombinase to excise the NAT marker cassette. NAT-sensitive colonies were additionally PCR tested with the primers oLC1482/oLC609 to verify the presence of the *ACT1p-HSP90*. To put a second copy of *HSP90* under the control of *ACT1p* in the genome, an *ACT1p-HSP90* cassette with homology to the *ACT1* locus was liberated by digesting the plasmid pLC757 with BsrGI. The digested plasmid was transformed into the strain. NAT-resistant transformants were PCR tested with the primers oLC3022/oLC199 to verify integration of the *ACT1p-HSP90* cassette into the *ACT1* locus.

**CaLC3384 and CaLC3385:** To construct *tetO-HSP90/hsp90Δ* strains, the plasmid pLC62 containing the *HSP90* knockout construct was digested with KpnI-HF and SacII and transformed into CaLC239 (SN95). NAT-resistant transformants were PCR tested for proper integration using the primer pairs oLC275/oLC276 and oLC277/oLC274. The *SAP2* promoter was induced to drive expression of FLP recombinase to excise the NAT marker cassette. The *tetO* promoter replacement construct was PCR amplified from pLC605 using the primers oLC3390 and oLC3220 and transformed into the heterozygous mutant strain. NAT-resistant transformants were PCR tested for proper integration of the *tetO* promoter at the *HSP90* locus using the primers oLC294/oLC534 and oLC300/oLC408. Transformants were PCR tested for the absence of a wild-type promoter of *HSP90* using the primers oLC294/oLC297. The *SAP2* promoter was then induced to drive expression of FLP recombinase to excise the NAT marker cassette.

**CaLC2995:** To construct the *tetO-HSF1-TAP/hsf1Δ* strain, the *TAP-ARG4* cassette was PCR amplified from pLC573 using the primers oLC2950 and oLC2922 and transformed into CaLC239 (SN95). *ARG4+* transformants were PCR tested for correct integration of TAP at the C-terminus of *HSF1* using the primer pairs oLC1597/oLC1593 and oLC1598/oLC1594.

**CaLC3890:** The *tetO-HSF1-TAP/hsf1Δ ACT1p-HSP90/ACT1p-HSP90* strain was constructed using the same method as CaLC2995, except that the *TAP-ARG4* cassette was transformed into CaLC3017 (*tetO-HSF1/hsf1Δ ACT1p-HSP90/ACT1p-HSP90*).

**CaLC3786:** The *tetO-HSP90/hsp90Δ HSF1-TAP/HSF1* strain was constructed using the same method as CaLC2995, except that the *TAP-ARG4* cassette was transformed into CaLC3384 (*tetO-HSP90/hsp90Δ*).

**CaLC5012:** The *HSF1-TAP/HSF1* strain in the SN250 background was constructed using the same method as CaLC2995, except that the *TAP-ARG4* cassette was transformed into CaLC2302 (SN250). **CaLC974:** To construct the *MAL2p-HSF1/hsf1Δ* strain, the plasmid pLC481, which contains the *MAL2* promoter and sequence homologous to *HSF1*, was digested with BssHII and transformed into CaLC970 (*HSF1/hsf1Δ*). NAT-resistant transformants were PCR tested for proper integration of the construct using primer pairs oLC995/oLC275 and oLC274/oLC996. Transformants were additionally PCR tested with the primers oLC985/oLC988 to verify the presence of the deleted allele of *HSF1* and oLC991/oLC994 to ensure there were no additional wild-type alleles of *HSF1*. The *SAP2* promoter was induced to drive expression of FLP recombinase to excise the NAT marker cassette.

**CaLC3171:** To construct the *cpr6Δ/cpr6Δ* strain, the NAT knockout cassette was PCR amplified from pLC49 using primers oLC3062 and oLC3063 and transformed into CaLC239 (SN95). NAT-resistant transformants were PCR tested for proper integration using the primer pairs oLC274/oLC3065 and oLC3064/oLC275. The *SAP2* promoter was induced to drive expression of FLP recombinase to excise the NAT marker. To delete the second allele of *CPR6*, the NAT knockout cassette was again amplified from pLC49 using oLC3062 and oLC3063 and transformed into the NAT-sensitive heterozygous strain. NAT-resistant transformants were PCR tested for proper integration using the same primers as above, and with the primers oLC3064/oLC3065 to ensure no wild-type alleles of *CPR6* remained. The *SAP2* promoter was then induced to drive expression of FLP recombinase to excise the NAT marker cassette.

**CaLC3172:** To construct the *aha1Δ/aha1Δ* strain, the NAT knockout cassette was PCR amplified from pLC49 using primers oLC3058 and oLC3059 and transformed into CaLC239 (SN95). NAT-resistant transformants were PCR tested for proper integration using the primer pairs oLC274/oLC2678 and oLC3172/oLC275. The *SAP2* promoter was induced to drive expression of FLP recombinase to excise the NAT marker. To delete the second allele of *AHA1*, the NAT knockout cassette was again amplified from pLC49 using oLC3058 and oLC3059 and transformed into the NAT-sensitive heterozygous strain. NAT-resistant transformants were PCR tested for integration using the same primers as above, and with the primers oLC2677/oLC2678 to ensure no wild-type alleles of *AHA1* remained. The *SAP2* promoter was then induced to drive expression of FLP recombinase to excise the NAT marker cassette.

**CaLC3193:** To construct the *hch1Δ/hch1Δ* strain, the NAT knockout cassette was PCR amplified from pLC49 using primers oLC3086 and oLC3087 and transformed into CaLC239 (SN95). NAT-resistant transformants were PCR tested for proper integration using the primers oLC274/oLC3089 and oLC3088/3089 to ensure the presence of a deleted allele. The *SAP2* promoter was induced to drive expression of FLP recombinase to excise the NAT marker. To delete the second allele of *HCH1*, the NAT knockout cassette was again PCR amplified from pLC49 using the primers oLC3086 and oLC3087, and transformed into the NAT-sensitive heterozygous strain. NAT-resistant transformants were PCR tested for integration using the primers oLC274/oLC3089 to test for proper integration and oLC3088/oLC3089 to ensure no wild-type alleles of *HCH1* remained. The *SAP2* promoter was then induced to drive expression of FLP recombinase to excise the NAT marker cassette.

**CaLC3310:** To construct the *sti1Δ/sti1Δ* strain, the NAT knockout cassette was PCR amplified from pLC49 using the primers oLC3074 and oLC3075 and transformed into CaLC239 (SN95). NAT-resistant transformants were tested for proper integration using the primer pairs oLC274/oLC2704 and oLC275/oLC3076. The *SAP2* promoter was induced to drive expression of FLP recombinase to excise the NAT marker. To delete the second allele of *STI1*, the NAT knockout cassette was again PCR amplified from pLC49 using oLC3074 and oLC3075, and transformed into the NAT-sensitive heterozygous strain. NAT-resistant transformants were PCR tested for integration using the same primers as above, and with the primers oLC2703/oLC2704 to ensure no wild-type alleles of *STI1* remained. The *SAP2* promoter was then induced to drive expression of FLP recombinase to excise the NAT marker cassette.

**CaLC3797:** To construct the *sba1Δ/sba1Δ* strain, the NAT knockout cassette was PCR amplified from pLC49 using primers oLC3748 and oLC3067 and transformed into CaLC239 (SN95). NAT-resistant transformants were PCR tested for proper integration using the primer pairs oLC3170/oLC275 and oLC274/oLC3747. The *SAP2* promoter was then induced to drive expression of FLP recombinase to excise the NAT marker cassette. To delete the second allele of *SBA1*, the NAT knockout cassette was again PCR amplified from pLC49 using oLC3748 and oLC3067, and transformed into the NAT-sensitive heterozygous strain. NAT-resistant transformants were PCR tested for integration using the same primers as above and the primers oLC3170 and oLC3747 to ensure that no wild-type alleles of *SBA1* remained. The *SAP2* promoter was then induced to drive expression of FLP recombinase to excise the NAT marker cassette.

**CaLC3991:** To construct the *tetO-CDC37/cdc37Δ* strain, the plasmid pLC506 was digested with BssHII to liberate the *MAL2p*-*CDC37* cassette and the digested plasmid was transformed into CaLC239 (SN95). The NAT-resistant transformants were PCR tested for proper integration using the primer pairs oLC1093/oLC275 and oLC274/oLC1097. The *SAP2* promoter was then induced to drive expression of FLP recombinase to excise the NAT marker cassette. The plasmid pLC505 was digested with BssHII to liberate the *CDC37* knockout cassette and the digested plasmid was transformed into the NAT-sensitive *MAL2p-CDC37/CDC37* strain. The NAT-resistant transformants were PCR tested for proper integration using the primers oLC275/oLC1093 and oLC274/oLC1094. Transformants were additionally PCR tested with oLC441/oLC1097 to verify the presence of the *MAL2p-CDC37* allele and with oLC1093/oLC1097 to ensure that there were no remaining wild-type alleles of *CDC37*. The *SAP2* promoter was then induced to drive expression of FLP recombinase to excise the NAT marker cassette, and the NAT-sensitive colonies were again PCR tested for the presence of the deleted allele using oLC1093/oLC1094, presence of the *MAL2* promoter using oLC441/oLC1097, and the absence of a wild-type allele using oLC1093/ oLC1097. The *TAR*-*tetO* cassette was amplified from pLC605 using oLC2208 and oLC2291 and transformed into the *MAL2p-CDC37/cdc37Δ* strain, which was grown in YPM before transformation. NAT-resistant and glucose-insensitive colonies were PCR tested for proper integration using oLC300/oLC705. The *SAP2* promoter was then induced to drive expression of FLP recombinase to excise the NAT marker cassette.

**CaLC4760:** The *tetO-HSF1/tetO-HSF1* strain in the SN250 background was generated using CRISPR technology adapted from Vyas *et al*, 2015[72]. The plasmid pLC970 containing the CRISPR machinery and guide directed to the promoter of *HSF1* was digested with KpnI-HF and SacI-HF to liberate the cassette. A repair template containing the *TAR-tetO* construct was PCR amplified from genomic DNA of the strain CaLC3786 (*tetO-HSP90/hsp90Δ HSF1-TAP/HSF1)* using the primers oLC4725 and oLC4726 which contain upstream and downstream homology to the promoter of *HSF1*. The digested plasmid and repair were transformed into CaLC2302 (SN250). NAT-resistant transformants were PCR tested for proper integration of the *tetO* promoter with the primers oLC4714/oLC1676 and PCR tested for the absence of wild-type alleles of *HSF1* using oLC989/oLC1676. The *SAP2* promoter was induced to drive expression of FLP recombinase to excise the NAT marker cassette. In order to reduce filamentation induced by *HSF1* overexpression to allow for more efficient excising of the NAT marker cassette, 20 μg/mL DOX was included in the YNB-BSA cultures. NAT-sensitive colonies were PCR tested with the primers oLC4714/oLC1676 to confirm integration of the *tetO* promoter and oLC989/oLC1676 to ensure that there were no wild-type alleles of *HSF1* remaining.

**CaLC5014:** The *tetO*-*HSF1-TAP/tetO-HSF1* strain in the SN250 background was constructed using the same method as CaLC2995, except that the *TAP-ARG4* cassette was transformed into CaLC4760 (SN250 *tetO-HSF1/tetO-HSF1*). Transformants were also PCR tested with the primers oLC4714/oLC1676 to confirm the presence of the *tetO* promoter and oLC989/oLC1676 to ensure that there were no wild-type alleles of the *HSF1* promoter remaining.

**CaLC4822:** The *tetO-HSF1/tetO-HSF1-TAP* strain was constructed using the same method as CaLC4760, except that the CRISPR machinery/sgRNA to *HSF1* and *TAR-tetO* repair were transformed into CaLC2993 (*HSF1-TAP/HSF1)*.

**CaLC4916:** The *tetO-HSF1/tetO-HSF1 NOP1-GFP* strain was constructed using the same method as CaLC4760, except that the CRISPR machinery/sgRNA to *HSF1* and *TAR-tetO* repair were transformed into CaLC4506 (*NOP1-GFP/NOP1)*.

**CaLC4931:** The *tetO-HSF1/tetO-HSF1 kex2Δ/kex2Δ* strain was constructed using the same method as CaLC4760, except that the CRISPR machinery/sgRNA to *HSF1* and *TAR-tetO* repair were transformed into CaLC3055 (*kex2Δ/kex2Δ*).

**CaLC4958:** The *tetO-HSF1/tetO-HSF1 efg1Δ/efg1Δ* strain was constructed using the same method as CaLC4760, except that the CRISPR machinery/sgRNA to *HSF1* and *TAR-tetO* repair were transformed into CaLC563 (*efg1Δ/efg1Δ*).

**CaLC4961:** The *tetO-HSF1/tetO-HSF1* strain in the CAI4 background was constructed using the same method as CaLC4760, except that the CRISPR machinery/sgRNA to *HSF1* and *TAR-tetO* repair were transformed into CaLC75 (CAI4).

**CaLC4963:** The *tetO-HSF1/tetO-HSF1 rob1Δ/rob1Δ* strain was constructed using the same method as CaLC4760, except that the CRISPR machinery/sgRNA to *HSF1* and *TAR-tetO* repair were transformed into CaLC2738 (*rob1Δ/rob1Δ*).

**CaLC5039:** The *tetO-HSF1/tetO-HSF1 ume6Δ/ume6Δ* (preflip) strain was constructed using the same method as CaLC4760, except that the CRISPR machinery/sgRNA to *HSF1* and *TAR-tetO* repair were transformed into CaLC1566 (*ume6Δ/ume6Δ*). Multiple attempts at excising the CRISPR machinery/sgRNA out of the strain were unsuccessful, so the strain remains NAT resistant.

**CaLC5275:** The *tetO-HSF1/tetO-HSF1* (preflip) strain was constructed using the same method as CaLC4760, except that the CRISPR machinery and sgRNA to *HSF1* were not excised from the strain. This was done to have a marker-matched control to compare with CaLC5039. This strain has the same phenotype as CaLC4760.

**CaLC4762:** The *tetO-HSF1/tetO-HSF1 brg1Δ/brg1Δ* strain was constructed using the same method as CaLC4760, except that the CRISPR machinery/sgRNA to *HSF1* and *TAR-tetO* repair were transformed into CaLC2736 (*brg1Δ/brg1Δ*).

**CaLC5277:** The *tetO-HSF1/tetO-HSF1 brg1Δ/brg1Δ* (preflip) strain was constructed using the same method as CaLC4762, except that the CRISPR machinery and sgRNA to *HSF1* were not excised from the strain. This was done to have a marker-matched strain to compare with CaLC5039. This strain has the same phenotype as CaLC4762.

**CaLC5042:** The *tetO-HSF1/tetO-HSF1* strain in the CAF2-1 background was constructed using the same method as CaLC4760, except that the CRISPR machinery/sgRNA to *HSF1* and *TAR-tetO* repair were transformed into CaLC2742 (CAF2-1).

**CaLC5077:** The *tetO-HSF1/tetO-HSF1 bub2Δ/bub2Δ* strain was constructed using the same method as CaLC4760, except that the CRISPR machinery/sgRNA to *HSF1* and *TAR-tetO* repair were transformed into CaLC1589 (*bub2Δ/bub2Δ*).

**CaLC5079:** The *tetO-HSF1/tetO-HSF1 ras1Δ/ ras1Δ* strain was constructed using the same method as CaLC4760, except that the CRISPR machinery/sgRNA to *HSF1* and *TAR-tetO* repair were transformed into CaLC564 (*ras1Δ/ ras1Δ)*.

**CaLC4892:** The *tetO-HSF1/tetO-HSF1-TAP ACT1p-HSP90/ACT1p-HSP90* strain was generated using CRISPR technology adapted from Vyas *et al*, 2015[72]. The plasmid pLC976 containing the CRISPR machinery and guide directed to the promoter of *HSP90* was digested with KpnI-HF and SacI-HF to liberate the CRISPR components. A repair template containing the *ACT1p* was PCR amplified from genomic DNA from the strain CaLC4828 (*ACT1p-HSP90*) using primers oLC5002 and oLC5003. The digested plasmid and repair were transformed into CaLC4822 (*tetO-HSF1/tetO-HSF1-TAP*). NAT-resistant transformants were PCR tested for proper integration using the primers oLC1850/oLC199 and for lack of wild-type *HSP90* promoter with the primers oLC198/oLC199. The *SAP2* promoter was induced to drive expression of FLP recombinase to excise the NAT marker cassette. NAT-sensitive colonies were additionally PCR tested with the primers oLC198 and oLC199 to ensure that there were no wild-type alleles of the *HSP90* promoter remained.

**CaLC4828:** To construct the *ACT1p-HSP90* strain the *ACT1* promoter with a NAT cassette was PCR amplified from plasmid pLC620 using the primers oLC5002 and oLC5003 and transformed into CaLC239 (SN95). NAT-resistant transformants were PCR tested for proper integration of the cassette using the primer pairs oLC274/oLC199 and oLC3116/oLC268. The *SAP2* promoter was induced to drive expression of FLP recombinase to excise the NAT marker cassette. NAT-sensitive colonies were additionally tested with oLC3116/oLC268 to verify the presence of the *ACT1* promoter.

**CaLC4855:** The *tetO-HSP90/tetO-HSP90* strain was generated using CRISPR technology adapted from Vyas *et al*, 2015[72]. The plasmid pLC976 containing the CRISPR machinery and guide directed to the promoter of *HSP90* was digested with KpnI-HF and SacI-HF to liberate the CRISPR components. A repair template containing the *TAR-tetO* promoter was PCR amplified from genomic DNA from the CaLC3786 strain (*tetO-HSP90/hsp90Δ HSF1-TAP/HSF1*) using primers oLC4980 and oLC4981 with homology to *HSP90*. The digested plasmid and repair were transformed into CaLC239 (SN95). NAT-resistant transformants were PCR tested for proper integration using the primers oLC4714/oLC199 and for lack of wild-type *HSP90* promoter with oLC268/oLC199. The *SAP2* promoter was induced to drive expression of FLP recombinase to excise the NAT marker cassette. NAT-sensitive colonies were additionally PCR tested with oLC4714/oLC199 and oLC268/oLC199.

**CaLC4790:** The *tetO-orf19*.*4021/tetO-orf19*.*4021* strain was generated using CRISPR technology adapted from Vyas *et al*, 2015[72]. The plasmid pLC971 containing the CRISPR machinery and guide directed to the promoter of *orf19*.*4021* was digested with KpnI-HF and SacI-HF to liberate the CRISPR components. A repair template containing the *TAR-tetO* promoter was PCR amplified from genomic DNA from the CaLC3786 strain (*tetO-HSP90/hsp90Δ HSF1-TAP/HSF1*) using primers oLC4810 and oLC4811 with homology to *orf19*.*4021*. The digested plasmid and repair were transformed into CaLC239 (SN95). NAT-resistant transformants were PCR tested for proper integration using the primers oLC300/oLC4813 and for lack of wild-type *orf19*.*4021* promoter with the primers oLC4812/oLC4813. The *SAP2* promoter was induced to drive expression of FLP recombinase to excise the NAT marker cassette.

**CaLC4791:** The *tetO-FOX2/tetO-FOX2* strain was generated using CRISPR technology adapted from Vyas *et al*, 2015[72]. The plasmid pLC972 containing the CRISPR machinery and guide directed to the promoter of *FOX2* was digested with KpnI-HF and SacI-HF to liberate the CRISPR components. A repair template containing the *TAR-tetO* promoter was PCR amplified from genomic DNA from the CaLC3786 strain (*tetO-HSP90/hsp90Δ HSF1-TAP/HSF1*) using primers oLC4802 and oLC4803 with homology to *FOX2*. The digested plasmid and repair were transformed into CaLC239 (SN95). NAT-resistant transformants were PCR tested for proper integration using the primers oLC300/ oLC4806 and for lack of wild-type *FOX2* promoter with oLC4807/oLC4806. The *SAP2* promoter was induced to drive expression of FLP recombinase to excise the NAT marker cassette.

### Plasmid construction

**pLC335:** Sequence homologous to the upstream region of *PHO23* was PCR amplified from SC5314 genomic DNA (CaLC155) using the primers oLC396/oLC397, digested with KpnI andApaI, and ligated into pLC49. Integration was PCR tested using the primers oLC275/oLC396. Sequence homologous to the downstream region of *PHO23* was PCR amplified from SC5314 genomic DNA using the primers LC398/oLC399, digested with SacI and SacII, and ligated into pLC49 containing the sequence homologous to the upstream region of *PHO23*. Integration was PCR tested using the primersoLC274/oLC399. The construct can be liberated using KpnI andSacI.

**pLC478:** Sequence homologous to the upstream region of *HSF1* was PCR amplified from SC5314 genomic DNA using the primers oLC985/oLC986, digested with ApaI, and ligated into pLC49. Integration was PCR tested using the primers oLC275/oLC985. Sequence homologous to the downstream region of *HSF1* was PCR amplified from SC5314 genomic DNA using the primers oLC987/oLC988, digested with SacI and SacII, and ligated into pLC49 containing the sequence homologous to the upstream region of *HSF1*. Integration was PCR tested using the primers oLC274/oLC988. The construct can be liberated using BssHII.

**pLC481:** Sequence homologous to the upstream region of *HSF1* was PCR amplified from SC5314 genomic DNA using the primers oLC991/oLC992, digested with ApaI, and ligated into pLC49. Integration was PCR tested using the primers oLC275/oLC991. Sequence homologous to the beginning of the *HSF1* open reading frame (beginning with ATG) was PCR amplified from SC5314 genomic DNA using the primers oLC993/oLC994, digested with SacI and SacII, and ligated into pLC49 containing the sequence homologous to the upstream region of *HSF1*. Integration was PCR tested using the primers oLC274/oLC994. The *MAL2* promoter was liberated from pLC50 using NotI and SacII and cloned into the plasmid containing both pieces of sequence homologous to *HSF1*. Integration was PCR tested using the primer pairs oLC441/oLC994 and oLC274/oLC994. The construct can be liberated using BssHII.

**pLC551:** The plasmid pLC330, containing the *tetO* promoter and sequence homologous to *HSP90*, was digested with SapI and ApaI to remove the sequence homologous to the upstream region of *HSP90*. Sequence homologous to the upstream region of *HSF1* was PCR amplified from SC5314 genomic DNA using the primers oLC1368/oLC1370, digested with SapI and ApaI, and ligated into pLC330 with the region of *HSP90* sequence removed. Next, the plasmid pLC52, containing the tetracycline-repressible transactivator *TAR*, was digested with ApaI to liberate *TAR* and ligated into the plasmid containing sequence homologous to *HSF1* and the *tetO* promoter. This plasmid was then digested with KpnI and SacII to remove the sequence homologous to the downstream region of *HSP90*. Sequence homologous to the downstream region of *HSF1* was PCR amplified from SC5314 genomic DNA using the primers oLC993/oLC1369, digested with KpnI and SacII, and ligated into the plasmid. The construct can be liberated using SapI/KpnI.

**pLC605:** The plasmid pLC52, containing the tetracycline-repressible transactivator and the *FLP-NAT* cassette, was digested with NotI and SacII. The *tetO* promoter was PCR amplified from pLC46 using the primers oLC300 and oLC301, digested with NotI and SacII and ligated into the plasmid.

**pLC755:** The plasmid pLC335, containing sequence homologous to upstream and downstream of *PHO23* was digested with NotI and SacII. *HSP90* was PCR amplified from pLC455 using the primers oLC1493 and oLC1485, digested with NotI and SacII and ligated into the pLC335 vector backbone. The plasmid containing *HSP90* was then digested with NotI. The *ACT1* promoter was PCR amplified from pLC430 using the primers oLC1482 and oLC1492, digested with NotI, and ligated into the plasmid. The *PHO23-ACT1p-HSP90-PHO23* cassette can be liberated using BssHII.

**pLC757:** The plasmid pLC430 containing the *ACT1* promoter, was digested with XmaI. *HSP90* was PCR amplified from pLC455 using the primers oLC1429 and oLC1430, digested with XmaI and ligated into the vector backbone. The entire integrating vector can be linearized with BsrGI to target it for integration at the *ACT1* locus.

**pLC963:** The sequences for two adjacent regions at a neutral locus on the left arm of chromosome 5 (Neut5L) (5-Neut: Ca22chr5A_C_albicans_SC5314:232638–233017 and 3-Neut: Ca22chr5A_C_albicans_SC5314:233018–233358) were synthesized by IDT. These fragments were cloned into pLC933 (pV1093[72]) at the KpnI and SacI/SacII sites, respectively, by Gibson Assembly. The Nat^R^ containing ApaI and NotI fragment was replaced with a FLP-Nat^R^ amplicon generated using the primers NatFLP-fwd and NatFLP-rev and pJK863[73] as a template.

**pLC970:** The plasmid pLC963 was digested with BsmBI and the sgRNA directed towards the *HSF1* promoter was inserted by ligating the annealed primers, oLC4723 and oLC4724. Proper integration was confirmed using Sanger sequencing with the primer oLC4609.

**pLC971:** The plasmid pLC963 was digested with BsmBI and the sgRNA directed towards the *orf19*.*4021* promoter was inserted by ligating the annealed primers, oLC4808 and oLC4809. Proper integration was confirmed using Sanger sequencing with the primer oLC4609.

**pLC972:** The plasmid pLC963 was digested with BsmBI and the sgRNA directed towards the *FOX2* promoter was inserted by ligating the annealed primers, oLC4804 and oLC4805. Proper integration was confirmed using Sanger sequencing with the primer oLC4609.

**pLC976:** The plasmid pLC963 was digested with BsmBI and the sgRNA directed towards the *HSP90* promoter was inserted by ligating the annealed primers, oLC4978 and oLC4979. Proper integration was confirmed using Sanger sequencing with the primer oLC4609.

## Supporting information

S1 Data Microarray data comparing the expression of genes in the *tetO HSF1/hsf1Δ* strain when grown in the absence and presence of DOX. (XLSX)

S2 Data ChIP-Seq data. List of basal and overexpression targets, GO terms associated with the targets, and all of the ChIP-Seq peaks.
(XLSX)

S3 Data MEME Analysis showing motif distributions around Hsf1 ChIP-Seq peak summits. (XLSX)

S4 Data RNA-seq data. List of the differentially expressed genes upon *HSF1* overexpression and all of the expression data.
(XLSX)

S1 Table Strains used in this study. (DOCX)

S2 Table Plasmids used in this study. (DOCX)

S3 Table Oligonucleotides used in this study. (DOCX)

S1 Fig Filamentation induced by *HSF1* overexpression is not a general property of genetic overexpression, and filamentation induced by *HSF1* overexpression or depletion occurs without substantial cell death. a) Strains were grown in rich medium for 6 hours at 30°C in the absence of DOX. Overexpression of *HSF1*, but not *orf19*.*4021*, *FOX2* or *HSP90*, induces filamentation. b) Overnights of the wild-type strain or *tetO-HSF1/tetO-HSF1* strain were subcultured to an OD of 0.1 in rich medium in the absence or presence of the indicated concentrations of DOX and grown overnight. Cultures were subcultured to an OD of 0.1 in the same conditions and grown for an additional overnight before imaging analysis. Heat killed cells were prepared by treating the wild-type strain at 100°C for five minutes. Cell death was assayed by treating 20 μL samples with 25 μg/mL of the membrane impermeable dye propidium iodide for 5 minutes in the dark before imaging. Images were all taken at the same fluorescence intensity.
(TIF)

S2 Fig Titration of *HSF1* levels with DOX highlights the relationship between *HSF1* expression and filamentation. Strains were grown in rich medium in the presence of no DOX, 0.01 μg/mL DOX, 0.1 μg/mL DOX, 1 μg/mL DOX or 10 μg/mL DOX at 30°C. a) Quantitative RT-PCR analysis to determine the levels of *HSF1* when grown in varying levels of DOX. *HSF1* transcript levels were normalized to *ACT1* and *GPD1*. Data are means +/- standard error of the means for triplicate samples. *** indicates P value <0.005, ** indicates P value <0.01, * indicates P value <0.05, ns indicates no significant difference, unpaired t test. b) *HSF1* overexpression induces filamentation when the *tetO-HSF1-TAP/tetO-HSF1* strain is grown in the presence of no DOX or 0.01 μg/mL DOX. When grown in 0.1 μg/mL DOX, where the *HSF1* levels are close to wild-type levels (no significant difference, P value 0.1773, unpaired t test), the strain grows in the yeast form. *HSF1* depletion induces filamentation when grown in the presence of 1 μg/mL DOX or 10 μg/mL DOX. The morphology of the control strain (*HSF1-TAP/HSF1*) is unaffected by growth in varying concentrations of DOX.
(TIF)

S3 Fig Overexpression or depletion of *HSF1* induces filamentation in multiple strain backgrounds. Strains were grown in the presence of no DOX, 0.1 μg/mL DOX, or 20 μg/mL DOX at 30°C. Strains in the CAI4 background were grown with 80 mg/L uridine added to the growth medium.
(TIF)

S4 Fig *HSF1* expression varies significantly under different conditions. a) A scatterplot showing the Fragments Per Kilobase of transcript per Million mapped reads (FPKM) values for *HSF1* gene expression in 169 published RNA-seq datasets of wild-type strains of *C*. *albicans* grown under different conditions. *HSF1* expression levels vary significantly under diverse experimental conditions. b) Quantitative RT-PCR analysis comparing the levels of *HSF1* in the control strain (*HSF1-TAP/HSF1*) grown in *HSF1* inducing experimental conditions and in the *HSF1* overexpression strains. *HSF1* expression was significantly induced in the wild-type strain upon a 30°C to 42°C heat shock or when grown in biofilms formed in Spider medium. While the levels of *HSF1* in these experimental conditions did not reach the levels of the *tetO-HSF1-TAP/tetO-HSF1* strain where filamentation is observed, the levels were comparable to the *tetO-HSF1-TAP/hsf1Δ* strain which filaments upon elevated temperature. Strains were grown in the presence of no DOX or 0.01 μg/mL DOX, as indicated. *HSF1* transcript levels were normalized to *ACT1* and *PMA1*. Data are means +/- standard error of the means for triplicate samples. *** indicates P value <0.005, ns indicates no significant difference, unpaired t test.
(TIF)

S5 Fig Reduced temperature does not block filamentation in response to multiple genetic perturbations. a) Filamentation induced by depletion of *HSP90* is not blocked at 23°C. Strains were grown in the absence or presence of 20 μg/mL DOX at the indicated temperatures. b) Filamentation in response to overexpression of positive filamentation regulators *BRG1* or *UME6* is not blocked at 23°C. Overexpression of *LEU3* serves as a control that has no impact on filamentation. Strains were grown at the indicated temperatures in the absence or presence of 50 μg/mL DOX to induce expression of the *tetON* promoter. c) Filamentation in response to homozygous deletion of *TUP1* or *NRG1* is not blocked at 23°C. Strains were grown in the absence of DOX at the indicated temperatures.
(TIF)

S6 Fig Ras1 is necessary for filamentation in response to *HSF1* depletion but dispensable for filamentation in response to *HSF1* overexpression. Strains were grown in rich medium with 80 mg/L uridine added, in the presence of no DOX, 0.1 μg/mL DOX, 20 μg/mL DOX or 50 μg/mL DOX at 30°C.
(TIF)

S7 Fig *HSF1* depletion induces filamentation in multiple conditional expression systems, and modulates the expression of genes involved in filamentation. a) The impact of *HSF1* depletion was assessed using a strain where the only allele of *HSF1* is under the control of the *tetO* promoter (left panels), allowing for transcriptional repression with DOX treatment. The effect of *HSF1* depletion was also monitored using a strain where the only allele of *HSF1* was under the control of the *MAL2* promoter (right panels), allowing for induction of *HSF1* expression in rich medium with maltose (YPM) and repression in rich medium with glucose (YPD). Filamentation is observed when *HSF1* expression is repressed through growth in 20 μg/mL DOX or 50% YPM/YPD, respectively. b) Microarray analysis identified transcriptional changes in response to *HSF1* depletion in the *tetO-HSF1/hsf1Δ* strain. GO terms associated with the genes misregulated upon *HSF1* depletion by greater than 2-fold are shown. c) Gene Set Enrichment Analysis identified transcriptional profiles that are significantly correlated to the transcriptional profile of genes up-regulated in response to *HSF1* depletion. Of note, the profile of genes upregulated in response to geldanamycin treatment was similar to the genes upregulated in response to *HSF1* depletion. d) Enrichment plot for geldanamycin.
(TIF)

S8 Fig *HSF1* depletion compromises Hsp90 function, leading to a small but reproducible decrease in phosphorylated Hog1 levels. Western blot analysis was performed to assay if *HSF1* depletion compromises Hsp90 function by monitoring the phosphorylation of the Hsp90 client protein Hog1. Strains were grown in the absence or presence of 20 μg/mL DOX. Cells were treated with 5 mM hydrogen peroxide (H_2_O_2_) for 10 minutes to induce oxidative stress before protein extraction. Depletion of Hsf1 reduces the levels of phosphorylated Hog1 (pHog1), even in the isogenic strain with constitutive *HSP90* expression. Tubulin or actin levels serve as loading controls. WT indicates the wild type, untagged control. Three biological replicates are shown.
(TIF)

S9 Fig *HSF1* depletion does not induce filamentation through reduced expression of any single Hsf1-dependent Hsp90 co-chaperone gene. Deletion or depletion mutants were made for the six Hsf1-dependent Hsp90 co-chaperone genes in *C*. *albicans*. Strains were grown in the absence or presence of high DOX (20 μg/mL) at 30°C as indicated. Scale bar represents 10 μm.
(TIF)

S10 Fig Expression analysis for *UME6* and *BRG1*. a) Quantitative RT-PCR analysis to validate the overexpression of *UME6* or *BRG1* in the tetracycline-inducible promoter (*tetON*) strains. Strains were grown in the absence or presence of 50 μg/mL DOX at 30°C to induce overexpression. Overexpression of *LEU3* acts as a negative control. b) Overexpression of *HSF1* no longer drives the overexpression of *UME6* or *BRG1* when grown at lower temperatures. The wild-type and *tetO-HSF1/tetO-HSF1* strains were grown at 30°C or 23°C, in the absence of DOX, until mid-log phase. *UME6*, *BRG1* and *HSF1* transcript levels were normalized to *ACT1* and *GPD1*. Data are means +/- standard error of the means for triplicate samples.
(TIF)
