# Supplementary material for: Tuning Hsf1 levels drives distinct fungal morphogenetic programs with depletion impairing Hsp90 function and overexpression expanding the target space
Source: PLoS Genet. 2018 Mar 28;14(3):e1007270. doi: 10.1371/journal.pgen.1007270 (PMC5873724; doi:10.1371/journal.pgen.1007270)
Supplement: S1 Table — (DOCX) [file pgen.1007270.s005.docx]

**S1 Table:** Strains used in this study.

| **Strain Name** | **Alias** | **Genotype** | **Source** |
| --- | --- | --- | --- |
| CaLC239 | SN95 | *arg4 /arg4 his1 /his1 URA3/ura3 ::imm^434^ IRO1/iro1::imm^434^* | [1] |
| CaLC2993 | SN95 *HSF1-TAP/HSF1* | *arg4/arg4 his1/his1 URA3/ura3::imm^434^ IRO1/iro1::imm^434^ HSF1-TAP-ARG4/HSF1* | [2] |
| CaLC970 | SN95 *HSF1/hsf1Δ* | *arg4/arg4 his1/his1 URA3/ura3::imm^434^ IRO1/iro1::imm^434^ HSF1/hsf1::FRT* | This study |
| CaLC2928 | SN95 *tetO-HSF1/hsf1Δ* | *arg4/arg4 his1/his1 URA3/ura3::imm^434^ IRO1/iro1::imm^434^ TAR-FRT::tetO-HSF1/hsf1::FRT* | This study |
| CaLC2995 | SN95 *tetO-HSF1-TAP/hsf1Δ* | *arg4/arg4 his1/his1 URA3/ura3::imm^434^ IRO1/iro1::imm^434^ TAR-FRT::tetO-HSF1-TAP-ARG4/hsf1::FRT* | This study |
| CaLC4822 | SN95 *tetO-HSF1-TAP/tetO-HSF1* | *arg4/arg4 his1/his1 URA3/ura3::imm^434^ IRO1/iro1::imm^434^ TAR-tetO-HSF1-TAP-ARG4/TAR-tetO-HSF1* | This study |
| CaLC2302 | SN250 (derived from SN152) | *arg4/arg4 his1/his1 leu2::C.dubliniensis HIS1/leu2::C.maltosa LEU2 URA3/ura3::imm^434^ IRO1/iro1::imm^434^* | [3] |
| CaLC4760 | SN250 *tetO-HSF1/tetO-HSF1* | *arg4/arg4 his1/his1 leu2::C.dubliniensis HIS1/leu2::C.maltosa LEU2 URA3/ura3 ::imm^434^ IRO1/iro1::imm^434^ TAR-tetO-HSF1/TAR-tetO-HSF1* | This study |
| CaLC5275 | SN250 *tetO-HSF1/tetO-HSF1* (preflip) | *arg4/arg4 his1/his1 leu2::C.dubliniensis HIS1/leu2::C.maltosa LEU2 URA3/ura3 ::imm^434^ IRO1/iro1::imm^434^*  *NEUT5L-FRT-ENO1p-Cas9-NAT-snR52p-HSF1p sgRNA-FRT/NEUT5L*  *TAR-tetO-HSF1/TAR-tetO-HSF1* | This study |
| CaLC4506 | SN95 *NOP1-GFP/NOP1* | *arg4 /arg4 his1 /his1 URA3/ura3::imm^434^ IRO1/iro1::imm^434^ NOP1/NOP1-GFP-HIS* | [4] |
| CaLC4916 | SN95 *tetO-HSF1/tetO-HSF1 NOP1-GFP/NOP1* | *arg4 /arg4 his1 /his1 URA3/ura3::imm^434^ IRO1/iro1::imm^434^ NOP1/NOP1-GFP-HIS TAR-tetO-HSF1/TAR-tetO-HSF1* | This study |
| CaLC75 | CAI4 | *ura3::imm^434^/ura3::imm^434^* | [5] |
| CaLC4961 | CAI4 *tetO-HSF1/tetO-HSF1* | *ura3::imm^434^/ura3::imm^434^ TAR-tetO-HSF1/TAR-tetO-HSF1* | This study |
| CaLC564 | CAI4 *ras1Δ/ras1Δ* | *ura3::imm^434^/ura3::imm^434^ ras1::hisG/ras1::hph* | [6] |
| CaLC5079 | CAI4 *tetO-HSF1/tetO-HSF1 ras1Δ/ras1Δ* | *ura3::imm^434^/ura3::imm^434^ ras1D::hisG/ras1D::hph TAR-tetO-HSF1/TAR-tetO-HSF1* | This study |
| CaLC3017 | SN95 *tetO-HSF1/hsf1Δ ACT1p-HSP90/ACT1p-HSP90* | *arg4/arg4 his1/his1 URA3/ura3::imm^434^ IRO1/iro1::imm^434^ TAR-tetO-HSF1/hsf1::FRT pho23::ACT1p-HSP90::FRT/PHO23 ACT1p-HSP90/ACT1* | This study |
| CaLC3890 | SN95 *tetO-HSF1-TAP/hsf1Δ ACT1p-HSP90/ACT1p-HSP90* | *arg4/arg4 his1/his1 URA3/ura3::imm^434^ IRO1/iro1::imm^434^ TAR-tetO-HSF1-TAP/hsf1::FRT pho23::ACT1p-HSP90::FRT/PHO23 ACT1p-HSP90/ACT1* | This study |
| CaLC3384/3385 | SN95 *tetO-HSP90/hsp90Δ* | *arg4 /arg4 his1 /his1 URA3/ura3::imm^434^ IRO1/iro1 ::imm^434^ hsp90::FRT/TAR-FRT::tetO-HSP90* | This study |
| CaLC3786 | SN95 *tetO-HSP90/hsp90Δ HSF1-TAP/HSF1* | *arg4 /arg4 his1 /his1 URA3/ura3::imm^434^ IRO1/iro1 ::imm^434^ hsp90::FRT/TAR-FRT::tetO-HSP90 HSF1/HSF1-TAP-ARG4* | This study |
| CaLC3991 | SN95  *tetO-CDC37/cdc37Δ* | *URA3/ura3 ::imm^434^ IRO1/iro1 ::imm^434^ cdc37::FRT/TAR-FRT::tetO-CDC37* | This study |
| CaLC3171 | SN95  *cpr6Δ/cpr6Δ* | *URA3/ura3 ::imm^434^ IRO1/iro1 ::imm^434^ cpr6::FRT/cpr6::FRT* | This study |
| CaLC3172 | SN95  *aha1Δ/aha1Δ* | *URA3/ura3 ::imm^434^ IRO1/iro1 ::imm^434^ aha1::FRT/aha1::FRT* | This study |
| CaLC3193 | SN95  *hch1Δ/hch1Δ* | *URA3/ura3 ::imm^434^ IRO1/iro1 ::imm^434^ hch1::FRT/hch1::FRT* | This study |
| CaLC3310 | SN95  *sti1Δ/sti1Δ* | *URA3/ura3 ::imm^434^ IRO1/iro1 ::imm^434^ sti1::FRT/sti1::FRT* | This study |
| CaLC3797 | SN95  *sba1Δ/sba1Δ* | *URA3/ura3 ::imm^434^ IRO1/iro1 ::imm^434^ sba1::FRT/sba1::FRT* | This study |
| CaLC1589 | RM1000  *bub2Δ/bub2Δ* | *ura3::imm^434^/ura3::imm^434^ his1::hisG/his1::hisG bub2::URA3/bub2::HIS1* | [7] |
| CaLC5077 | RM1000  *tetO-HSF1/tetO-HSF1 bub2Δ/bub2Δ* | *ura3::imm^434^/ura3::imm^434^ his1::hisG/ his1::hisG bub2::URA3/bub2::HIS1 TAR-tetO-HSF1/TAR-tetO-HSF1* | This study |
| CaLC563 | CAI4 *efg1Δ/efg1Δ* | *ura3::imm^434^/ura3::imm^434^ efg1::hisG/efg1::hisG-URA3-hisG* | [8] |
| CaLC4958/4959 | CAI4  *tetO-HSF1/tetO-HSF1 efg1Δ/efg1Δ* | *ura3::imm434/ura3::imm434 efg1::hisG/efg1::hisG-URA3-hisG TAR-tetO-HSF1/TAR-tetO-HSF1* | This study |
| CaLC2738 | SN152  *rob1Δ/rob1Δ* | *arg4/arg4 leu2/leu2 his1/his1 URA3/ura3::imm^434^ IRO1/iro1::imm^434^ rob1::C.albicansHIS1/rob1::C.albicans LEU2* | [9] |
| CaLC4963 | SN152  *tetO-HSF1/tetO-HSF1 rob1Δ/rob1Δ* | *arg4/arg4 leu2/leu2 his1/his1 URA3/ura3::imm^434^ IRO1/iro1::imm^434^ rob1::C.albicansHIS1/rob1::C.albicans LEU2 TAR-tetO-HSF1/TAR-tetO-HSF1* | This study |
| CaLC3055 | SN152  *kex2Δ/kex2Δ* | *arg4/arg4 his1/his1 leu2/leu2 URA3/ura3 ::imm^434^ IRO1/iro1::imm^434^ kex2::C.dubliniensis HIS1/kex2::C.maltosa LEU2* | [3] |
| CaLC4931 | SN152  *tetO-HSF1/tetO-HSF1 kex2Δ/kex2Δ* | *arg4/arg4 his1/his1 leu2/leu2 URA3/ura3 ::imm^434^ IRO1/iro1::imm^434^ kex2::C.dubliniensis HIS1/kex2::C. maltosa LEU2 TAR-tetO-HSF1/TAR-tetO-HSF1* | This study |
| CaLC4828 | SN95 *ACT1p-HSP90* | *arg4/arg4 his1/his1 URA3/ura3::imm^434^ IRO1/iro1::imm^434^ ACT1p-HSP90/HSP90* | This study |
| CaLC4892 | SN95  *tetO-HSF1-TAP/tetO-HSF1 ACT1p-HSP90/ACT1p-HSP90* | *arg4/arg4 his1/his1 URA3/ura3::imm^434^ IRO1/iro1::imm^434^ TAR-tetO-HSF1/TAR-tetO-HSF1-TAP-ARG4 ACT1p-HSP90/ACT1p-HSP90* | This study |
| CaLC5012 | SN152  *HSF1-TAP/HSF1* | *arg4/arg4 his1/his1 leu2::C.dubliniensis HIS1/leu2 ::C.maltosa LEU2 URA3/ura3::imm^434^ IRO1/iro1::imm^434^ HSF1-TAP-ARG4/HSF1* | This study |
| CaLC5014 | SN152  *tetO-HSF1-TAP/tetO-HSF1* | *arg4/arg4 his1/his1 leu2::C.dubliniensis HIS1/leu2 ::C.maltosa LEU2 URA3/ura3::imm^434^ IRO1/iro1::imm^434^ TAR-tetO-HSF1-TAP-ARG4/TAR-tetO-HSF1* | This study |
|  | *tetO-UME6* | *ura3::imm^434^/ura3::imm^434^ his1::hisG/HIS1 arg4::hisG/ARG4 ADH1/adh1::ADH1p-cartTA::SAT1::PTET-caUME6* | [10] |
|  | *tetO-BRG1* | *ura3::imm^434^/ura3::imm^434^ his1::hisG/HIS1 arg4::hisG/ARG4 ADH1/adh1::ADH1p-cartTA::SAT1::PTET-caBRG1* | [10] |
|  | *tetO-LEU3* | *ura3::imm^434^/ura3::imm^434^ his1::hisG/HIS1 arg4::hisG/ARG4 ADH1/adh1::ADH1p-cartTA::SAT1::PTET-caLEU3* | [10] |
| CaLC1566 | SN152 *ume6Δ/ume6Δ* | *ume6::C. maltosa LEU2/ume6::C. dubliniensis HIS1 arg4::ARG4/arg4 leu2/leu2 his1/his1 ura3::imm434/URA3 iro1::imm434/IRO1* | [11] |
| CaLC5039 | SN152 *tetO-HSF1/tetO-HSF1 ume6Δ/ume6Δ* (preflip) | *ume6::C. maltosa LEU2/ume6 :: C. dubliniensis HIS1 arg4 ::ARG4/arg4 leu2 /leu2 his1/his1 ura3 ::imm^434^/URA3 iro1 ::imm^434^/IRO1*  *NEUT5L-FRT-ENO1p-Cas9-NAT-snR52p-HSF1p sgRNA-FRT/NEUT5L*  *TAR-tetO-HSF1/TAR-tetO-HSF1* | This study |
| CaLC2736 | SN152 *brg1Δ/brg1Δ* | *arg4/arg4 leu2/leu2 his1/his1 URA3/ura3::imm^434^ IRO1/iro1::imm^434^ brg1::C. albicans HIS1/brg1::C. albicans LEU2* | [9] |
| CaLC4762 | SN152 *tetO-HSF1/tetO-HSF1 brg1Δ/brg1Δ* | *arg4/arg4 leu2/leu2 his1/his1 URA3/ura3::imm^434^ IRO1/iro1 ::imm^434^ brg1::C. albicans HIS1/ brg1::C. albicans LEU2 TAR-tetO- HSF1/TAR-tetO-HSF1* | This study |
| CaLC5277 | SN152 *tetO-HSF1/tetO-HSF1 brg1Δ/brg1Δ* (preflip) | *arg4/arg4 leu2/leu2 his1/his1 URA3/ura3::imm^434^ IRO1/iro1 ::imm^434^ brg1::C. albicans HIS1/ brg1::C. albicans LEU2 NEUT5L-FRT-ENO1p-Cas9-NAT-snR52p-HSF1p sgRNA-FRT/NEUT5L*  *TAR-tetO-HSF1/TAR-tetO-HSF1* | This study |
| CaLC2689 | SN95 *tup1Δ/tup1Δ* | *arg4/arg4 his1/his1 URA3/ura3::imm^434^ IRO1/iro1::imm^434^ tup1::FRT/tup1::FRT* | [12] |
| CaLC3050 | SN152 *nrg1Δ/nrg1Δ* | *arg4/arg4 leu2/leu2 his1/his1 URA3/ura3::imm^434^ IRO1/iro1::imm^434^ nrg1::C. albicans HIS1/nrg1::C. albicans LEU2* | [9] |
| CaLC4855 | SN95 *tetO-HSP90/tetO-HSP90* | *arg4/arg4 his1/his1 URA3/ura3::imm^434^ IRO1/iro1::imm434 TAR-tetO-HSP90/TAR-tetO-HSP90* | This study |
| CaLC4790 | SN95  *tetO-orf19.4021/tetO-orf19.4021* | *arg4/arg4 his1/his1 URA3/ura3::imm^434^ IRO1/iro1 ::imm^434^ TAR- tetO-orf19.4021/TAR- tetO-orf19.4021* | This study |
| CaLC4791 | SN95  *tetO-FOX2/tetO-FOX2* | *arg4/arg4 his1/his1 URA3/ura3::imm^434^ IRO1/iro1 ::imm^434^ TAR- tetO-FOX2/TAR- tetO-FOX1* | This study |
| CaLC974 | SN95 *MAL2p-HSF1/hsf1Δ* | *arg4/arg4 his1/his1 URA3/ura3::imm^434^ IRO1/iro1::imm^434^ MAL2p-HSF1/hsf1::FRT* | This study |
| CaLC2742 | CAF2-1 | *ura3::imm^434^/URA3* | [5] |
| CaLC5042 | CAF2-1 *tetO-HSF1/tetO-HSF1* | *ura3::imm^434^/URA3 TAR-tetO-HSF1/TAR-tetO-HSF1* | This study |
| CaLC155 | SC5314 |  | [13] |

**S1 Table References**

1. Noble SM, Johnson AD. Strains and strategies for large-scale gene deletion studies of the diploid human fungal pathogen *Candida albicans*. Eukaryotic Cell. 2005;4(2):298-309.

2. Leach MD, Budge S, Walker L, Munro C, Cowen LE, Brown AJP. Hsp90 orchestrates transcriptional regulation by Hsf1 and cell wall remodelling by MAPK signalling during thermal adaptation in a pathogenic yeast. PLoS Pathogens. 2012;8:e1003069.

3. Noble SM, French S, Kohn La, Chen V, Johnson AD. Systematic screens of a *Candida albicans* homozygous deletion library decouple morphogenetic switching and pathogenicity. Nature Genetics. 2010;42:590-8.

4. Xie JL, O'Meara TR, Polvi EJ, Robbins N, Cowen LE. Staurosporine induces filamentation in the human fungal pathogen *Candida albicans* via signaling through Cyr1 and Protein Kinase A. mSphere. 2017;2:1-14.

5. Fonzi WA, Irwin MY. Isogenic strain construction and gene mapping in *Candida albicans*. Genetics. 1993;134:717-28.

6. Feng Q, Summers E, Guo B, Fink G. Ras signaling is required for serum-induced hyphal differentiation in *Candida albicans*. Journal of Bacteriology. 1999;181:6339-46.

7. Bachewich C, Whiteway M. Cyclin Cln3p links G1 progression to hyphal and pseudohyphal development in *Candida albicans*. Eukaryotic Cell. 2005;4(1):95-102.

8. Braun BR, Johnson AD. *TUP1*, *CPH1*, and *EFG1* make independent contributions to filamentation in *Candida albicans*. Genetics. 2000;12:57-67.

9. Homann OR, Dea J, Noble SM, Johnson AD. A phenotypic profile of the *Candida albicans* regulatory network. PLoS Genetics. 2009;5(12):e1000783.

10. Chauvel M, Nesseir A, Cabral V, Znaidi S, Goyard S, Bachellier-Bassi S, et al. A versatile overexpression strategy in the pathogenic yeast *Candida albicans*: Identification of regulators of morphogenesis and fitness. PLoS One. 2012;7(9):e45912.

11. Banerjee M, Kadosh D, Thompson DS, Lazzell A, Carlisle PL. *UME6*, a novel filament-specific regulator of *Candida albicans* hyphal extension and virulence. Molecular Biology of the Cell. 2008;19:1354-65.

12. Diezmann S, Leach MD, Cowen LE. Functional divergence of Hsp90 genetic interactions in biofilm and planktonic cellular states. PLoS One. 2015;10(9):e0137947.

13. Gillum AM, Tsay EY, Kirsch DR. Isolation of the *Candida albicans* gene for orotidine-5'-phosphate decarboxylase by complementation of *S. cerevisiae* *ura3* and *E. coli* *pyrF* mutations. Molecular & General Genetics. 1984;198(2):179-82.
