# Supplementary material for: Tuning Hsf1 levels drives distinct fungal morphogenetic programs with depletion impairing Hsp90 function and overexpression expanding the target space
Source: PLoS Genet. 2018 Mar 28;14(3):e1007270. doi: 10.1371/journal.pgen.1007270 (PMC5873724; doi:10.1371/journal.pgen.1007270)
Supplement: S2 Table — (DOCX) [file pgen.1007270.s006.docx]

**S2 Table:** Plasmids used in this study.

| **Name** | **Description** | **Source** |
| --- | --- | --- |
| pLC330 | *tetO-CaHSP90, ampR natR* | [1] |
| pLC335 | *FLP-NAT -CaPHO23 K/O, ampR natR* | This study |
| pLC430 | *ACT1p-NAT, ampR natR* | [2] |
| pLC455 | *CaHSP90 complementation vector, ampR natR* | [3] |
| pLC46 | *pNIM1, ampR* | [4] |
| pLC478 | *FLP-NAT-HSF1-KO, ampR natR* | This study |
| pLC481 | *FLP-NAT-MAL2p-HSF1, ampR natR* | This study |
| pLC49 | *FLP-NAT, ampR natR* | [2] |
| pLC50 | *FLP-NAT-MAL2p, ampR natR* | [2] |
| pLC505 | *FLP-NAT-CDC37-KO, ampR natR* | [5] |
| pLC506 | *FLP-NAT-MAL2p-CDC37, ampR natR* | [5] |
| pLC52 | *tet-R* (*TAR*)*, ampR natR* | [1] |
| pLC551 | *CaTAR-FLP-NAT-tetO-HSF1, ampR natR* | This study |
| pLC573 | *pFA-TAP-ARG4, ampR* | [6] |
| pLC605 | *CaTAR-FLP-NAT-tetO, ampR natR* | This study |
| pLC62 | *FLP-NAT-HSP90-KO, ampR natR* | [1] |
| pLC620 | *NAT-ACT1-3xFLAG, ampR natR* | [7] |
| pLC755 | *FLP-NAT PHO23-ACT1p-HSP90-PHO23, ampR natR* | This study |
| pLC757 | *NAT-ACT1p-HSP90, ampR natR* | This study |
| pLC933 | pV1093, Solo system CaCas9/sgRNA entry expression vector, contains NatR  gene, and 2kb targeting arms for the  upstream and downstream of *ENO1* coding region, ampR | [8] |
| pLC963 | pV1393, as pLC933 but with targeting arms for the upstream and downstream of *NEUT5L,* ampR natR | This study |
| pLC970 | pV1393 with sgRNA targeting Ca*HSF1* promoter, ampR natR | This study |
| pLC971 | pV1393 with sgRNA targeting Ca*orf19.4021* promoter, ampR natR | This study |
| pLC972 | pV1393 with sgRNA targeting Ca*FOX2* promoter, ampR natR | This study |
| pLC976 | pV1393 with sgRNA targeting Ca*HSP90* promoter, ampR natR | This study |

**S2 Table References**

1. Shapiro RS, Uppuluri P, Zaas AK, Collins C, Senn H, Perfect JR, et al. Hsp90 orchestrates temperature-dependent *Candida albicans* morphogenesis via Ras1-PKA signaling. Current Biology. 2009;19:621-9.

2. Shen J, Guo W, Köhler JR. Ca*NAT1*, a heterologous dominant selectable marker for transformation of *Candida albicans* and other pathogenic *Candida* species. Infection and Immunity. 2005;73(2):1239-42.

3. Cowen LE, Singh SD, Köhler JR, Collins C, Zaas AK, Schell Wa, et al. Harnessing Hsp90 function as a powerful, broadly effective therapeutic strategy for fungal infectious disease. Proceedings of the National Academy of Sciences of the United States of America. 2009;106:2818-23.

4. Park Y-N, Morschhäuser J. Tetracycline-inducible gene expression and gene deletion in *Candida albicans*. Eukaryotic Cell. 2005;4(8):1328-342.

5. Shapiro RS, Zaas AK, Betancourt-Quiroz M, Perfect JR, Cowen LE. The Hsp90 co-chaperone Sgt1 governs *Candida albicans* morphogenesis and drug resistance. PloS One. 2012;7:e44734.

6. Lavoie H, Sellam A, Askew C, Nantel A, Whiteway M. A toolbox for epitope-tagging and genome-wide location analysis in *Candida albicans*. BMC Genomics. 2008;9:578.

7. Shapiro RS, Sellam A, Tebbji F, Whiteway M, Nantel A, Cowen LE. Pho85, Pcl1, and Hms1 signaling governs *Candida albicans* morphogenesis induced by high temperature or Hsp90 compromise. Current Biology. 2012;22:461-70.

8. Vyas VK, Barrasa MI, Fink GR. A *Candida albicans* CRISPR system permits genetic engineering of essential genes and gene families. Science Advances. 2015;1:e1500248.
