# Supplementary material for: Tuning Hsf1 levels drives distinct fungal morphogenetic programs with depletion impairing Hsp90 function and overexpression expanding the target space
Source: PLoS Genet. 2018 Mar 28;14(3):e1007270. doi: 10.1371/journal.pgen.1007270 (PMC5873724; doi:10.1371/journal.pgen.1007270)
Supplement: S3 Table — (DOCX) [file pgen.1007270.s007.docx]

**S3 Table:** Oligonucleotides used in this study.

| Name | Description | Sequence (5' -> 3') |
| --- | --- | --- |
|  | NatFLP-fwd | GACGCTCGAAGGCTTTAATTTGCGGCCGGGCCCCCCCTCGAGGGATCCACTAGTTCTAG |
|  | NatFLP-rev | TTTGAACGGACTAAGTCTAATCACTTGCGGCCGCACATCAAGCTTGCCTCGTCCCCGCCG |
| oLC1093 | CaCDC37-645-F | CCATCTCCAGGTAACAACTC |
| oLC1094 | CaCDC37+2150-R | CCACAATCTTATGGTCAACC |
| oLC1097 | CaCDC37+662-R | GGAGCTTTTGGTTTATCTTG |
| oLC1368 | CaHSF1-619-SapI-F | TAGCGCTCTTCCGTGTCCATAATCCCTGATTTC |
| oLC1369 | CaHSF1+350-KpnI-R | CGGGGTACCGAGTTGTATTCATCTCCTTCC |
| oLC1370 | CaHSF1-322-ApaI-R | TTGCGGGCCCGACAAGAAGCAAGTCTTAAC |
| oLC1429 | CaHSP90+1-Xma1-F | CATTCCCGGGATGGCTGACGCAAAAGTTGA |
| oLC1430 | CaHSP90+2124-Xma1-R | CATTCCCGGGTTAATCAACTTCTTCCATAGC |
| oLC1460 | UME6+1648-F | GGAGGTGTAAGTAATGACAC |
| oLC1461 | UME6+1976-R | GGTTCTTCATTGACAACTTG |
| oLC1482 | CaACT1-1012- NotI-F | AGAATGCGGCCGCGCTATTAAGATCACCAGCCT |
| oLC1485 | CaHSP90-2124-SacII-R | TCCCCGCGGAACAAGATCAATACCTTACTACC |
| oLC1492 | CaACT1-1-NotI-R | AGAATGCGGCCGCTTTGAATGATTATATTTTTTTAATATTAA |
| oLC1493 | CaHSP90+1-NotI-F | AGAATGCGGCCGCATGGCTGACGCAAAAGTTG |
| oLC1593 | TAP-R | TAAACTTTGGATGAAGGCG |
| oLC1594 | ARG4-F | ATGTTGGCTACTGATTTAGCTG |
| oLC1597 | CaHSF1+1978-F | GGACAATCAATTCAGCAAGTG |
| oLC1598 | CaHSF1+2607-R | GCATTTACTCTCAATAGAGTG |
| oLC1676 | CaHSF1+231-R | CCTGCATCTGGGTATAGTTTGG |
| oLC1850 | ACT1p-R | CGT TAA TTA GTT GTT CAA CG |
| oLC198 | CaHSP90-131F | GTGGTCTCCGTTGGTTGCC |
| oLC199 | CaHSP90+307R | CAGATTTAGCAATAGTACCC |
| oLC2208 | CaCDC37_pLC605F | TCAAATTTTTTTAGCTTTCCCACTTGATTTCTTCTTGTATTGGCTACCTAAATTGTAATTAAGATCAATCGGAAACAGCTATGACCATG |
| oLC2285 | CaACT1+855-F | GACCTTGAGATACCCAATTG |
| oLC2286 | CaACT1+1076-R | CAGCTTGAATGGAAACGTAG |
| oLC2291 | CaCDC37 tetO R | TTGGATGAACTTCCACATCAGAATCATCAGAAATTTCTATCTTATCCCACTTGGAGTAATCTATTGGCATCGACTATTTATATTTGTATG |
| oLC2451 | CaHSF1+721-F | CCATCAGATGGTATTTCTTCC |
| oLC2452 | CaHSF1+931-R | GGTGAAACACTTGGAAAGTC |
| oLC2635 | CaBRG1 +462F | CCACAATCAGTTTCTGCTTCTAC |
| oLC2636 | CaBRG1 +727R | GACCTTGATGTGTTACAGATGG |
| oLC2677 | CaAHA1_+835F | TTGTTGAATTATGGAGATTAG |
| oLC2678 | CaAHA1_+1187R | TGAGCGAGTTGTATATGGTAG |
| oLC268 | CaHSP90-FP-1 | CGGAGATGTATTGACTGTGG |
| oLC2703 | CaSTI1_+1474F | AGGCAAGAACTAAAGATGTTG |
| oLC2704 | CaSTI1_+1877R | TTTGCCTATGTATATGCTAAC |
| oLC274 | pJK863down-F | CTGTCAAGGAGGGTATTCTGG |
| oLC275 | pJK863up-R | AAAGTCAAAGTTCCAAGGGG |
| oLC276 | CaHSP90-427-F | GAGATGGCAAATCGTTAGG |
| oLC277 | CaHSP90+2627-R | CCACCCTGAACCTAATTTGG |
| oLC2922 | CaHSF1-HA ARG R | TATACCTAAACAACGAAAATAATGAATTGAAAAATATAATGATTATAGACACACTTCATCTATATCCTATTCGATGAATTCGAGCTCGTT |
| oLC294 | CaHSP90-597-F-KpnI | CGGGGTACCGATTTCAGGTTGAAGAATTTGC |
| oLC2950 | CaHSF1-TAP-ARG4-F | TAACATAAATGATTCTAATGGTAATGAGAAGAAACTGAAGAAAAGATCAATCGAAGAAGTTAGCGATCATGGTCGACGGATCCCCGGGTT |
| oLC297 | CaHSP90+348-R-SacI | CCCGAGCTCTCAGCACCAGCACTTAAAGC |
| oLC300 | Tetp-F-NotI | ATAAGAATGCGGCCGCGTTTGGTTCAGCACCTTGTCG |
| oLC301 | Tetp-R-SacII | GGCACCGCGGCGACTATTTATATTTGTATGTGTGTAGG |
| oLC3022 | CaACT1-1127-F | GTTTCTTTGCCTTACTACCAC |
| oLC3058 | CaAHA1-KO-pLC49-F | TTGCACCGAATCGAGATTTTTCTTGAAGTAATCAACCTCTCAAAATCAAAACTAAAACAACATAGCAATGGGAAACAGCTATGACCATG |
| oLC3059 | CaAHA1-KO-pLC49-R | ATCATAAGTGATGGTTAATAGAGTAAAATTCTTCTTTATGGATTGCTTGTACGTACATTCTATTTATCTAGTAAAACGACGGCCAG |
| oLC3062 | CaCPR6-KO-pLC49-F | TTTTCCAAATTTATATAAAAATGAGATAAAATTTTCGTTTCTGGTATTTTTCTTTTTCCACCAACTCATGGGAAACAGCTATGACCATG |
| oLC3063 | CaCPR6-KO-pLC49-R | GTATGTCGCACGACTTTTTGTTGAAAAAAAAAAAAAAGCTTTTATTATCATCTATGAAGGCCTATATGATTTAGTAAAACGACGGCCAG |
| oLC3064 | CaCPR6-352-F | TTCCGGGTTGCCTAGTCTTT |
| oLC3065 | CaCPR6+1735-R | AAACTGCACTTCCTTGACGG |
| oLC3067 | CaSBA1-KO-pLC49-R | TGACTAATAAAATAGACTATTCAACTGTATAATTTTGGTTTTTTTTGTATTACTTTGTTATTTGAGATTAGTAAAACGACGGCCAG |
| oLC3074 | CaSTI1-KO-pLC49-F | AACTTCCACCCAAATATCAAATAACTAACTTATCATTCCAACAGATAATATTCCCACTTCAATAACAATGGGAAACAGCTATGACCATG |
| oLC3075 | CaSTI1-KO-pLC49-R | CAACAACTTGTGTAAATAAACCAAATGTGAATTATAAGGGGGTTTTTATTAGTTACTTTTTATCTGGTTAGTAAAACGACGGCCAG |
| oLC3076 | CaSTI1-294-F | TGGCCTTCTGTGTCAAATTGT |
| oLC3086 | CaHCH1-KO-pLC49-F | ATTTTTCACTTTTAAACTTCATAGTATTAACGGTACTATTTGAGTAGTAACTTTCAACAAACAAAACATGGGAAACAGCTATGACCATG |
| oLC3087 | CaHCH1-KO-pLC49-R | TGTTATGTAATTTAAGTAACTATCCACAAGGTATGTATATACACATATGTATTTAGTGTAAATTCTATTAGTAAAACGACGGCCAG |
| oLC3088 | CaHCH1-615-F | CCTTTGATGGTTGGTGCTTT |
| oLC3089 | CaHCH1+1111-R | ACTTTTGCCTGCCTCAAGAA |
| oLC3116 | M13R+34R | CTTTAGTGAGGGTTAATTGC |
| oLC3170 | CaSBA1-407-F | TGATGTTCAGAGGTAGGCTT |
| oLC3172 | CaAHA1-300-F | TTCTCATTTGGCATGCTGTT |
| oLC3220 | CaHSP90-tetO-pLC605-R | TAATGATCAAAGACATCAACTGAGAGATCTCAGCAGTGAATTCGTGAGTTTCAACTTTTGCGTCAGCCATCGACTATTTATATTTGTATG |
| oLC324 | CaHSP90 + 2104F | GCTATGGAAGAAGTTGATTAA |
| oLC3390 | CaHSP90-tetO-pLC605-F | GAAGAAGTGGTGATATGAGAAACAACCACCAGCAGCGCCACCAGCAGCACCATATTACTACCTATAACAGGGAAACAGCTATGACCATG |
| oLC3747 | CaSBA1+831-R | GGGTTTGCAAATACCTAATG |
| oLC3748 | CaSBA1-B-KO pLC49 F | TAATAATACTCAAATTGAAGGCTTTCACATTCTGAAATAGAACATTAATTGAAGAAGAAAAATTACCCATTGGAAACAGCTATGACCATG |
| oLC376 | CaPHO23-A | CTAAAATTTCAGACTAAACG |
| oLC381 | CaPHO23-D | CATTTACGTAATTACCATTGC |
| oLC396 | CaPHO23-375-F-KpnI | CGGGGTACCCAACACTAGTAATATTATCC |
| oLC397 | CaPHO23+3-R-ApaI | TTGCGGGCCCCATTGTTAAGAATTACCTG |
| oLC398 | CaPHO23+1447-F-SacII | TCCCCGCGGTAACACCAATAAAACCAAGAG |
| oLC399 | CaPHO23+1810-R-SacI | CCCGAGCTCTTCCGTTCTTGTCTTTTCC |
| oLC408 | CaHSP90 + 536R | AACAATCTCAACATGGTACC |
| oLC441 | CaMAL2-528-F-NotI | ATAAGAATGCGGCCGCGTCTAGTACCATCTGTACC |
| oLC4609 | snr52p-fwd1 | GAAACTTCGGCCCAATAGGATTGG |
| oLC4714 | tetOp+488F | TCGTTTCTGATGGGCTTTTC |
| oLC4723 | CaHSF1p_sgRNA_top | ATTTGAAATATAGAAACTGTTAATGG |
| oLC4724 | CaHSF1p_sgRNA_bot | AAAACCATTAACAGTTTCTATATTTC |
| oLC4725 | CaHSF1p_CaTAR+tetOp_fwd | CTTAATTTAACATCTCGAGATATTTCTTCCTCAACAACCTGGAAACAGCTATGACCATG |
| oLC4726 | CaHSF1orf_CaTAR+tetOp_rev | GTAATGGATCTCTATAATCAGTAGTCATATTCATAATCATCGACTATTTATATTTGTATG |
| oLC4802 | CaFOX2p_cTAR+tetOp_f | TTCTTTTTCTTTTTMTTCTTTTTCTTTTTCCTTTTAATCCTGGAAACAGCTATGACCATG |
| oLC4803 | CaFOX2ORF_cTAR+tetOp_r | GGTGATGATAACAACTTTATCTTTAAAATCTATTGGAGACATCGACTATTTATATTTGTA |
| oLC4804 | CaFOX2p_sgRNA_top | ATTTGAATAAAATAGAATATTTCAAG |
| oLC4805 | CaFOX2p_sgRNA_bot | AAAACTTGAAATATTCTATTTTATTC |
| oLC4806 | CaFOX2+396-R | AGCCTTGGTGACAGCGTAAG |
| oLC4807 | CaFOX2-464-F | ACCAGTTTACTGTGGTTTGC |
| oLC4808 | Caorf19.4021p_sgRNA_top | ATTTGATCACTCATGTTTCGATGTAG |
| oLC4809 | Caorf19.4021p_sgRNA_bot | AAAACTACATCGAAACATGAGTGATC |
| oLC4810 | Ca-orf19.4021p_cTAR+tetOp_f | GAAAAAAAAAAACAACATAAACAGCTGAAATTTCATTTCCAGGAAACAGCTATGACCATG |
| oLC4811 | Caorf19.4021ORF_cTAR+tetOp_r | CTTCCATAGCCACGTCTCTATCATATTCCAAATCACTCATCGACTATTTATATTTGTATG |
| oLC4812 | Ca-orf19.4021-328-F | TTCACTGTCATGCTGGGATG |
| oLC4813 | Ca-orf19.4021+232-R | GTGTGCTGCATTGATAGGAC |
| oLC4978 | CaHSP90p_sgRNA_top | ATTTGTTGATTTGTATGGTATGATTG |
| oLC4979 | CaHSP901p_sgRNA_bot | AAAACAATCATACCATACAAATCAAC |
| oLC4980 | CaHSP90p_CaTAR+tetOp_fwd | CCTCCCGTTTTTTCTTTTTTTCATTTCTTCTTTCTATCCAGGAAACAGCTATGACCATG |
| oLC4981 | CaHSP90orf_CaTAR+tetOp_rev | CAGCAGTGAATTCGTGAGTTTCAACTTTTGCGTCAGCCATCGACTATTTATATTTGTATG |
| oLC5002 | CaHSP90+pLC620F | CCTCCCGTTTTTTCTTTTTTTCATTTCTTCTTTCTATCCAAATCATACCATACAAATCAATAGTTCATTGGAAACAGCTATGACCATG |
| oLC5003 | CaHSP90+pLC620R | CATCAACTGAGAGATCTCAGCAGTGAATTCGTGAGTTTCAACTTTTGCGTCAGCCATTTTGAATGATTATATTTTTTTAATATTAATATC |
| oLC534 | CaTAR-797-R | GATGGAGATAGTTTACGG |
| oLC609 | CaHSP90+308R-SacI | CGAGCTCCCAGATTTAGCAATAGTACC |
| oLC6472 | CaPMA1AB+720-F | TGGTGACTCTACTTTCGTCG |
| oLC6473 | CaPMA1AB+869-R | AACAAGCAACCCAAACGACC |
| oLC705 | CaCDC37+333-R | CAAGGTCAGACCCTTGTCTT |
| oLC752 | CaGPD1A+570-F | AGTATGTGGAGCTTTACTGGGA |
| oLC753 | CaGPD1AB+766-R | CAGAAACACCAGCAACATCTTC |
| oLC756 | CaHSP90+1051-F | GCTGAAGAGTTGATTCCAGAAT |
| oLC757 | CaHSP90+1236-R | GGAGAAAGCAGTGTAGAATTGG |
| oLC985 | CaHSF1-378-ApaI-F | TTGCGGGCCCCCTAAATACACTCACTGTCTAC |
| oLC986 | CaHSF1+3-ApaI-R | TTGCGGGCCCCATACAAAAACGAATATAAATATAG |
| oLC987 | CTA8+2284-SacII-F | TCCCCGCGGTAAATAGGATATAGATGAAGTG |
| oLC988 | CaHSF1+2693-SacI-R | CCCGAGCTCCGTCATCAATTGGATTTTGC |
| oLC989 | CaHSF1-523-F | CCAACTTGCAATTATAACCG |
| oLC990 | CaHSF1+2890-R | GCTGTCTTTCTACTTTATCG |
| oLC991 | CaHSF1-619-ApaI-F | TTGCGGGCCCCGTGTCCATAATCCCTGATTTC |
| oLC992 | CaHSF1-252-ApaI-R | TTGCGGGCCCGTTGTACTATGTGTTTGTGG |
| oLC993 | CaHSF1+1-SacII-F | TCCCCGCGGATGATTATGAATATGACTACTG |
| oLC994 | CaHSF1+350-SacI-R | CCCGAGCTCGAGTTGTATTCATCTCCTTCC |
| oLC995 | CaHSF1-762-F | GCTAAATTGTACATTTTCACC |
| oLC996 | CaHSF1+499-R | CGATTAACTCGTGATGTTGTTG |
|  |  |  |
|  |  |  |
