# Supplementary figures and images for: Tuning Hsf1 levels drives distinct fungal morphogenetic programs with depletion impairing Hsp90 function and overexpression expanding the target space
Source: PLoS Genet. 2018 Mar 28;14(3):e1007270. doi: 10.1371/journal.pgen.1007270 (PMC5873724; doi:10.1371/journal.pgen.1007270)

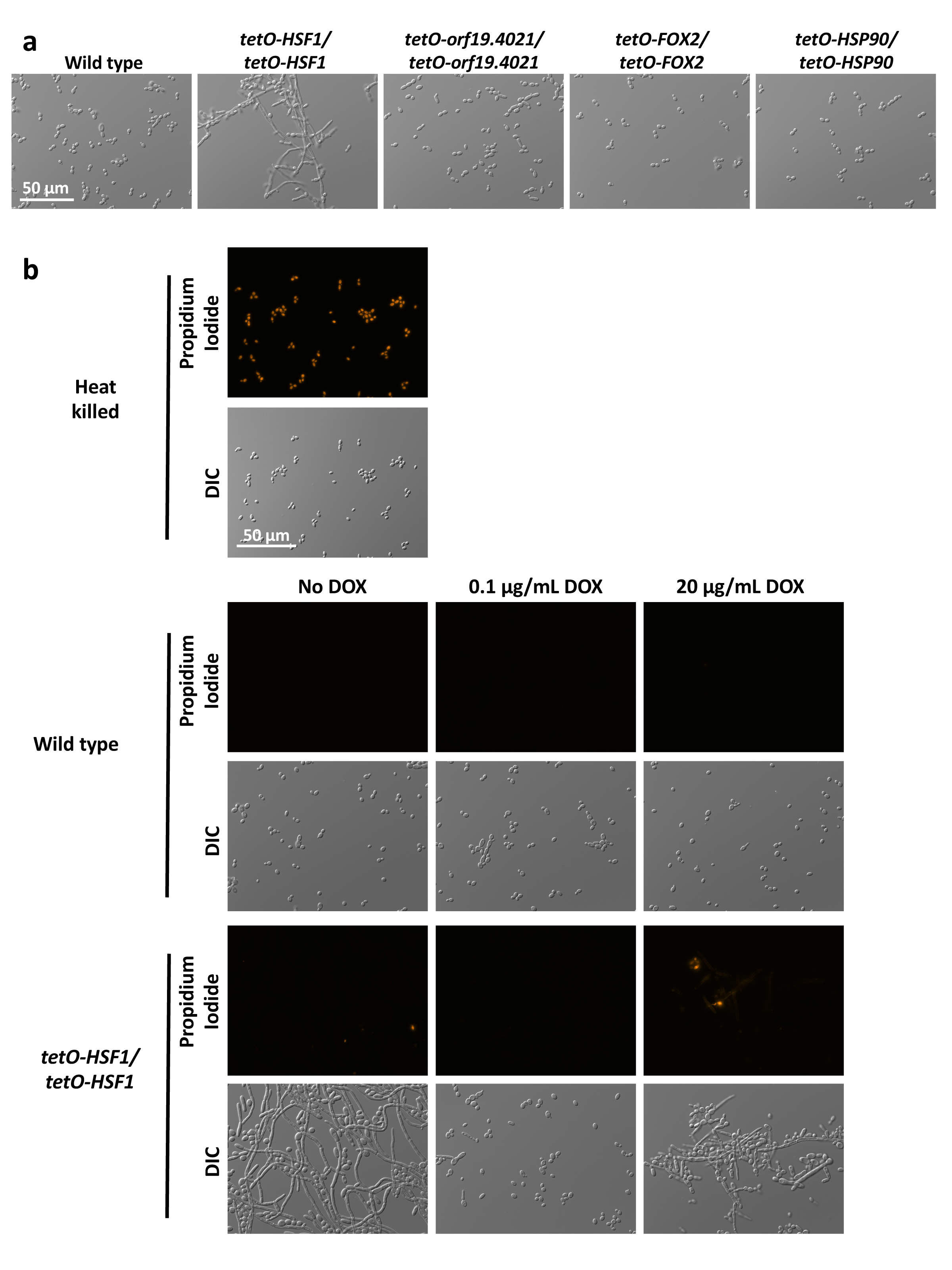

Supplement: S1 Fig — a) Strains were grown in rich medium for 6 hours at 30°C in the absence of DOX. Overexpression of HSF1, but not orf19.4021, FOX2 or HSP90, induces filamentation. b) Overnights of the wild-type strain or tetO-HSF1/tetO-HSF1 strain were subcultured to an OD of 0.1 in rich medium in the absence or presence of the indicated concentrations of DOX and grown overnight. Cultures were subcultured to an OD of 0.1 in the same conditions and grown for an additional overnight before imaging analysis. Heat killed cells were prepared by treating the wild-type strain at 100°C for five minutes. Cell death was assayed by treating 20 μL samples with 25 μg/mL of the membrane impermeable dye propidium iodide for 5 minutes in the dark before imaging. Images were all taken at the same fluorescence intensity. (TIF) [file pgen.1007270.s008.tif]

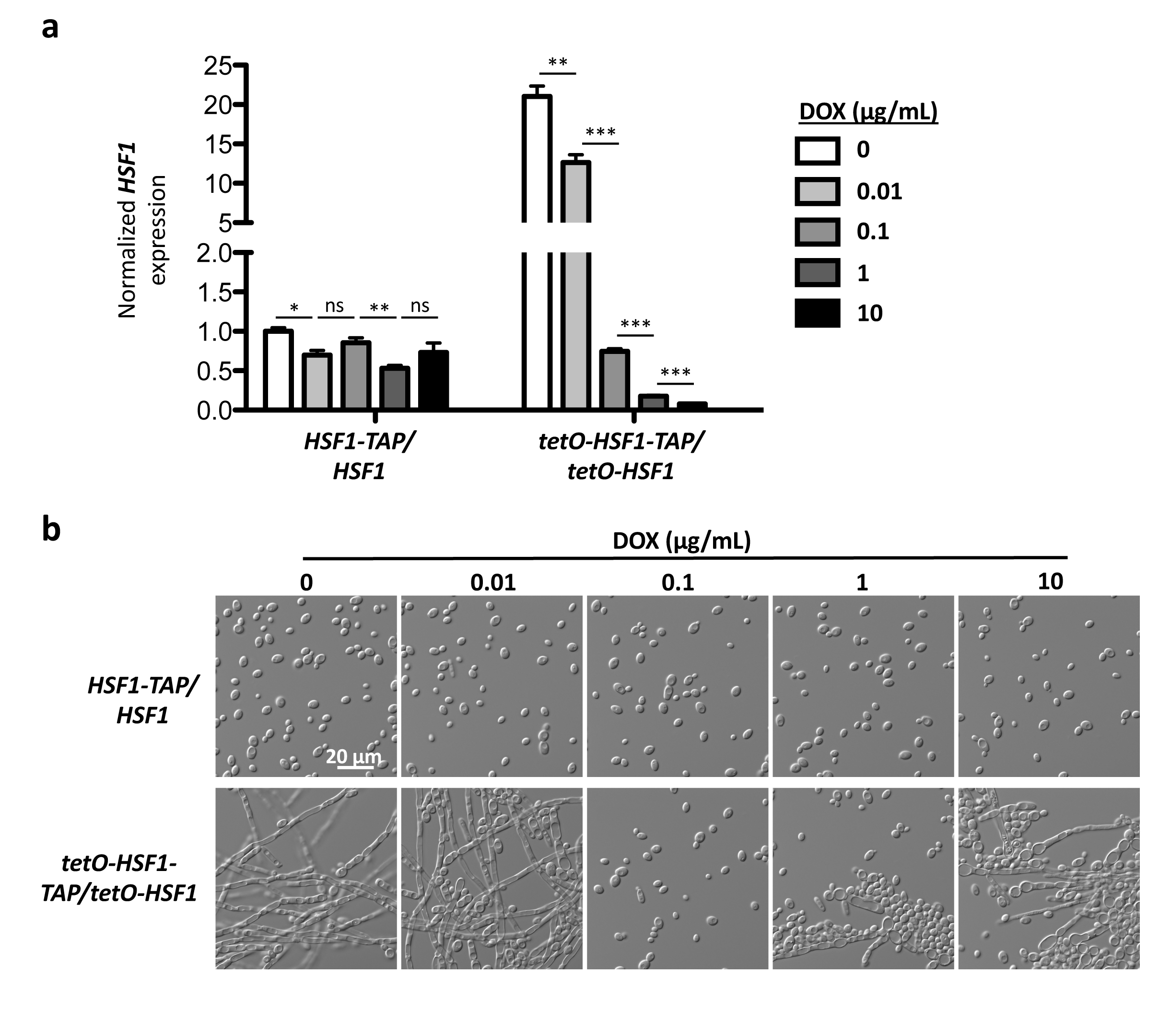

Supplement: S2 Fig — Strains were grown in rich medium in the presence of no DOX, 0.01 μg/mL DOX, 0.1 μg/mL DOX, 1 μg/mL DOX or 10 μg/mL DOX at 30°C. a) Quantitative RT-PCR analysis to determine the levels of HSF1 when grown in varying levels of DOX. HSF1 transcript levels were normalized to ACT1 and GPD1. Data are means +/- standard error of the means for triplicate samples. *** indicates P value <0.005, ** indicates P value <0.01, * indicates P value <0.05, ns indicates no significant difference, unpaired t test. b) HSF1 overexpression induces filamentation when the tetO-HSF1-TAP/tetO-HSF1 strain is grown in the presence of no DOX or 0.01 μg/mL DOX. When grown in 0.1 μg/mL DOX, where the HSF1 levels are close to wild-type levels (no significant difference, P value 0.1773, unpaired t test), the strain grows in the yeast form. HSF1 depletion induces filamentation when grown in the presence of 1 μg/mL DOX or 10 μg/mL DOX. The morphology of the control strain (HSF1-TAP/HSF1) is unaffected by growth in varying concentrations of DOX. (TIF) [file pgen.1007270.s009.tif]

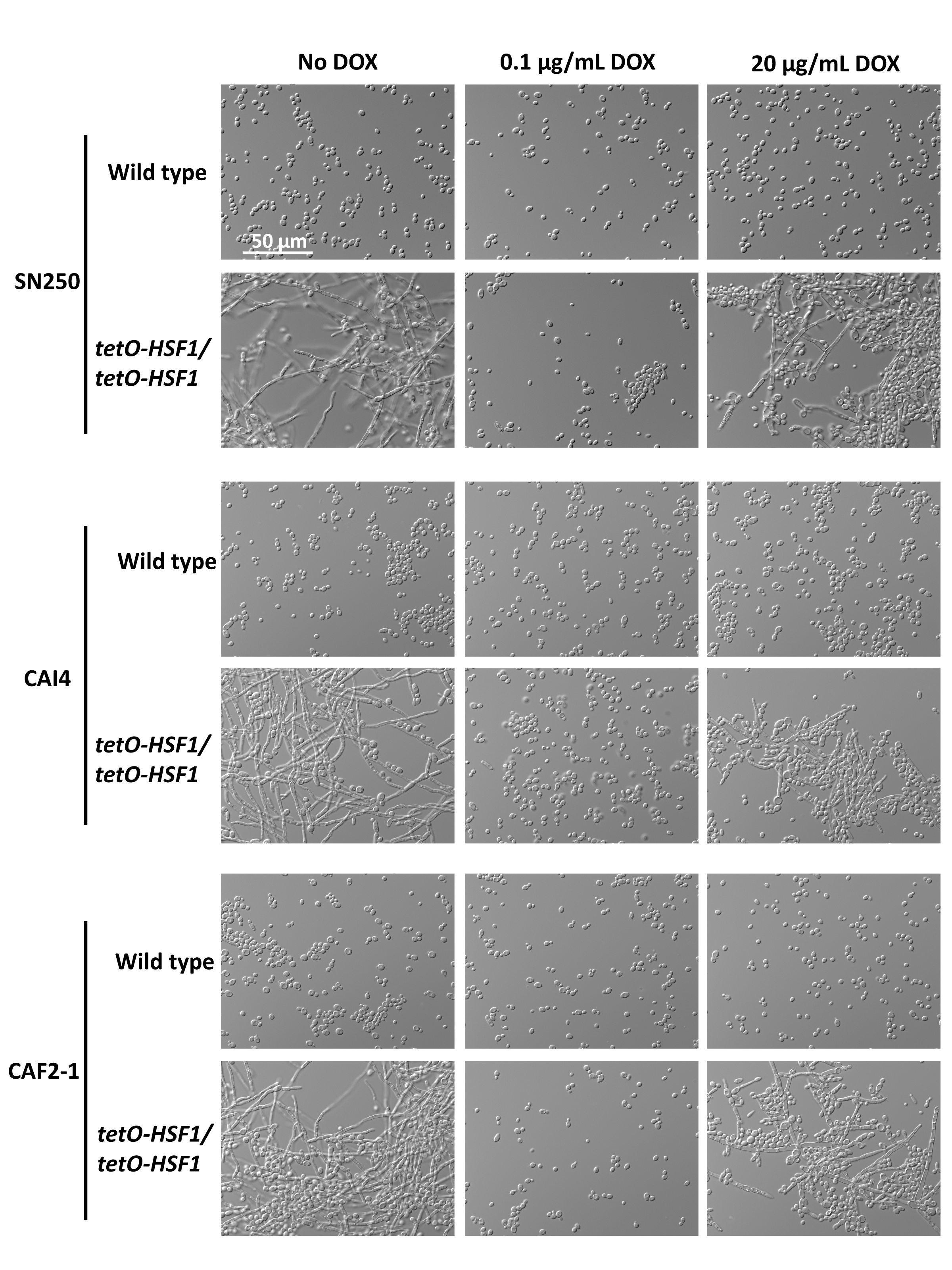

Supplement: S3 Fig — Strains were grown in the presence of no DOX, 0.1 μg/mL DOX, or 20 μg/mL DOX at 30°C. Strains in the CAI4 background were grown with 80 mg/L uridine added to the growth medium. (TIF) [file pgen.1007270.s010.tif]

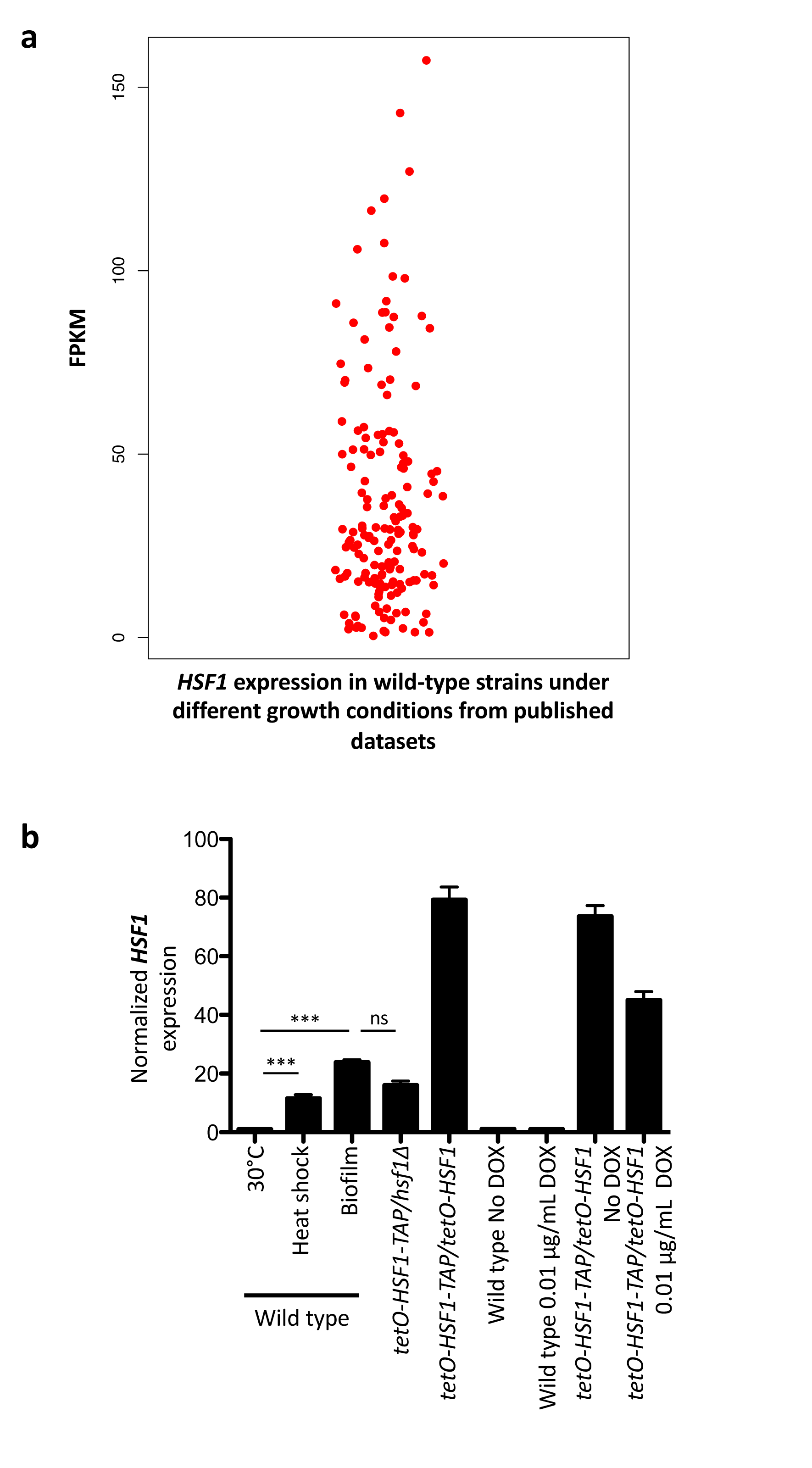

Supplement: S4 Fig — a) A scatterplot showing the Fragments Per Kilobase of transcript per Million mapped reads (FPKM) values for HSF1 gene expression in 169 published RNA-seq datasets of wild-type strains of C. albicans grown under different conditions. HSF1 expression levels vary significantly under diverse experimental conditions. b) Quantitative RT-PCR analysis comparing the levels of HSF1 in the control strain (HSF1-TAP/HSF1) grown in HSF1 inducing experimental conditions and in the HSF1 overexpression strains. HSF1 expression was significantly induced in the wild-type strain upon a 30°C to 42°C heat shock or when grown in biofilms formed in Spider medium. While the levels of HSF1 in these experimental conditions did not reach the levels of the tetO-HSF1-TAP/tetO-HSF1 strain where filamentation is observed, the levels were comparable to the tetO-HSF1-TAP/hsf1Δ strain which filaments upon elevated temperature. Strains were grown in the presence of no DOX or 0.01 μg/mL DOX, as indicated. HSF1 transcript levels were normalized to ACT1 and PMA1. Data are means +/- standard error of the means for triplicate samples. *** indicates P value <0.005, ns indicates no significant difference, unpaired t test. (TIF) [file pgen.1007270.s011.tif]

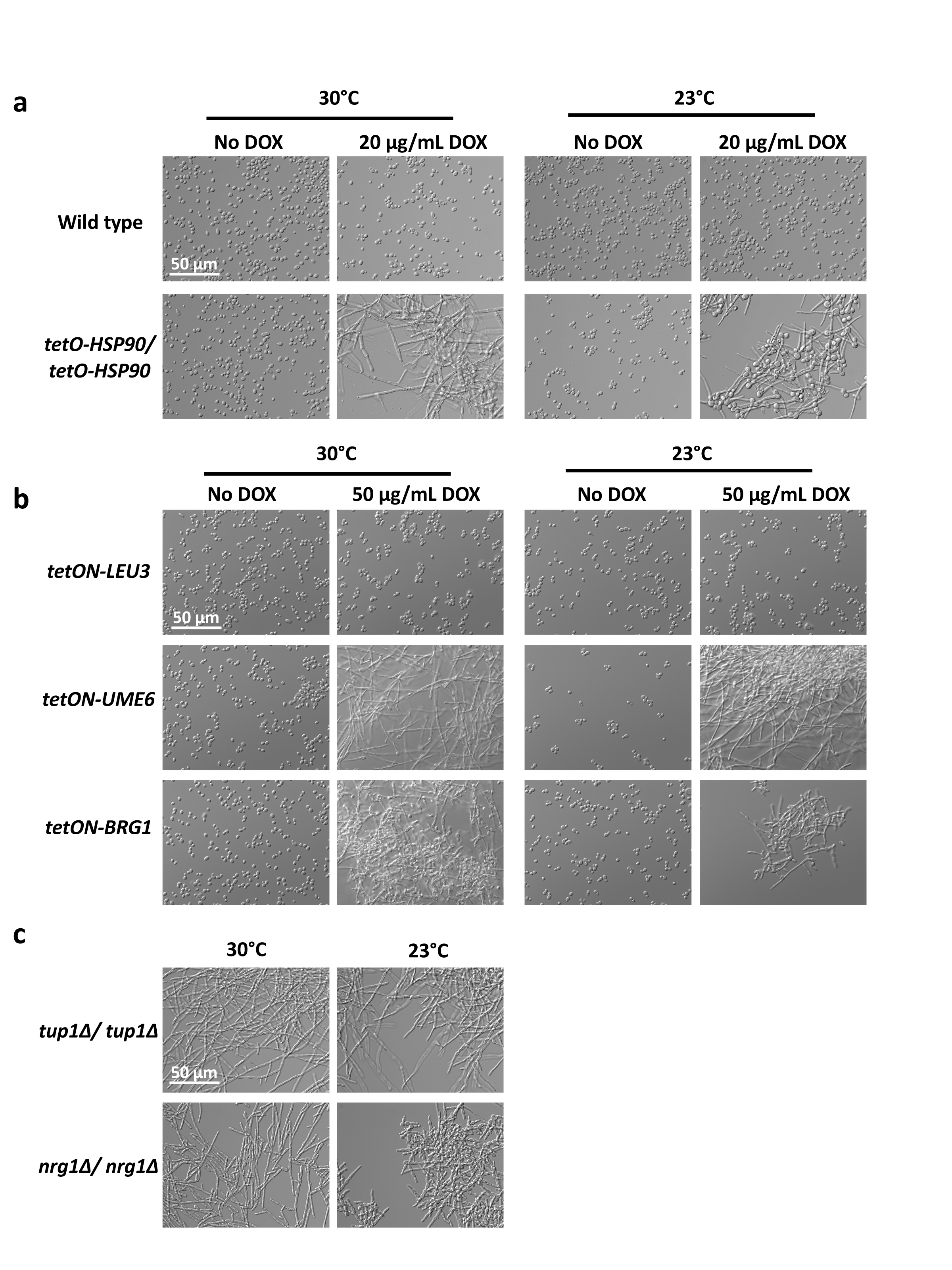

Supplement: S5 Fig — a) Filamentation induced by depletion of HSP90 is not blocked at 23°C. Strains were grown in the absence or presence of 20 μg/mL DOX at the indicated temperatures. b) Filamentation in response to overexpression of positive filamentation regulators BRG1 or UME6 is not blocked at 23°C. Overexpression of LEU3 serves as a control that has no impact on filamentation. Strains were grown at the indicated temperatures in the absence or presence of 50 μg/mL DOX to induce expression of the tetON promoter. c) Filamentation in response to homozygous deletion of TUP1 or NRG1 is not blocked at 23°C. Strains were grown in the absence of DOX at the indicated temperatures. (TIF) [file pgen.1007270.s012.tif]

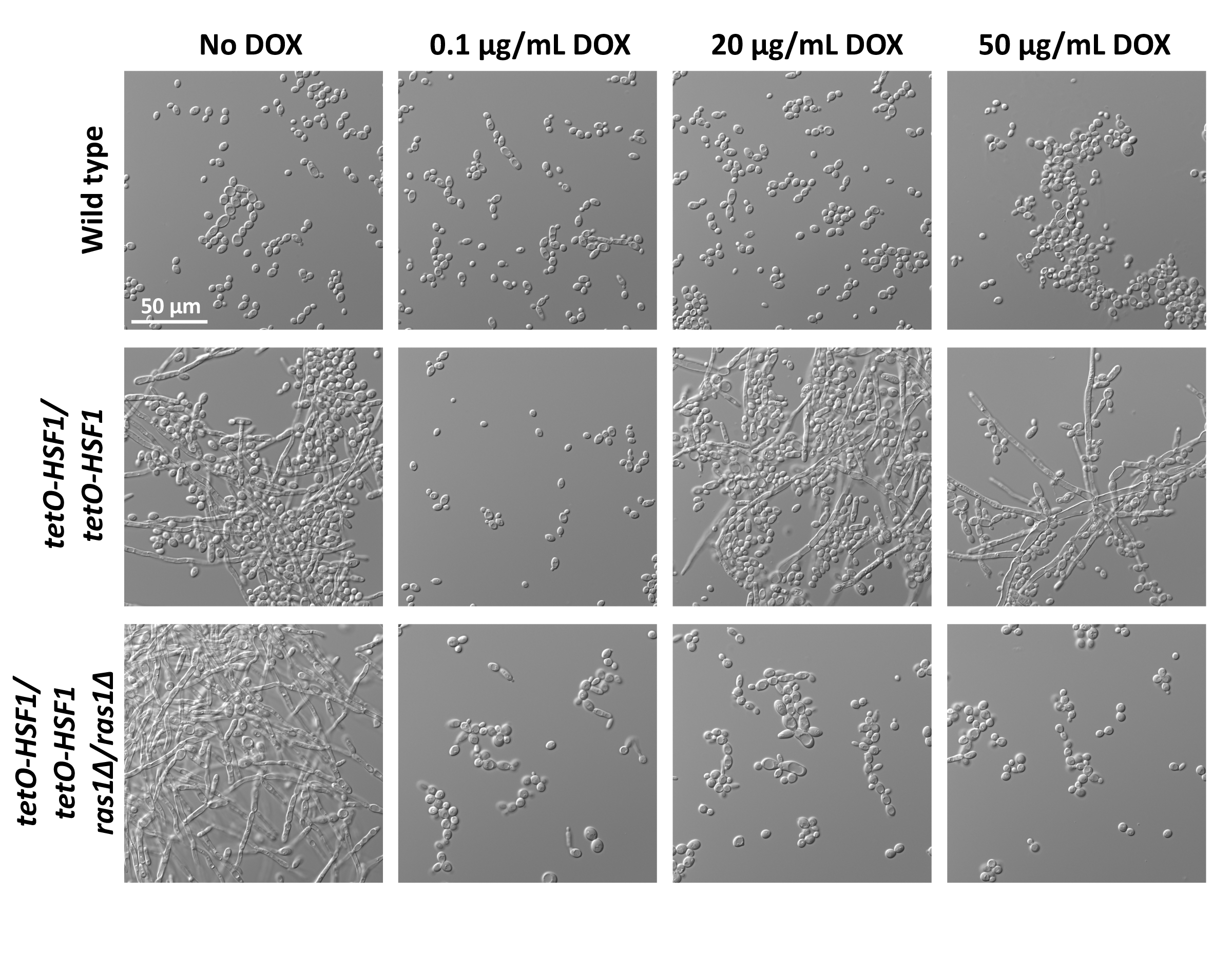

Supplement: S6 Fig — Strains were grown in rich medium with 80 mg/L uridine added, in the presence of no DOX, 0.1 μg/mL DOX, 20 μg/mL DOX or 50 μg/mL DOX at 30°C. (TIF) [file pgen.1007270.s013.tif]

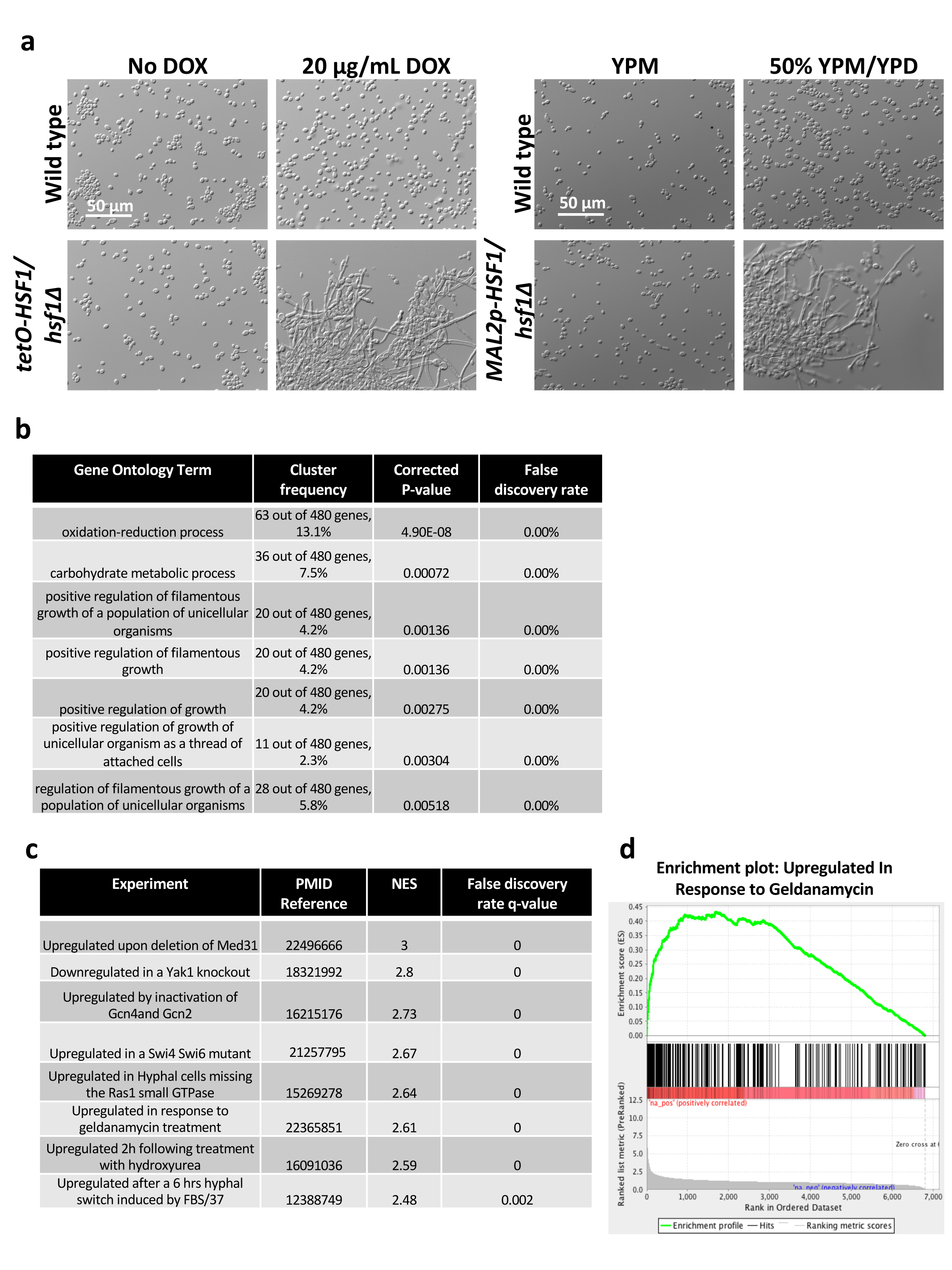

Supplement: S7 Fig — a) The impact of HSF1 depletion was assessed using a strain where the only allele of HSF1 is under the control of the tetO promoter (left panels), allowing for transcriptional repression with DOX treatment. The effect of HSF1 depletion was also monitored using a strain where the only allele of HSF1 was under the control of the MAL2 promoter (right panels), allowing for induction of HSF1 expression in rich medium with maltose (YPM) and repression in rich medium with glucose (YPD). Filamentation is observed when HSF1 expression is repressed through growth in 20 μg/mL DOX or 50% YPM/YPD, respectively. b) Microarray analysis identified transcriptional changes in response to HSF1 depletion in the tetO-HSF1/hsf1Δ strain. GO terms associated with the genes misregulated upon HSF1 depletion by greater than 2-fold are shown. c) Gene Set Enrichment Analysis identified transcriptional profiles that are significantly correlated to the transcriptional profile of genes up-regulated in response to HSF1 depletion. Of note, the profile of genes upregulated in response to geldanamycin treatment was similar to the genes upregulated in response to HSF1 depletion. d) Enrichment plot for geldanamycin. (TIF) [file pgen.1007270.s014.tif]

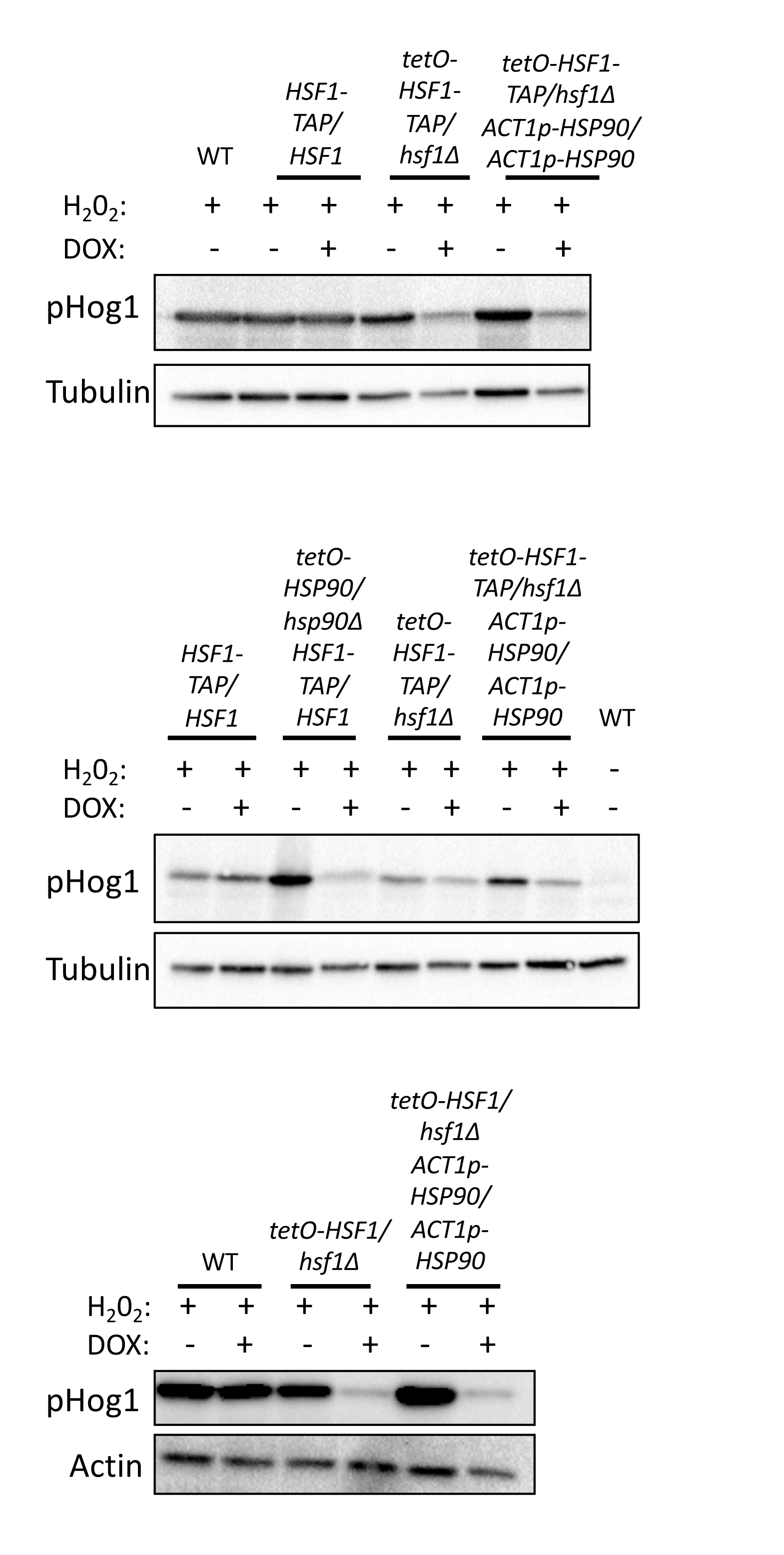

Supplement: S8 Fig — Western blot analysis was performed to assay if HSF1 depletion compromises Hsp90 function by monitoring the phosphorylation of the Hsp90 client protein Hog1. Strains were grown in the absence or presence of 20 μg/mL DOX. Cells were treated with 5 mM hydrogen peroxide (H2O2) for 10 minutes to induce oxidative stress before protein extraction. Depletion of Hsf1 reduces the levels of phosphorylated Hog1 (pHog1), even in the isogenic strain with constitutive HSP90 expression. Tubulin or actin levels serve as loading controls. WT indicates the wild type, untagged control. Three biological replicates are shown. (TIF) [file pgen.1007270.s015.tif]

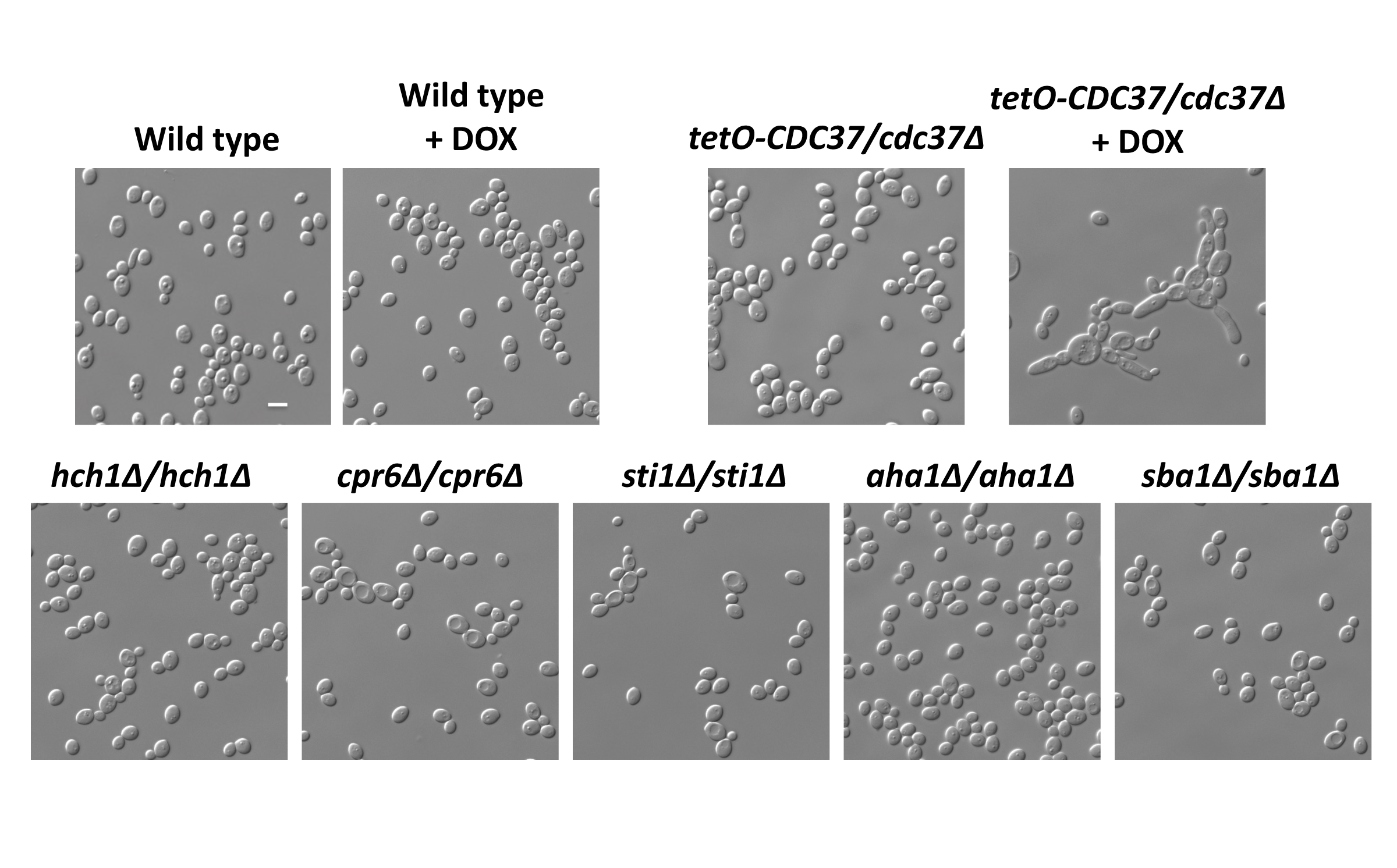

Supplement: S9 Fig — Deletion or depletion mutants were made for the six Hsf1-dependent Hsp90 co-chaperone genes in C. albicans. Strains were grown in the absence or presence of high DOX (20 μg/mL) at 30°C as indicated. Scale bar represents 10 μm. (TIF) [file pgen.1007270.s016.tif]

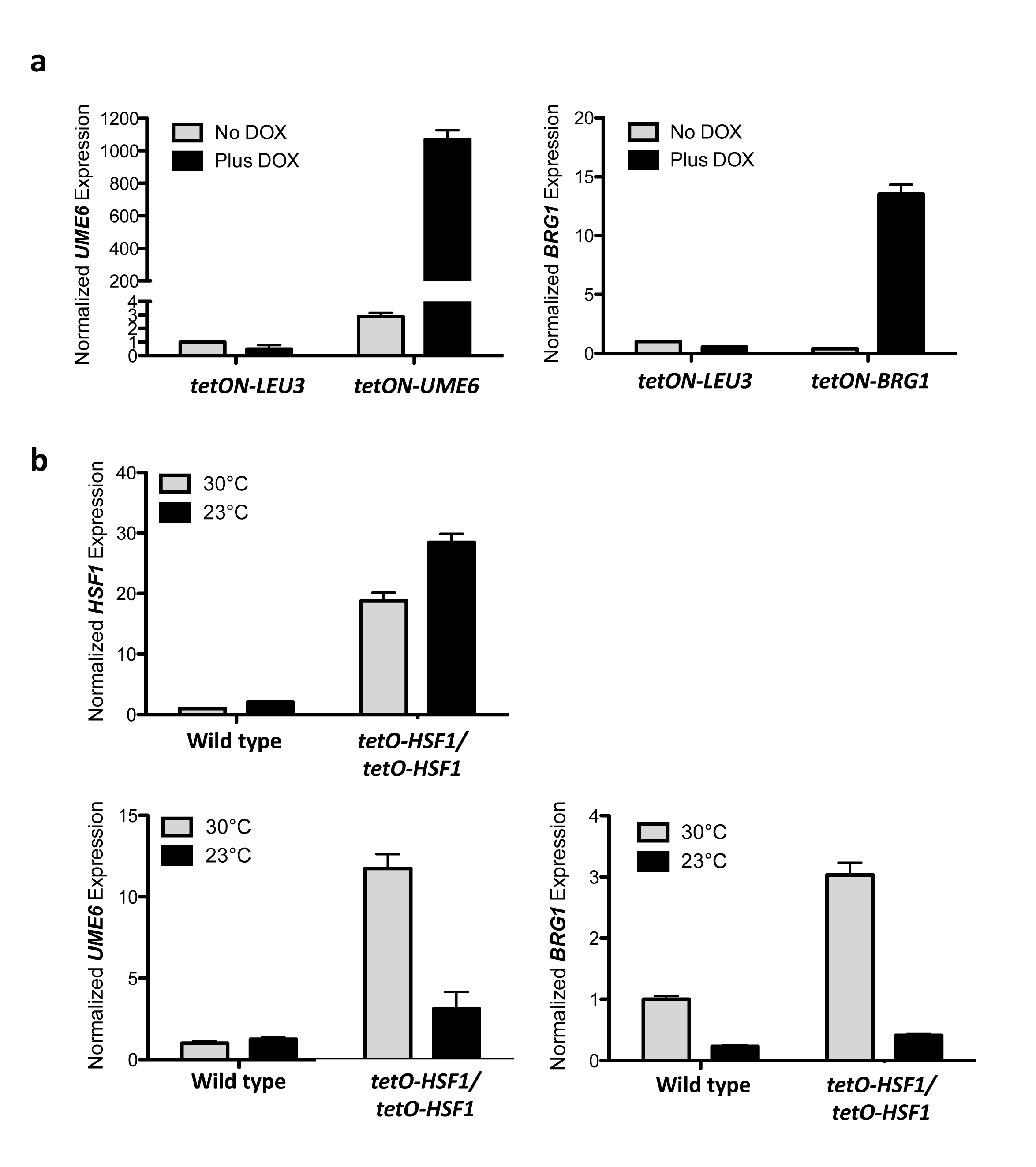

Supplement: S10 Fig — a) Quantitative RT-PCR analysis to validate the overexpression of UME6 or BRG1 in the tetracycline-inducible promoter (tetON) strains. Strains were grown in the absence or presence of 50 μg/mL DOX at 30°C to induce overexpression. Overexpression of LEU3 acts as a negative control. b) Overexpression of HSF1 no longer drives the overexpression of UME6 or BRG1 when grown at lower temperatures. The wild-type and tetO-HSF1/tetO-HSF1 strains were grown at 30°C or 23°C, in the absence of DOX, until mid-log phase. UME6, BRG1 and HSF1 transcript levels were normalized to ACT1 and GPD1. Data are means +/- standard error of the means for triplicate samples. (TIF) [file pgen.1007270.s017.tif]
